# Vacuolar protein sorting-associated protein 72 homolog (VPS72) binding to lysine acetyltransferase 5 (KAT5) promotes the proliferation, invasion and migration of hepatocellular carcinoma through regulating phosphatidylinositol 3-kinase (PI3K)/protein kinase B (AKT) signaling pathway

**DOI:** 10.1080/21655979.2022.2056692

**Published:** 2022-04-06

**Authors:** Tiejun Chen, Yinuo Tu, Dongnuo Lv, Kunpeng Lin, Hui Tang, Wenjie Huang

**Affiliations:** Department of Hepatobiliary Surgery, The Affiliated Cancer Hospital & Institute of Guangzhou Medical University, Guangzhou, Guangdong, P.R. China

**Keywords:** VPS72, KAT5, hepatocellular carcinoma, PI3K/AKT, proliferation

## Abstract

Hepatocellular carcinoma, a fatal malignancy that occurs in the liver, poses a major public health challenge. This paper attempted to clarify the role and mechanism of vacuolar protein sorting-associated protein 72 homolog (VPS72) in the progression of hepatocellular carcinoma. Firstly, VPS72 expression in hepatocellular carcinoma tissues and the prognostic correlation were analyzed by GEPIA2 database. Western blotting and RT-qPCR assays were used to evaluate VPS72 expression in several hepatocellular carcinoma cell lines. Then, cell proliferation was assessed by cell counting kit-8 and colony formation in HuH-7 cells with VPS72 silencing. Measurement of cell invasion and migration by transwell and wound healing assays. Next, the relationship between VPS72 and lysine acetyltransferase 5 (KAT5) was predicted by bioGRID, STRING and GEIPA2 databases, which was confirmed by Co-immunoprecipitation assay. Subsequently, KAT5 was overexpressed to explore whether VPS72 could regulate the progression of hepatocellular carcinoma by binding to KAT5. And the expression of proteins related to PI3K/AKT signaling was tested with western blotting. Results indicated that VPS72 was highly expressed in hepatocellular carcinoma tissues and cell lines and was associated with poor prognosis. VPS72 knockdown inhibited the proliferation, invasion and migration of HuH-7 cells. In addition, VPS72 could bind to KAT5. KAT5 overexpression reversed the suppressive impacts of VPS72 knockdown on the proliferation, invasion and migration in HuH-7 cells. Besides, VPS72 silencing downregulated p-PI3K and p-AKT expression, which was restored by KAT5 overexpression. Collectively, VPS72 binding to KAT5 promotes the progression of hepatocellular carcinoma through the regulation of PI3K/AKT signaling pathway.

## Graphical Abstract

**Figure:**
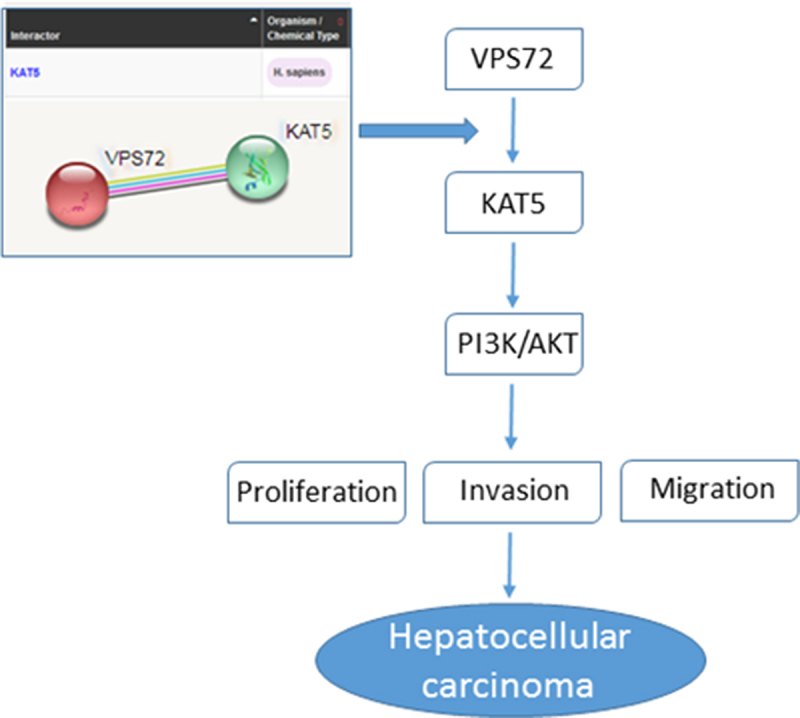

## Introduction

Primary hepatocellular carcinoma is the fifth leading cancer in the world and a significant source of cancer-related deaths worldwide [1]. The main reason for this is its poor prognosis. Only 5% to 15% of early-stage patients can be treated by surgical resection. Late treatment is then based on transarterial chemoembolization (TACE) or orally administered sorafenib, but less than one-third of patients benefit from it [2]. Although improvements in early diagnosis have led to effective treatment, the five-year survival rate remains relatively low due to its high recurrence rate of 70% after conventional treatment [3,4]. It is well known that tumor cell proliferation, invasion and metastasis are the main factors for cancer recurrence and poor prognosis. Much of the current literature have also paid particular attention to effective molecularly targeted therapies for hepatocellular carcinoma from this perspective. For example, Kou J-T et al. noted that the downregulation of lncRNA NEAT1 expression can facilitate cell apoptosis and inhibit cell proliferation and invasion of hepatocellular carcinoma cells [5]. Surveys such as that conducted by Aran G et al. have shown that CD5L could promote hepatocellular carcinoma cell proliferation and antiapoptotic response by binding to HSPA5 (GRP78) [6]. Zha et al. argued that miRNA-125a-5p regulates hepatocellular carcinoma cell growth, migration and invasion by targeting hematopoietic-substrate-1-associated protein X-1 (HAX1) [7].

Vacuolar protein sorting-associated protein 72 homolog (VPS72) is mainly located in the nucleus as a component of the SRCAP complex, mediating the exchange of ATP-dependent histone H2AZ1/H2B dimer with nucleosome H2A/H2B, and leading to transcriptional regulation of selected genes through chromatin remodeling [8]. Up to now, much less is known about VPS72, and only a few articles have reported that VPS72/YL1-mediated H2A.Z deposition is a necessary process for nuclear recombination to occur after mitosis [9]. Moreover, Han et al. discovered that Secretory Carrier Membrane Proteins (SCAMP) 3 is overexpressed in hepatocellular carcinoma and is involved in regulating VPS72 gene expression, which in turn affects cell adhesion, proliferation, transcription, cycle and metabolism [8]. Online analysis by the Gene Expression Profiling Interactive Analysis (GEPIA2) database (http://gepia2.cancer-pku.cn/#index) showed that VPS72 was highly expressed in patients with hepatocellular carcinoma and its high expression correlated with poor patient prognosis. To clarify the mechanism of action of VPS72 in hepatocellular carcinoma, according to the ranking of VPS72 expression in hepatocellular carcinoma patients on The Cancer Genome Atlas (TCGA) database (https://portal.gdc.cancer.gov/), Gene Set Enrichment Analysis (GSEA) enrichment analysis revealed that VPS72 was mainly enriched in some specific signaling pathways. These findings implied that the expression of VPS72 may be related to the proliferation of hepatocellular carcinoma cells.

Furthermore, bioGRID database (https://thebiogrid.org/) and STRING database (http://string-db.org) analysis suggested that VPS72 may interact with lysine acetyltransferase 5 (KAT5), which is reported to be involved in cancer progression. For instance, KAT5 negatively regulated the growth of prostate cancer cells [10]. KAT5/transformation/transcription domain-associated protein (TRRAP) exerted a role in promoting hepatocellular carcinoma cell proliferation by activating mitotic genes [11]. What’s more, KAT5 and KAT6B positively regulated prostate cancer cell proliferation through phosphatidylinositol 3-kinase (PI3K)/protein kinase B (Akt) signaling pathway, which was aberrantly activated in hepatocellular carcinoma [12]. Collectively, it is speculated that VPS72 may bind to KAT5 to promote proliferation, invasion and migration of hepatocellular carcinoma cells by regulating PI3K/AKT signaling pathway.

In this study, the expression of VPS72 in hepatocellular carcinoma tissues and overall survival were analyzed using GEPIA2 database. The functions of VPS72 on malignant biological properties of hepatocellular carcinoma cells were explored in the subsequent experiments. The potential mechanism between VPS72 and KAT5 in the progression hepatocellular carcinoma was further investigated. Our findings might offer a novel and promising target for the treatment of hepatocellular carcinoma.

## Materials and methods

### Bioinformatics analysis

VPS72 expression in liver hepatocellular carcinoma (LIHC) tissues and overall survival rate were analyzed by GEPIA2 database (http://gepia.cancer-pku.cn/). The correlation analysis between VPS72 and KAT5 expression in LIHC tissues was also analyzed by GEPIA2 database. TCGA datasets were downloaded from the public database cBioportal (http://www.cbioportal.org/) and Kyoto Encyclopedia of Genes and Genomes (KEGG) enrichment performed by Sangerbox (http://sangerbox.com/), a comprehensive tool for bioinformatics analysis based on R. The interaction between VPS72 and KAT5 was analyzed by bioGRID database (https://thebiogrid.org/) and STRING database (http://string-db.org).

### Cell culture

The human immortalized liver cell lines MIHA, human hepatocellular carcinoma cells lines Hep10, HuH-7 and SNU-387 were provided by Biovector NTCC Inc. (Beijing, China). MIHA and SNU-387 cells were cultivated in Roswell Park Memorial Institute (RPMI)-1640 medium (Procell, Wuhan, China) with 10% fetal bovine serum (FBS; RWD Life Science, Shenzhen, China). HuH-7 cells and Hep10 cells were grown in Dulbecco’s Modified Eagle Medium (DMEM) with 10% FBS for cultivation. The culture conditions for all cells were 37°C, 5% CO_2_ and 95% air.

### Cell transfection

Short hairpin RNA (shRNA) targeting VPS72 (sh-VPS72#1/2) and the negative control (sh-NC), KAT5 overexpression vector (oe-KAT5) and the empty vector plasmid (oe-NC) were designed and offered by Shanghai GeneChem Co., Ltd. The vectors were transfected into HuH-7 cells separately with the application of Lipofectamine^TM^ 2000 (Invitrogen, Carlsbad, CA, USA) for 48 h. The transfection efficiency of VPS72 and KAT5 was identified by the way of reverse transcription-quantitative PCR (RT-qPCR) and western blot analysis.

### Western blot assay

Protein extraction from HUH-7 cells treated in different groups was conducted by ice-cold radioimmunoprecipitation (RIPA) lysis buffer for 15 min and centrifuged at 4°C with 13,000 × g for 10 min. The harvest total protein concentration was measured by the bicinchoninic acid assay (BCA; TW-reagent, Shanghai, China) strictly based on manual provided by the manufacturer. The separation of equal amounts of 20 µg protein samples was carried out adopting 10% sodium dodecyl sulfate-polyacrylamide gel electrophoresis (SDS-PAGE) and transferred to nitrocellulose membranes. The membranes were blocked adopting 5% nonfat dry milk before the incubation with the primary antibodies overnight at 4°C. The primary antibodies used here were against VPS72 (Abcam, ab112055, 1:1000), proliferating cell nuclear antigen (PCNA; Abcam, ab18197, 1:1000), Ki67 (Beyotime, AF1738, 1:1000), matrix metallopeptidase 2 (MMP2; Abcam, ab97779, 1:500), matrix metallopeptidase 9 (MMP9; Abcam, ab38898, 1:1000), KAT5 (Abcam, ab137518, 1:1000), PI3K (Abcam, ab227204, 1:1000), phosphorylated (p)-PI3K (Abcam, ab278545, 1:1000), AKT (Cell Signaling Technology, #9272S, 1:1000), and p-AKT (Abcam, ab38449, 1:1000) and GAPDH (Cell Signaling Technology, #5174 T, 1:1000). Then, the membranes were interacted with horseradish peroxidase-conjugated (HRP) IgG secondary antibody (Abcam, ab205718, 1:10,000) for 1 h at room temperature. The visualization of protein bands employed an enhanced chemiluminescence (ECL) reagent (Beyotime, Shanghai, China). The blots were captured with the aid of Image Pro Plus software (Media Cybernetics, Rockville, MD, USA).

### RT-qPCR assay

The RT-qPCR was carried out for the measurement of VPS72 and KAT5 mRNA expression. The isolation of the whole RNA from HuH7 cells was performed utilizing TRIzol reagent (Invitrogen) in line with the recommendations mentioned by vendor. The synthesis of the complementary DNA (cDNA) from the total RNA was run with the help of the PrimeScriptTM RT Reagent Kit (Takara, Tokyo, Japan). The quantitation reaction was done using the SYBR Prime Script RT-PCR Kit on an ABI 7500 quantitative PCR instrument (ABI/Perkin Elmer, CA, USA). The primer sequences used in this study were displayed as follows: VPS72, forward: 5’-GAGGAATGGTTCCCCCAAGG-3’, reverse: 5’-TCCCAACCCTAGACTGGACA-3’; KAT5, forward: 5’-AGTGGAGGGAGGGAAGATGG-3’, reverse: 5’-TCTTCGTTGTCCTGGTTCCG-3’; Glyceraldehyde-phosphate dehydrogenase (GAPDH), forward: 5’-ACAACTTTGGTATCGTGGAAGG-3’ and reverse: 5’- GCCATCACGCCACAGTTTC-3’. GAPDH acted as an endogenous reference. The calculation of the relative gene expression was verified by the 2^−ΔΔCT^ method [13].

### Cell counting kit (CCK)-8 assay

The CCK-8 method was adopted to identify the proliferation of HuH7 cells. Cells in different groups were cultured in 96-well plates, each well containing approximately 10,000 cells. Then, the wells were added with 10 μL CCK-8 solutions (Yeasen, Shanghai, China) for 4 h incubation. Finally, a microplate reader (Bio-Rad, Hercules, CA, USA) was utilized to test the optical density value at 490 nm.

### Colony formation method

For the colony formation assay, HuH7 cells were seeded into 6-well plates and incubated for 12 days. Cells were washed with precooled phosphate buffer solution (PBS) twice, followed by a 5 min fixation of 4% paraformaldehyde at room temperature as well as a 10 min staining of 0.1% crystal violet. Finally, the number of colonies more than 50 cells per colony was counted.

### Transwell assay

The invasion ability of HuH7 cells was performed with the adoption of transwell method. Generally, the Matrigel (BD, Franklin Lakes, NJ, USA) was pre-coated onto the upper chamber of the transwell (BD). HuH7 cells were suspended in 200 μL of serum-free medium were added to the upper layer of transwell chambers. The lower chamber was added with 600 μL of medium containing 10% FBS. Next, the cells not crossing membranes were taken out with a cotton swab. The cells passed the membranes were fixed and stained. A light microscope (Nikon, Tokyo, Japan) was applied to count these cells in 5 fields selected randomly.

### Wound healing assay

The migration ability of HuH7 cells was evaluated by means of wound healing assay. For a brief overview, cells were maintained in 6-well plates until reaching a confluence of 95%. Then, 200 μL of sterile pipette tip was employed to scratch the cell surface. Cell were washed with PBS for three times before the cultivation together with the serum-free medium under the same conditions for 24 h. Finally, the images of cell migration at 0 and 24 h were captured by a light microscope (Nikon, Tokyo, Japan) and the migration length was calculated.

### Co-immunoprecipitation (Co-IP) experiment

To detect the interaction between VPS72 and KAT5, the steps of Co-IP were carried out as follows: HuH7 cells were first lysed in RIPA buffer supplemented with protease inhibitor cocktail (GlpBio technology, Montclair, CA, USA) for 30 min and centrifuged at 13,000 g for 5 min at 4°C. The collected supernatants were incubated with corresponding antibodies overnight at 4°C. Next, the 40 μL of protein A + G Agarose was added for 2 h incubation. After washing with PBS and boiled for 5 min, the 40 μL of protein samples were collected for the detection of western blot.

### Statistical analysis

Experiment data were documented in the way of mean ± standard deviation (SD) and analyzed with GraphPad Prism 8.0 (GraphPad Software, Inc.). The Overall Survival was calculated by Kaplan-Meier survival analysis. Student’s t-test was applied for the comparison between the two groups and one-way analysis of variance (ANOVA) for the difference among more than three groups. It was statistically significant when p-value was less than 0.05.

## Results

### VPS72 is highly expressed in hepatocellular carcinoma tissues and cell lines and correlates with poor prognosis

**Figure 1.**
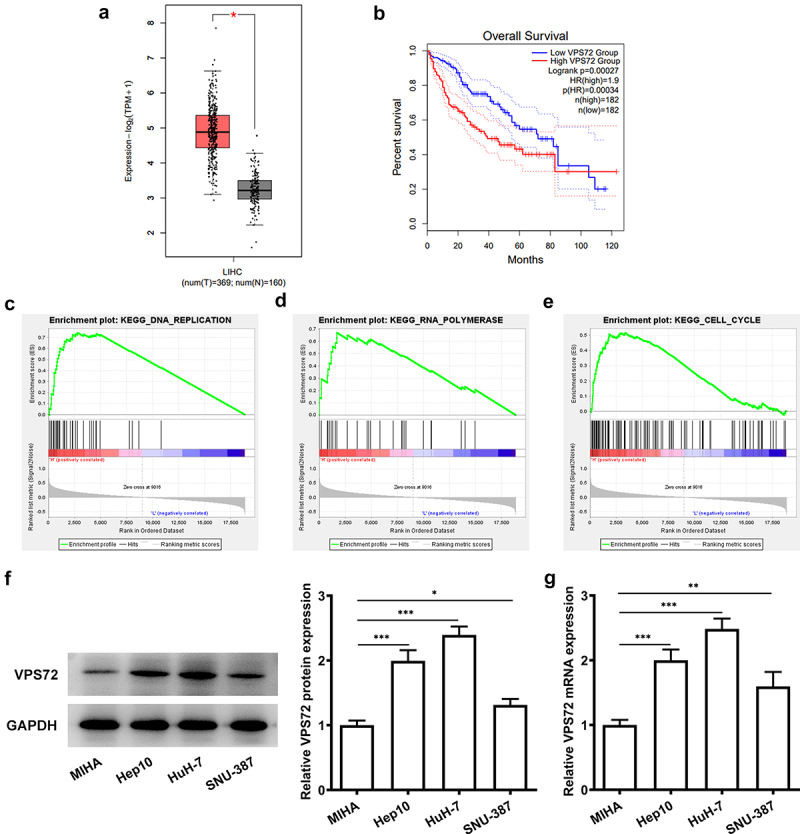
VPS72 was highly expressed in hepatocellular carcinoma tissues and cell lines and correlated with poor prognosis. (a-b) High expression of VPS72 in hepatocellular carcinoma tissues and low overall survival of patients with hepatocellular carcinoma were showed on GEPIA2 database. (c-e) Major signaling pathways where VPS72 was enriched were presented on GSEA software. (f-g) Protein and mRNA expression levels of VPS72 were tested by western blot and RT-qPCR in the human immortalized liver cell lines MIHA, human hepatocellular carcinoma cells lines Hep10, HuH-7 and SNU-387. Results were generated from three independent experiments and data were expressed as mean ± standard deviation (SD). *P < 0.05, **P < 0.01, ***P < 0.001.

To verify the specific role of VPS72 in hepatocellular carcinoma, the prediction of VPS72 expression was performed on the GEPIA2. On the basis of the results in Figure 1a-b, VPS72 expression was upregulated in the hepatocellular carcinoma tissues (P < 0.05) and high VPS72 expression was correlated with the low overall survival of patients (P = 0.00027). In addition, GSEA database showed that VPS72 was mainly enriched in the signaling pathways such as DNA replication (ES: 0.7447, P = 0.008, FDR = 0.0355), RNA polymerase (ES: 0.6708, P < 0.001, FDR = 0.0336), and cell cycle (ES: 0.5163, P < 0.023, FDR = 0.0978) (Figure 1c-e). Subsequent western blot and RT-qPCR assays in figure 1f-g displayed higher protein and mRNA levels of VPS72 in human hepatocellular carcinoma cell lines Hep10, HuH7 and SNU-387 than those of the human immortalized liver cell lines MIHA, especially the highest expression in HuH7 cells. As a result, HuH7 cells were selected as the subject for the next experiments. Together, these experiments results indicate that VPS72 is highly expressed in the hepatocellular carcinoma tissues and cell lines and high VPS72 expression is implicated in poor prognosis.

### VPS72 knockdown inhibits the proliferation of HuH7 cells

**Figure 2.**
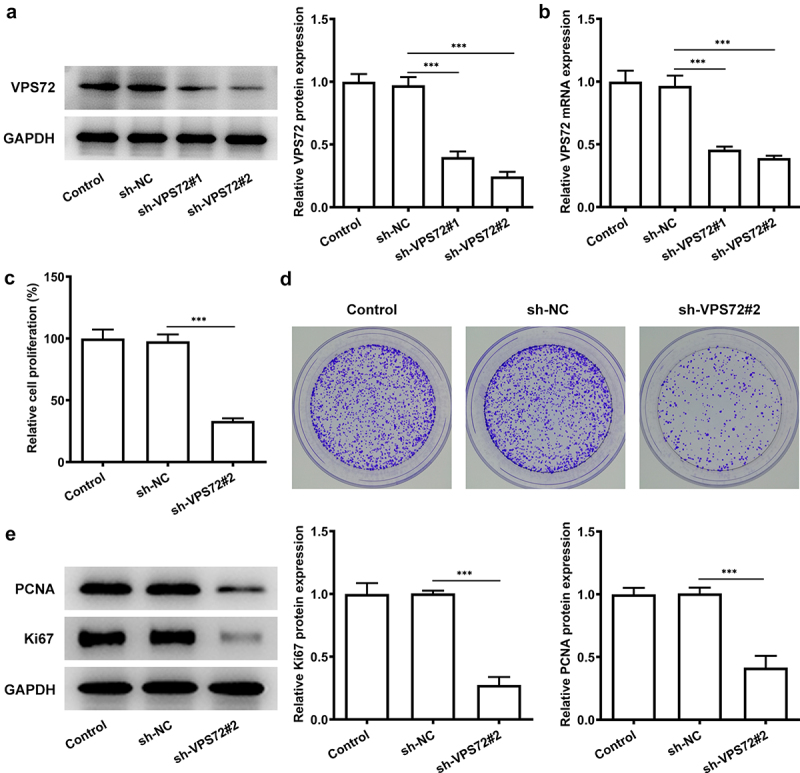
VPS72 knockdown inhibited the proliferation of HuH7 cells. (a-b) Protein and mRNA expression levels of VPS72 in HuH7 cells were tested by western blot and RT-qPCR in the groups of control, sh-NC, and sh-VPS72#1/2. (c) The proliferation of HuH7 cells was detected by means of CCK-8 in the groups of control, sh-NC, and sh-VPS72#2. (d) Cell proliferation was examined by colony formation assay in HuH7 cells in the groups of control, sh-NC, and sh-VPS72#2. (e) Protein expression levels of MMP2 and MMP9 in HuH7 cells were detected by western blot in HuH7 cells in the groups of control, sh-NC, and sh-VPS72#2. Data were obtained from three independent experiments and expressed as mean ± standard deviation (SD). ***P < 0.001.

For the sake of figuring out whether VPS72 has an effect on the proliferative ability of hepatocellular carcinoma cells, HuH7 cells were transfected with sh-VPS72#1 and sh-VPS72#2 to silence VPS72 expression. Then, the expression of VPS72 was examined by the ways of western blot and RT-qPCR. Figure 2a-b presented a declined expression of VPS72 in the groups of sh-VPS72#1/2 (vs sh-NC), and VPS72 expression was lower in the sh-VPS72#2 than that in the sh-VPS72#1, and therefore the later was used for the next experiments. Afterward, the impact of VPS72 knockdown on HuH7 cell proliferation was examined. As shown in Figure 2c, the relative cell proliferation percenter dropped sharply after VPS72 knockdown (vs sh-NC). It could be found from the Figure 2d that the number of colonies declined steeply in the sh-VPS72#2 group compared with the sh-NC group. Additionally, data exhibited in Figure 2e indicated that the protein expression of PCNA and Ki67 related to proliferation in HuH7 cells was remarkably reduced after transfection with sh-VPS72#2 (vs sh-NC). Overall, these results highlight the fact that VPS72 knockdown can effectively inhibit the proliferation of hepatocellular carcinoma cells.

### VPS72 knockdown inhibits the invasion and migration of HuH7 cells

**Figure 3.**
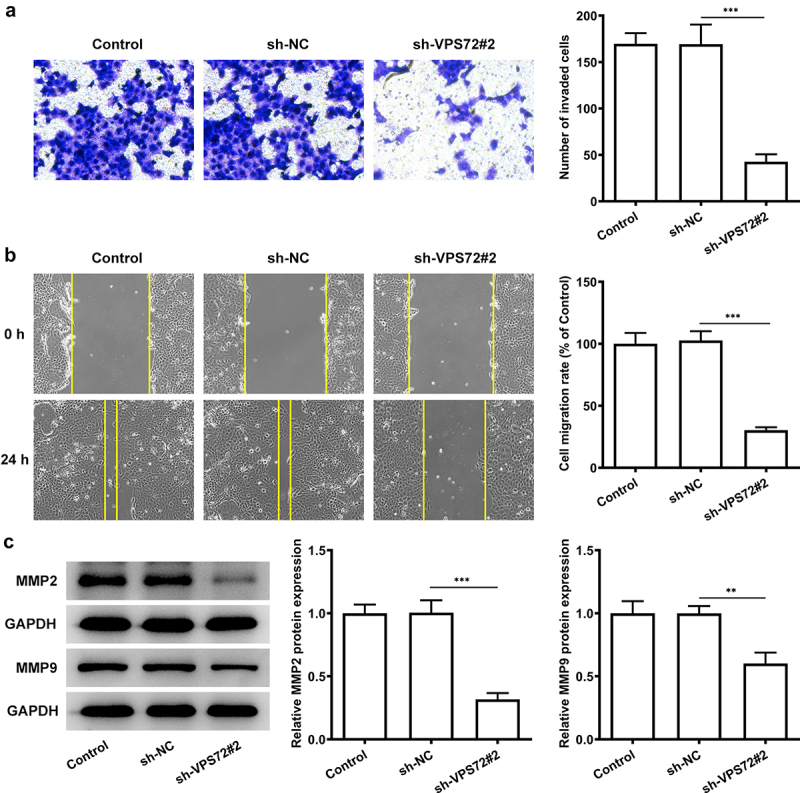
VPS72 knockdown suppressed the invasion and migration of HuH7 cells. (a) Cell invasion capacity of HuH7 cells was measured by transwell in the groups of control, sh-NC, and sh-VPS72#2. (b) Cell migration ability of HuH7 cells was detected by wound healing in the groups of control, sh-NC, and sh-VPS72#2. (c) Protein expression levels of MMP2 and MMP9 in HuH7 cells were examined by western blot in the groups of control, sh-NC, and sh-VPS72#2. Results were generated from three independent experiments and data were expressed as mean ± standard deviation (SD). **P < 0.01, ***P < 0.001.

To fully confirm the role of VPS72 knockdown on hepatocellular carcinoma cells, the changes in invasion and migration ability of HuH7 cells after VPS72 knockdown were detected. It was apparent from the Figure 3a that very few invaded cells were observed in the sh-VPS72#2 group (vs sh-NC). The cell migration rate in Figure 3b was obviously decreased in the sh-VPS72#2 group (vs sh-NC). What could be clearly seen in this Figure 3c was that markedly decreased protein expression of matrix MMP2 and MMP9 after transfected with sh-VPS72#2, by contrast with the negative control group. Considering all of the evidence, it seems that VPS72 knockdown also suppressed the invasion and migration of HuH7 cells.

### VPS72 can interact with KAT5 in hepatocellular carcinoma cells

**Figure 4.**
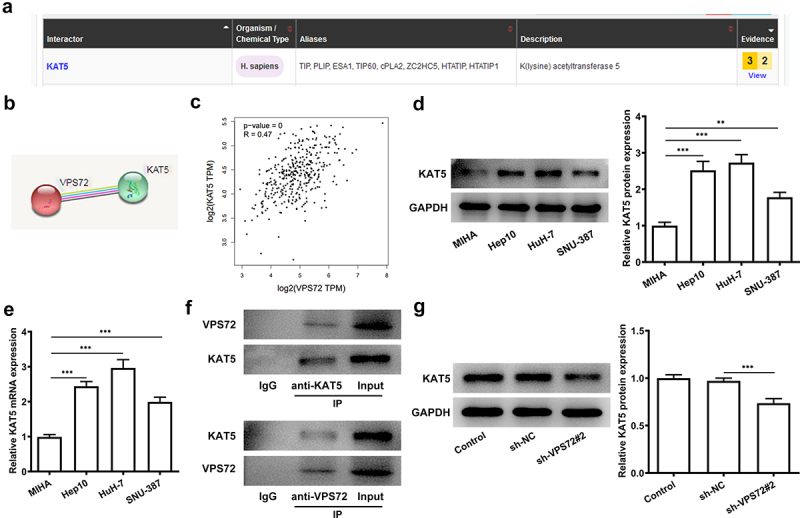
VPS72 could interact with KAT5 in hepatocellular carcinoma cells. (a-b) The interaction of VPS72 and KAT5 was analyzed by bioGRID database and STRING database, respectively. (c) The correlation analysis between VPS72 and KAT5 expression in hepatocellular carcinoma tissues was analyzed by GEPIA2 database. (d-e) Protein and mRNA expression levels of KAT5 were detected by western blot and RT-qPCR in the human immortalized liver cell lines MIHA, human hepatocellular carcinoma cells lines Hep10, HuH-7 and SNU-387. (f) The targeted binding of KAT5 and VPS72 was confirmed by Co-IP. (g) KAT5 expression in HuH7 cells in the groups of control, sh-NC, and sh-VPS72#2 was tested by western blot after VPS72 knockdown. Experiments were repeated three times and the measurement data were expressed in the form of the mean ± standard deviation (SD). **P < 0.01, ***P < 0.001.

To explore the potential mechanisms of VPS72 on the regulation of hepatocellular carcinoma progression, the relationship between of VPS72 and KAT5 was analyzed by using the bioGRID, STRING and GEPIA2 databases. The interaction between VPS72 and KAT5 was predicated by bioGRID database and STRING database, and the results were respectively exhibited in Figure 4a-b. Furthermore, from the Figure 4c, it could be seen that expression of VPS72 in hepatocellular carcinoma tissues was positively correlated with KAT5. KAT5 was highly expressed in the hepatocellular carcinoma cells lines Hep10, HuH-7 and SNU-387, and showed the highest expression in HuH7 cells (Figure 4d-e). Co-IP experiment also confirmed the targeting binding of VPS72 and KAT5 (figure 4f). Additionally, KAT5 expressed a lower level in HuH7 cells transfected with sh-VPS72#2 (vs sh-NC; Figure 4g), which illustrated that VPS72 knockdown could inhibit the expression of KAT5 in HuH7 cells. Collectively, this evidence outlines a critical targeted relationship of VPS72 and KAT5.

### Overexpression of KAT5 reverses the inhibitory effect of VPS72 knockdown on the proliferation of HuH7 cells

**Figure 5.**
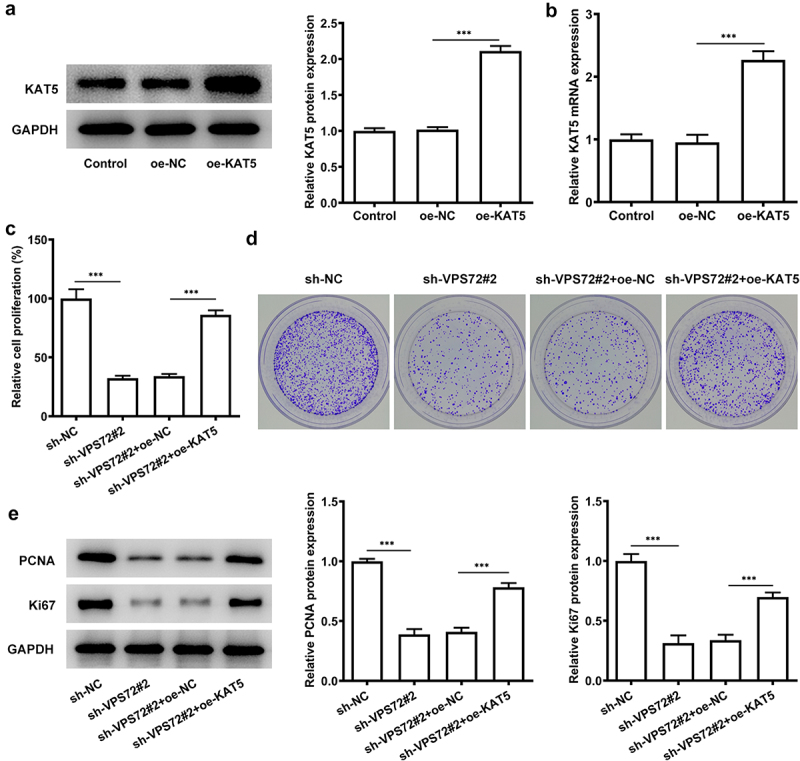
Overexpression of KAT5 reversed the inhibitory effect of VPS72 knockdown on the proliferation in HuH7 cells. (a-b) KAT5 expression in HuH7 cells transfected with oe-KAT5 was tested by RT-qPCR and western blot in the groups of control, oe-NC and oe-KAT5. (c) The proliferation rate of HuH7 cells was detected by means of CCK-8 in the groups of sh-NC, sh-VPS72#2, sh-VPS72#2+ oe-NC and sh-VPS72#2+ oe-KAT5. (d) Measurement of cell proliferation in HuH7 cells in the groups of sh-NC, sh-VPS72#2, sh-VPS72#2+ oe-NC and sh-VPS72#2+ oe-KAT5 with the use of colony formation assay. (e) Protein expression levels of MMP2 and MMP9 in HuH7 cells were detected by western blot in HuH7 cells in the groups of sh-NC, sh-VPS72#2, sh-VPS72#2+ oe-NC and sh-VPS72#2+ oe-KAT5. Results were generated from three independent experiments and data were expressed as mean ± standard deviation (SD). ***P < 0.001.

VPS72 knockdown has previously been demonstrated to have an inhibitory effect on HuH7 cell proliferation and VPS72 is associated with KAT5. This section was designed to explore whether KAT5 overexpression could influence the outcome of VPS72 knockdown in HuH7 cells. From the Figure 5a-b, the protein and mRNA expression of KAT5 was rapidly elevated after KAT5 overexpression (vs oe-NC). In Figure 5c, there was a clear trend of increasing in the proliferation rate of HuH7 cells co-transfected with sh-VPS72#2 and oe-KAT5 in comparison with the overexpression negative control. Moreover, KAT5 overexpression increased the number of colonies of HuH7 cells transfected with sh-VPS72#2 (vs oe-NC; Figure 5d). Not only that, there were higher protein expression of PCNA and Ki67 in the sh-VPS72#2+ oe-KAT5 group that those of the sh-VPS72#2+ oe-NC group (Figure 5e). The above results suggest that overexpression of KAT5 can attenuate the suppressive effect of VPS72 knockdown on the proliferation of HuH7 cells.

### Overexpression of KAT5 reverses the suppressive impacts of VPS72 knockdown on the invasion and migration of HuH7 cells

**Figure 6.**
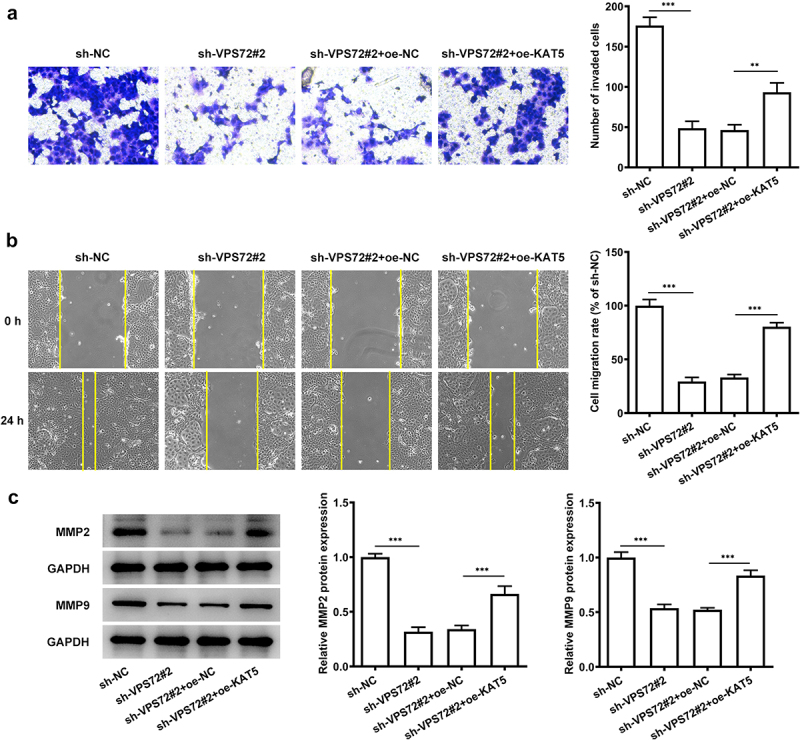
Overexpression of KAT5 restored the suppressive impacts of VPS72 knockdown on the invasion and migration of in HuH7 cells. (a) Cell invasion capacity of HuH7 cells was measured by transwell in the groups of sh-NC, sh-VPS72#2, sh-VPS72#2+ oe-NC and sh-VPS72#2+ oe-KAT5. (b) Cell migration ability of HuH7 cells was detected by wound healing in the groups of sh-NC, sh-VPS72#2, sh-VPS72#2+ oe-NC and sh-VPS72#2+ oe-KAT5. (c) Protein expression levels of MMP2 and MMP9 in HuH7 cells were examined by western blot in the groups of sh-NC, sh-VPS72#2, sh-VPS72#2+ oe-NC and sh-VPS72#2+ oe-KAT5. Data were obtained from three independent experiments and expressed as mean ± standard deviation (SD). **P < 0.01, ***P < 0.001.

This section set out to demonstrate if KAT5 overexpression could affect the inhibitory effects of VPS72 knockdown on the invasion and migration of HuH7 cells. As indicated in Figure 6a, KAT5 overexpression improved the invasion capacity of HuH7 cells transfected with sh-VPS72#2 (vs oe-NC). Also, the migration ability of HuH7 cells co-transfected with sh-VPS72#2 and oe-KAT5 was notably increased, compared with the overexpression negative control (Figure 6b). What’s more, the protein expression of MMP2 and MMP9 were also elevated in the sh-VPS72#2 and oe-KAT5 group (oe-NC) (Figure 6c). All of the results here support the fact that KAT5 overexpression attenuates the inhibitory effects of VPS72 knockdown on the invasion and migration of HuH7 cells.

### Overexpression of KAT5 cripples the impacts of VPS72 silencing on the PI3K/AKT signaling in HuH7 cells

**Figure 7.**
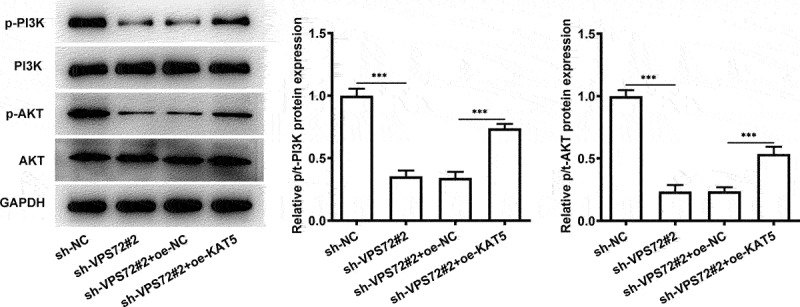
Overexpression of KAT5 crippled the impacts of VPS72 silencing on the PI3K/AKT signaling in HuH7 cells. Analysis of p-PI3K, PI3K, p-AKT and AKT expression using western blot analysis. Results were generated from three independent experiments and data were expressed as mean ± standard deviation (SD). ***P < 0.001.

To further explore the mechanisms of VPS72 binding to KAT5 in the regulation of hepatocellular carcinoma progression, the expression of proteins in the PI3K/AKT signaling was analyzed in the subsequent experiments. As displayed in Figure 7, VPS72 silencing notably downregulated p-PI3K and p-AKT expression when compared to the sh-NC group. However, the further gain-of-function of KAT5 conspicuously restored the inhibited p-PI3K and p-AKT expression induced by VPS72 deletion. These data suggest that VPS72 binds to KAT5, thereby regulating PI3K/AKT signaling pathway in HuH7 cells.

## Discussion

Hepatocellular carcinoma is a common malignant tumor in the digestive system with high mortality rate and poor prognosis [14]. Aberrant malignant cellular events including proliferation, migration and invasion may be implicated in the initiation and development of hepatocellular carcinoma [15]. In this study, both VPS72 and KAT5 were shown to be highly expressed in hepatocellular carcinoma cells, and their high expression was associated with poor prognosis. Efforts have been made in this paper and it was concluded that VPS72 knockdown inhibited the proliferation, invasion and migration of hepatocellular carcinoma cells. Targeted binding of VPS72 and KAT5 was also demonstrated. And overexpression of KAT5 reversed the inhibitory effects of VPS72 knockdown on the progression of HuH7 cells.

VPS72, also known as YL1 or Swc2, was originally defined as a binding protein of nuclear and DNA [16]. It can undergo nuclear recombination at the end of mitosis by mediating H2A.Z [9]. Additionally, a previous study noted that VPS72 may affect cell adhesion, proliferation, transcription, cell cycle and metabolism [8]. For example, VPS72 is present in Saccharomyces cerevisiae as an abnormal intracellular trafficking and abnormal vesicle morphology [17]. Forced expression of YL-1 protein suppresses the anchorage-independent growth of Kirsten sarcoma virus-transformed NIH3T3 cells [18]. In this article, what we explore is the role of VPS72 in hepatocellular carcinoma. In our study, by biological information analysis, we found that VPS72 was highly expressed in hepatocellular carcinoma tissues and was associated with poor prognosis. GSEA enrichment analysis showed that VPS72 was closely associated with DNA replication, RNA polymerase and cell cycle. Upregulated VPS72 expression in HuH7 cells confirmed by western blot and RT-qPCR also suggests that VPS72 expression is intimately implicated in the development of hepatocellular carcinoma.

Additionally, after VPS72 knockdown, relative cell proliferation rate and cell colonies were declined, which illustrated the fact that VPS72 knockdown made a great difference on the cell proliferation. PCNA is regarded as a target for inhibition and can shut down highly proliferative cells, leading to the development of cancer treatment [19]. One study noted that PCNA expression was upregulated in hepatocellular carcinoma patients [20]. In our study, PCNA expression was high in HuH7 cells but decreased after VPS72 knockdown. Not only that, Ki67 is a proliferation marker used to assess cell proliferation [21]. And high expression of ki67 was associated with poor long-term survival in liver tumors [22]. Our experiments results showed the decreased protein expression of PCNA and Ki67, which also demonstrated the inhibitory effects of VPS72 knockdown on the proliferation of HuH7 cells. Likewise, declined abilities of cell invasion and migration were also the evidence to demonstrate the inhibitory effects of VPS72 on HuU7 cells. It is also because MMPs are zinc-dependent protein hydrolases used for extracellular matrix degradation during cell invasion and migration [23]. Among them, MMP2 and MMP9 are involved in the metastasis of hepatocellular carcinoma [24]. In this study, downregulated MMP2 and MMP9 expression was observed after VPS72 knockdown, which indicated that VPS72 knockdown suppressed the migration of HuH7 cells.

On the other hand, further bioGRID database and STRING database analysis revealed the interaction of VPS72 with KAT5. KAT5 belongs to the multiple KAT family members and is a potential therapeutic for several cancers, such as hepatocellular carcinoma and mesothelioma [25]. Numerous studies have confirmed the function of KAT5 on cancer cell proliferation, invasion and migration. For examples, KAT5 could promote invasion and metastasis through C-MYC stabilization in anaplastic thyroid cancer [26]. Whereas, the inhibition of KAT5 markedly suppressed the proliferation and induced apoptosis of malignant pleural mesothelioma cells [27]. In addition, KAT5 knockdown specifically inhibits the proliferation of gallbladder carcinoma (GBC)-SD cells by casp9-mediated apoptosis [28]. Some researches claimed that the activation of PI3K/AKT signaling pathway has been implicated in the pathogenesis of hepatocellular carcinoma [29,30]. A case here is that maternally expressed gene 3 promotes hepatocellular carcinoma by activating PI3K/AKT signaling pathway through regulating AP1G1 [31]. While the inhibition of PI3K/AKT signaling pathway can suppresses the progression of hepatocellular carcinoma [32]. Moreover, one study also demonstrated that KAT5 or KAT6B knockdown could suppress the AKT/AKT signaling in prostate cancer cells [12]. All the information indicated that KAT5 may make an influence on hepatocellular carcinoma cells by PI3K/AKT signaling pathway. In our study, upregulated KAT5 expression in HuH7 cells and the targeting binding of KAT5 and VPS72 identified by Co-IP detection revealed the fact that KAT5 may bind to VPS72 to affect the progression of hepatocellular carcinoma. Subsequently, after KAT5 overexpression, cell proliferation, invasion and migration capacity that had decreased after VPS72 knockdown now increased again. These findings implied that overexpression of KAT5 reversed the suppressive impacts of VPS72 deletion on the proliferation, invasion and migration in HuH7 cells by regulating the PI3K/AKT signaling pathway.

However, there are two limitations exited in the present study. This study only analyzed VPS72 expression and its correlation with disease progression in hepatocellular carcinoma tissues using GEPIA2 database, which have not been validated in clinical hepatocellular carcinoma tissues collected from patients. Additionally, we only discussed the effects and regulatory mechanism of VPS72 and KAT5 in hepatocellular carcinoma cells. The further in vivo tumor model experiments will be performed in the future investigation to support the conclusion obtained in this study.

## Conclusion

Taken together, the studies presented thus far provide evidence that VPS72 binding to KAT5 promotes the proliferation, invasion and migration of hepatocellular carcinoma cells through the regulation of PI3K/AKT signaling pathway, which illustrated that VPS72 maybe the potential marker for the treatment of hepatocellular carcinoma.

## Supplementary Material

Supplemental Material

## Data Availability

All data included in this study are available upon request through contact with the corresponding author.
