# Supplementary figures and images for: Vacuolar protein sorting-associated protein 72 homolog (VPS72) binding to lysine acetyltransferase 5 (KAT5) promotes the proliferation, invasion and migration of hepatocellular carcinoma through regulating phosphatidylinositol 3-kinase (PI3K)/protein kinase B (AKT) signaling pathway
Source: Bioengineered. 2022 Apr 6;13(4):9197–210. doi: 10.1080/21655979.2022.2056692 (PMC9161877; doi:10.1080/21655979.2022.2056692)

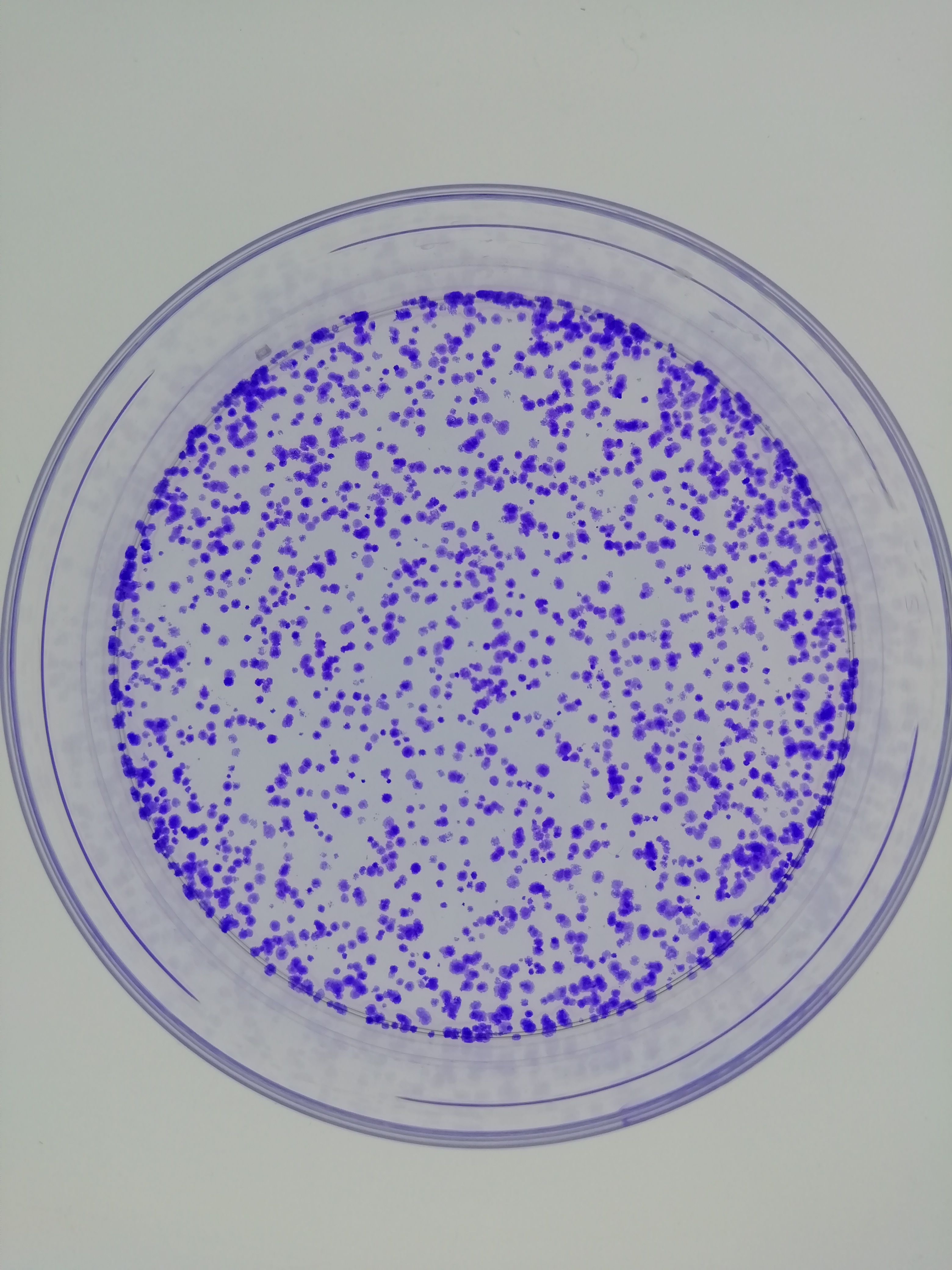

Supplement: Supplemental Material [file KBIE_A_2056692_SM9735.zip › supplementary/Fig2D_Control.jpg]

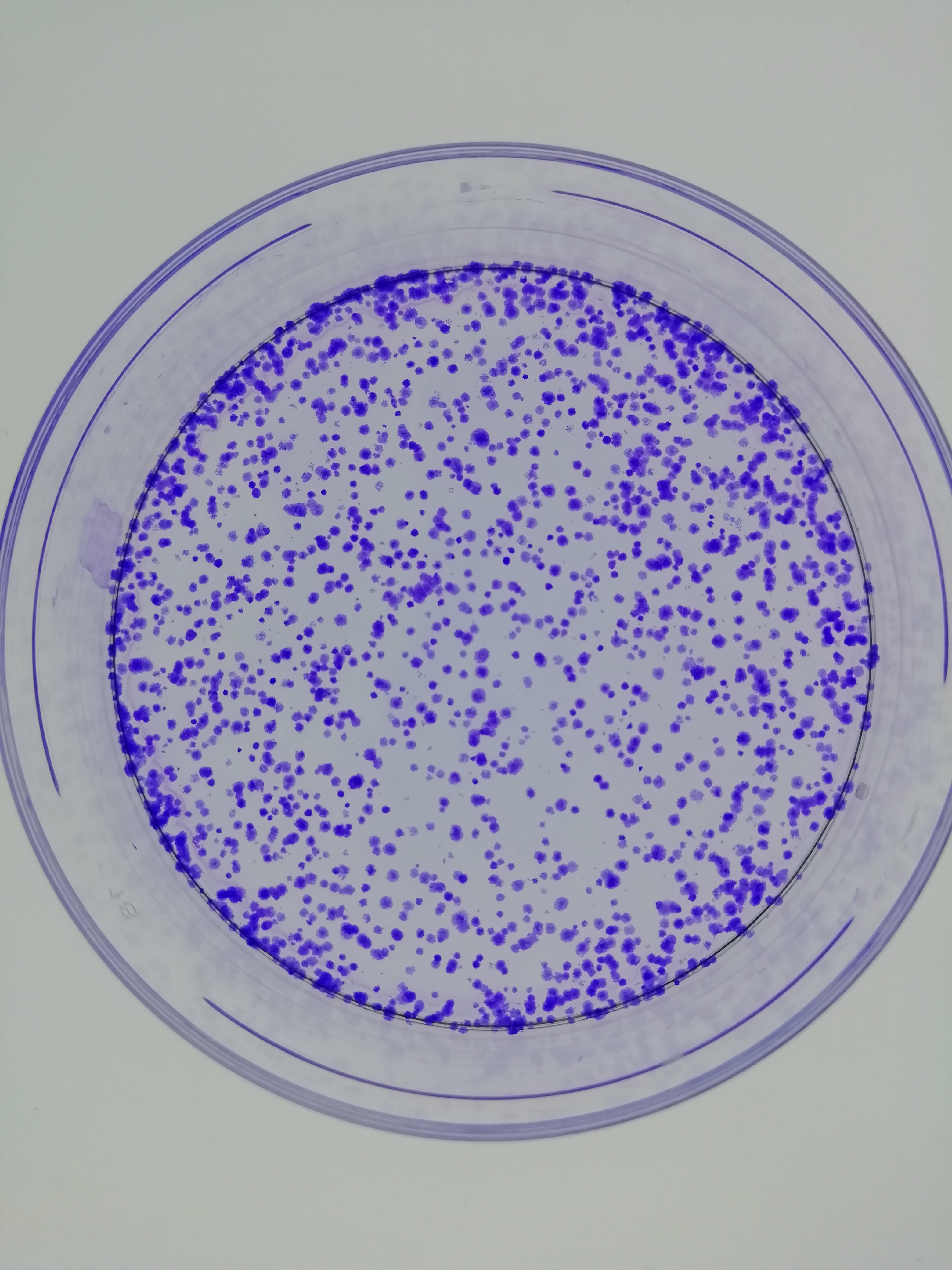

Supplement: Supplemental Material [file KBIE_A_2056692_SM9735.zip › supplementary/Fig2D_sh_NC.jpg]

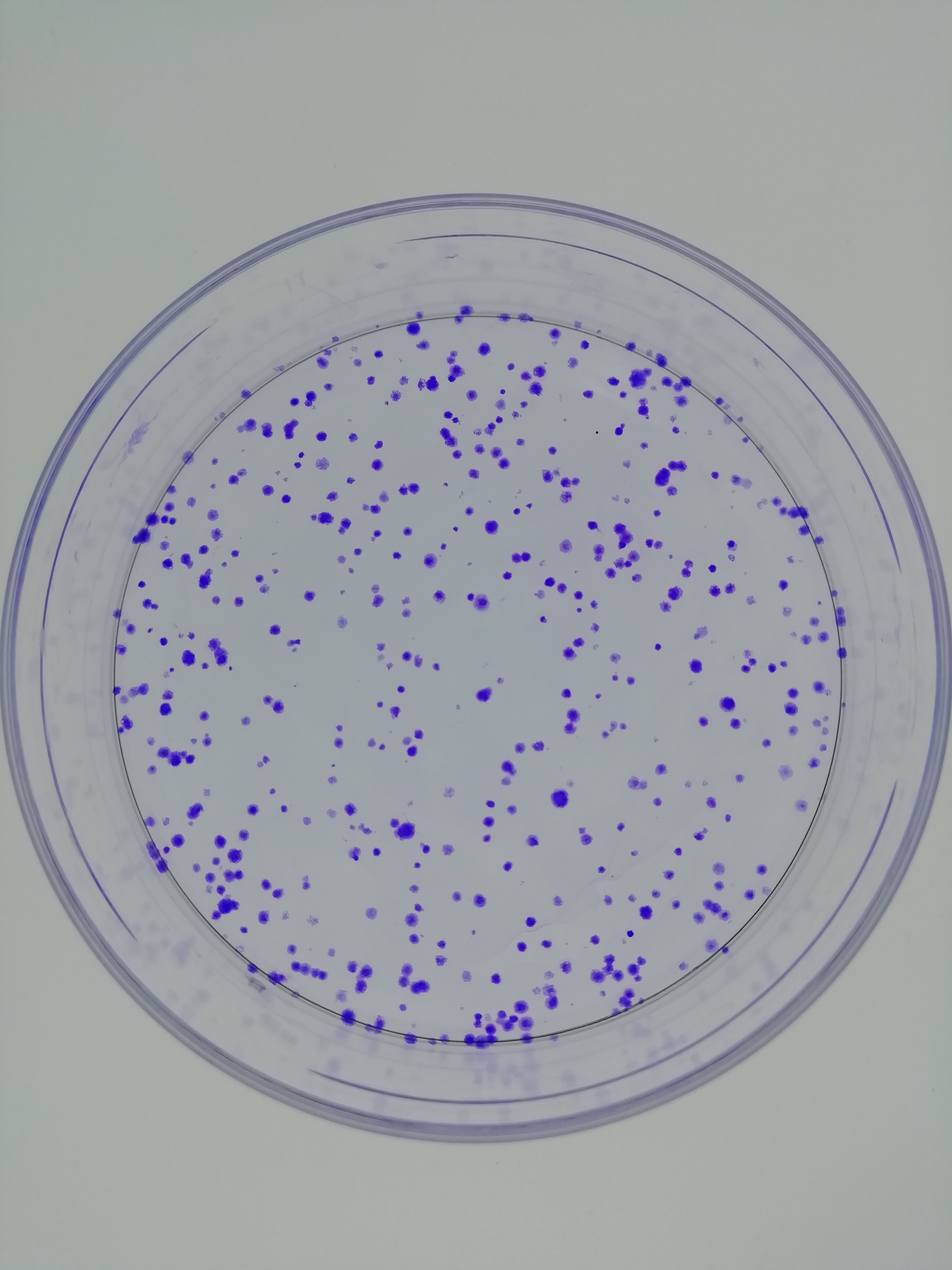

Supplement: Supplemental Material [file KBIE_A_2056692_SM9735.zip › supplementary/Fig2D_sh_VPS72_2.jpg]

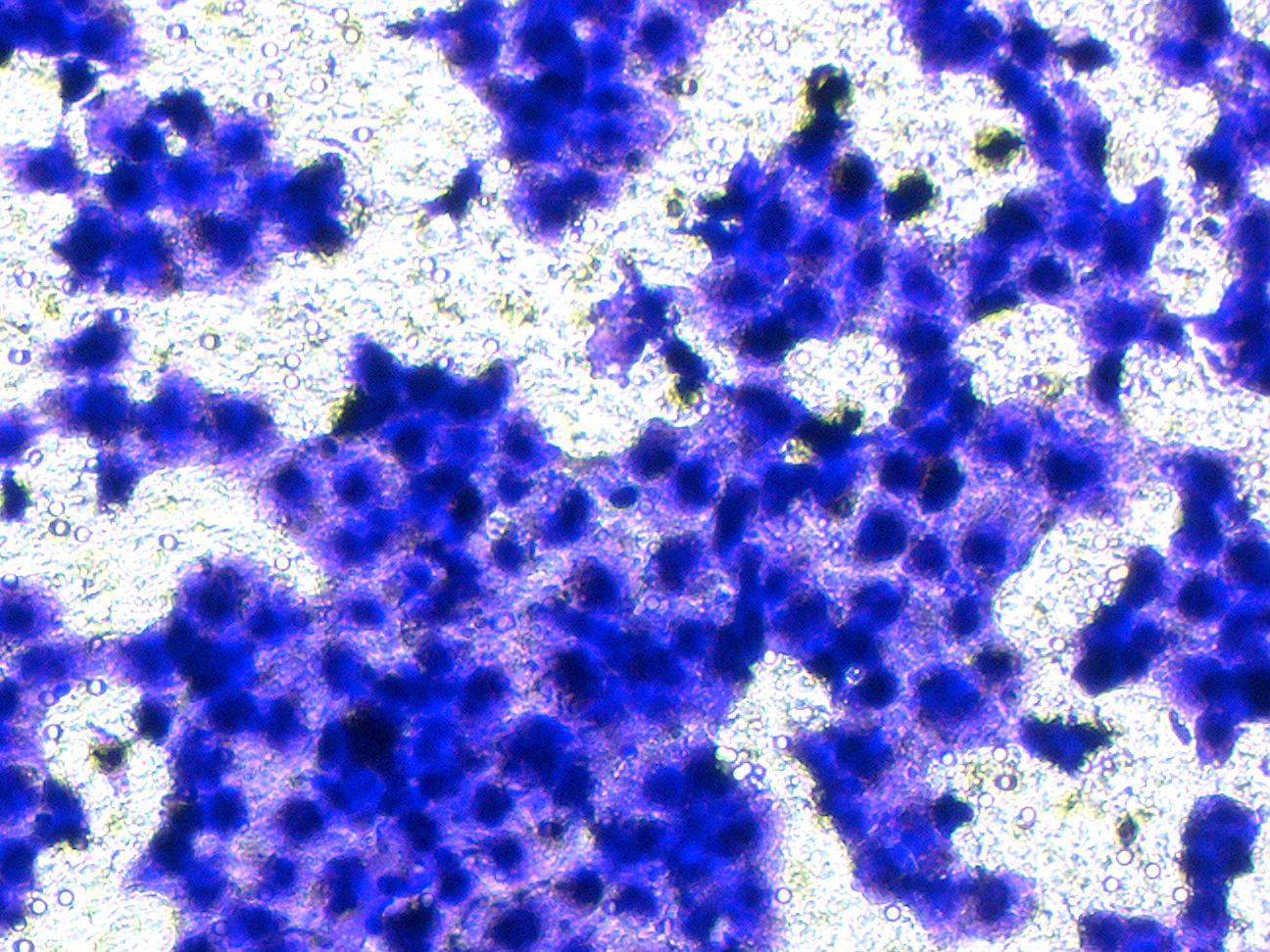

Supplement: Supplemental Material [file KBIE_A_2056692_SM9735.zip › supplementary/Fig3A_Control.jpg]

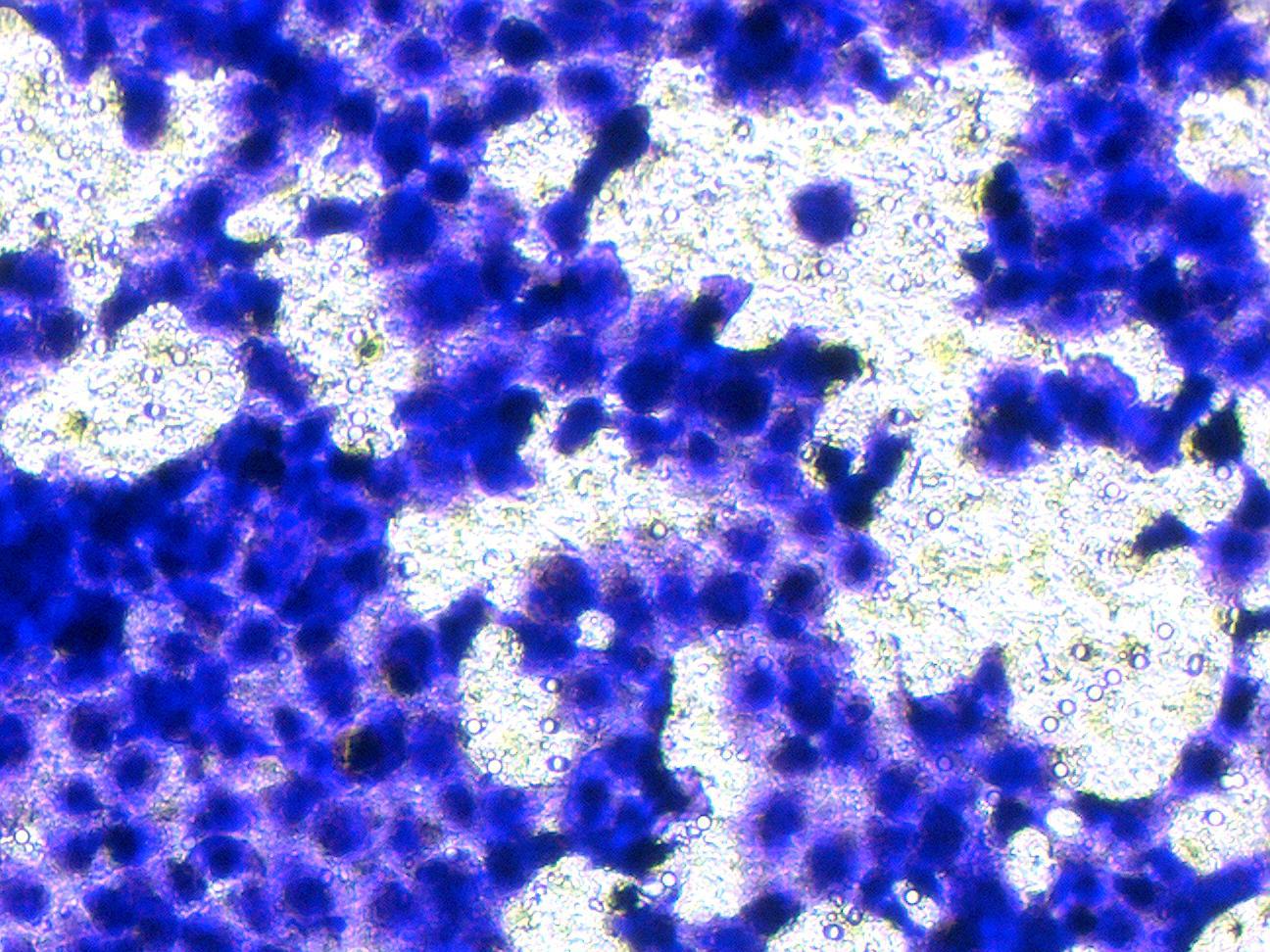

Supplement: Supplemental Material [file KBIE_A_2056692_SM9735.zip › supplementary/Fig3A_sh_NC.jpg]

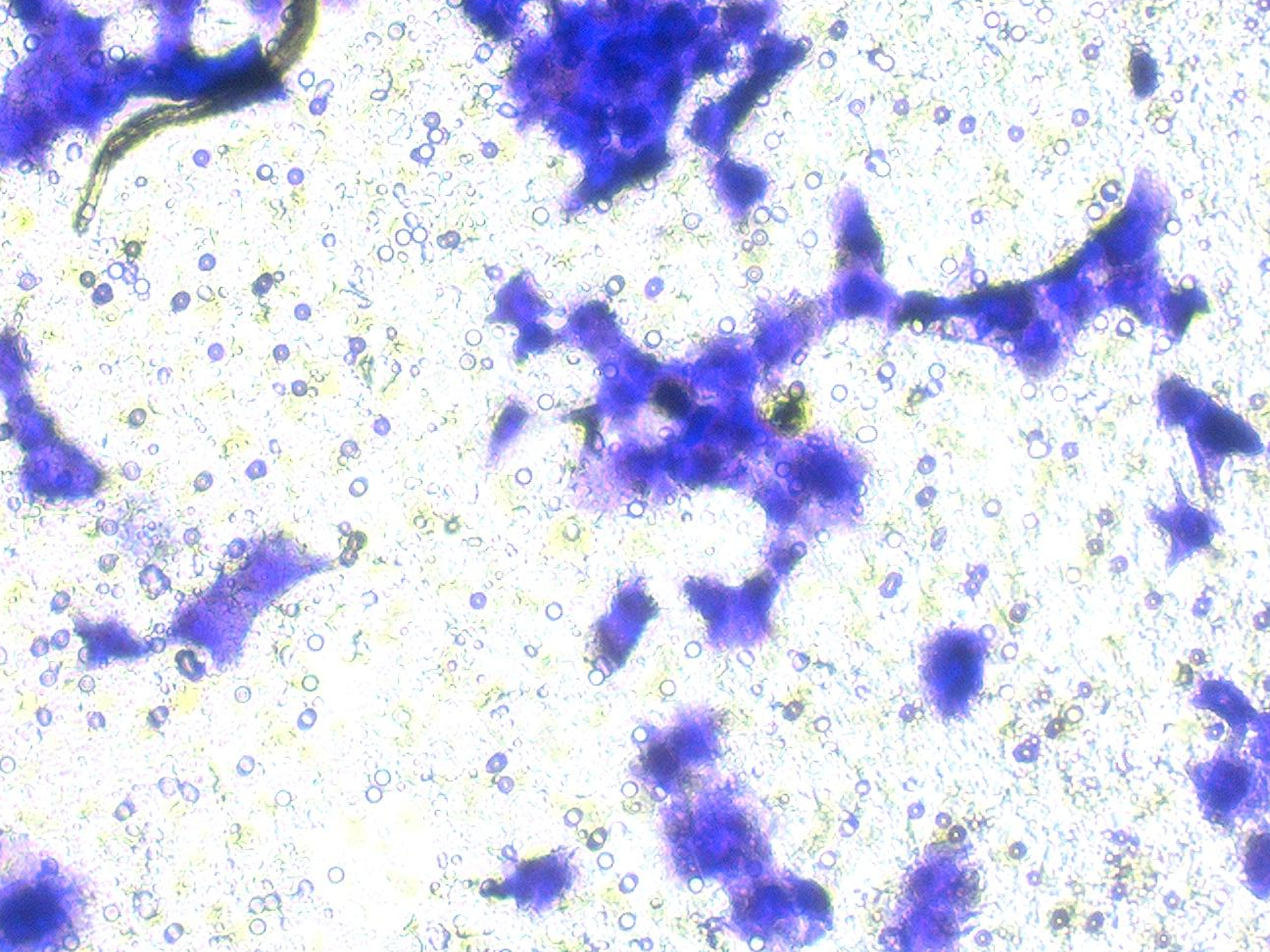

Supplement: Supplemental Material [file KBIE_A_2056692_SM9735.zip › supplementary/Fig3A_sh_VPS72_2.png]

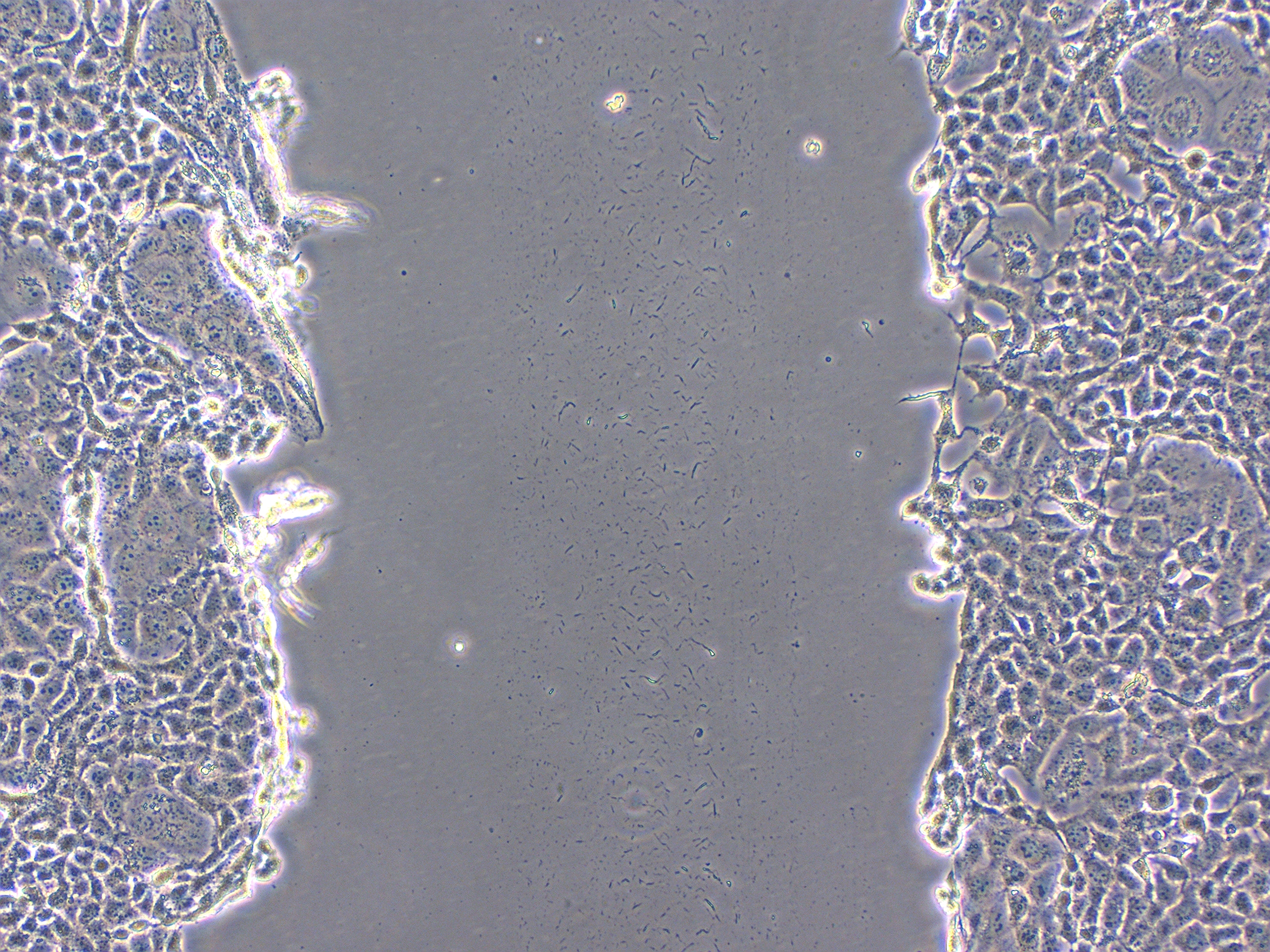

Supplement: Supplemental Material [file KBIE_A_2056692_SM9735.zip › supplementary/Fig3B_Control_0h.jpg]

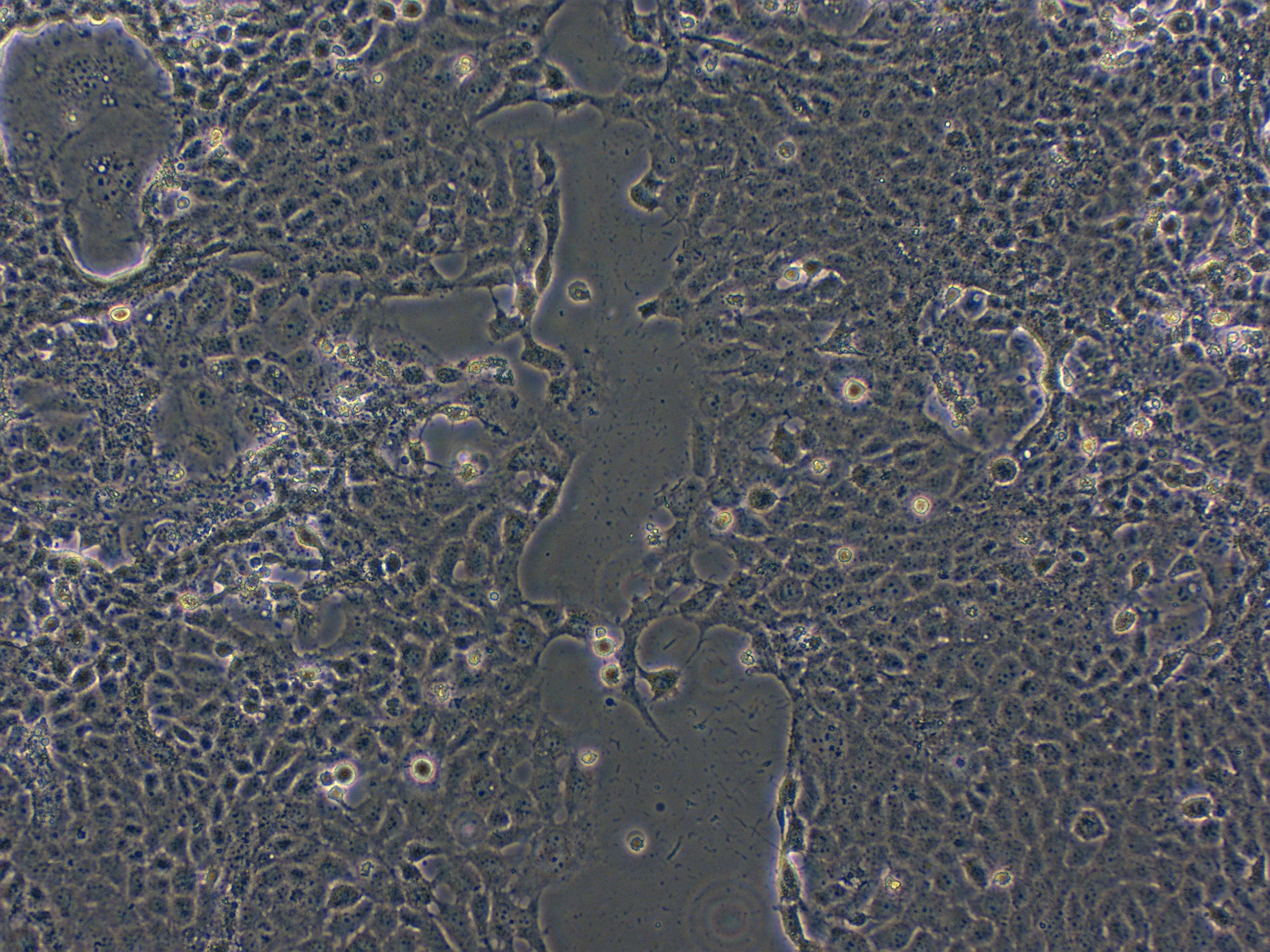

Supplement: Supplemental Material [file KBIE_A_2056692_SM9735.zip › supplementary/Fig3B_Control_24h.jpg]

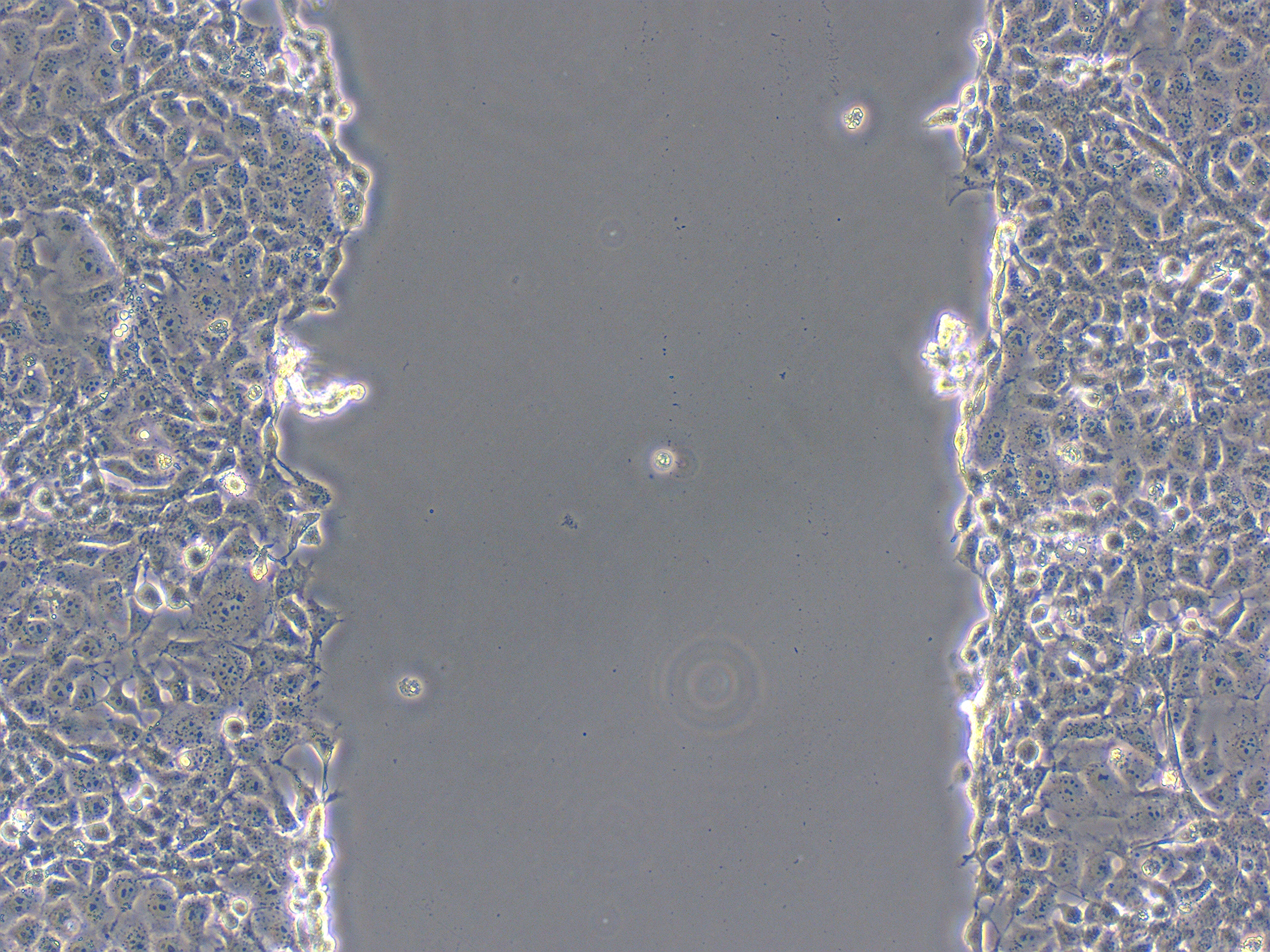

Supplement: Supplemental Material [file KBIE_A_2056692_SM9735.zip › supplementary/Fig3B_sh_NC_0h.jpg]

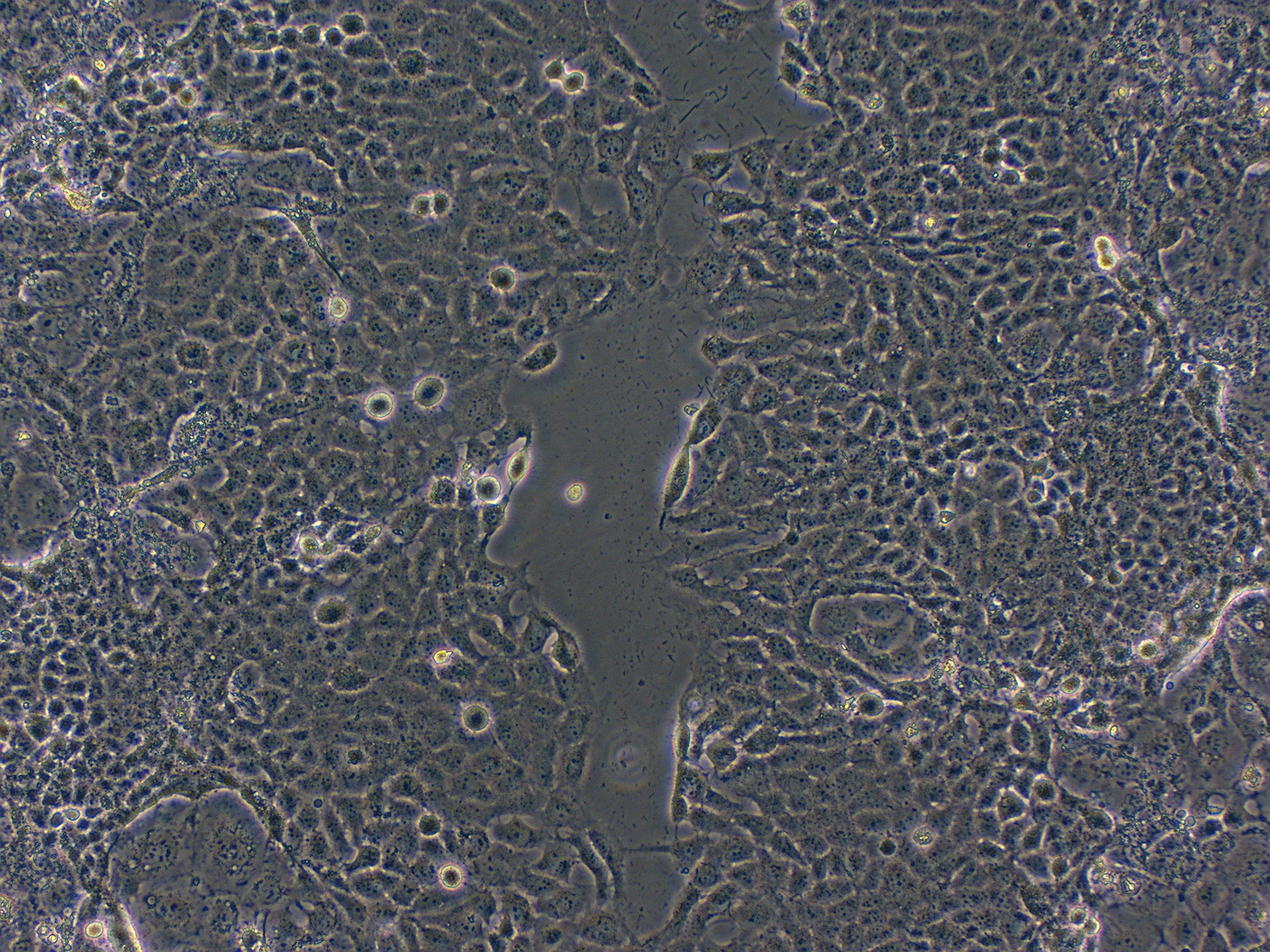

Supplement: Supplemental Material [file KBIE_A_2056692_SM9735.zip › supplementary/Fig3B_sh_NC_24h.jpg]

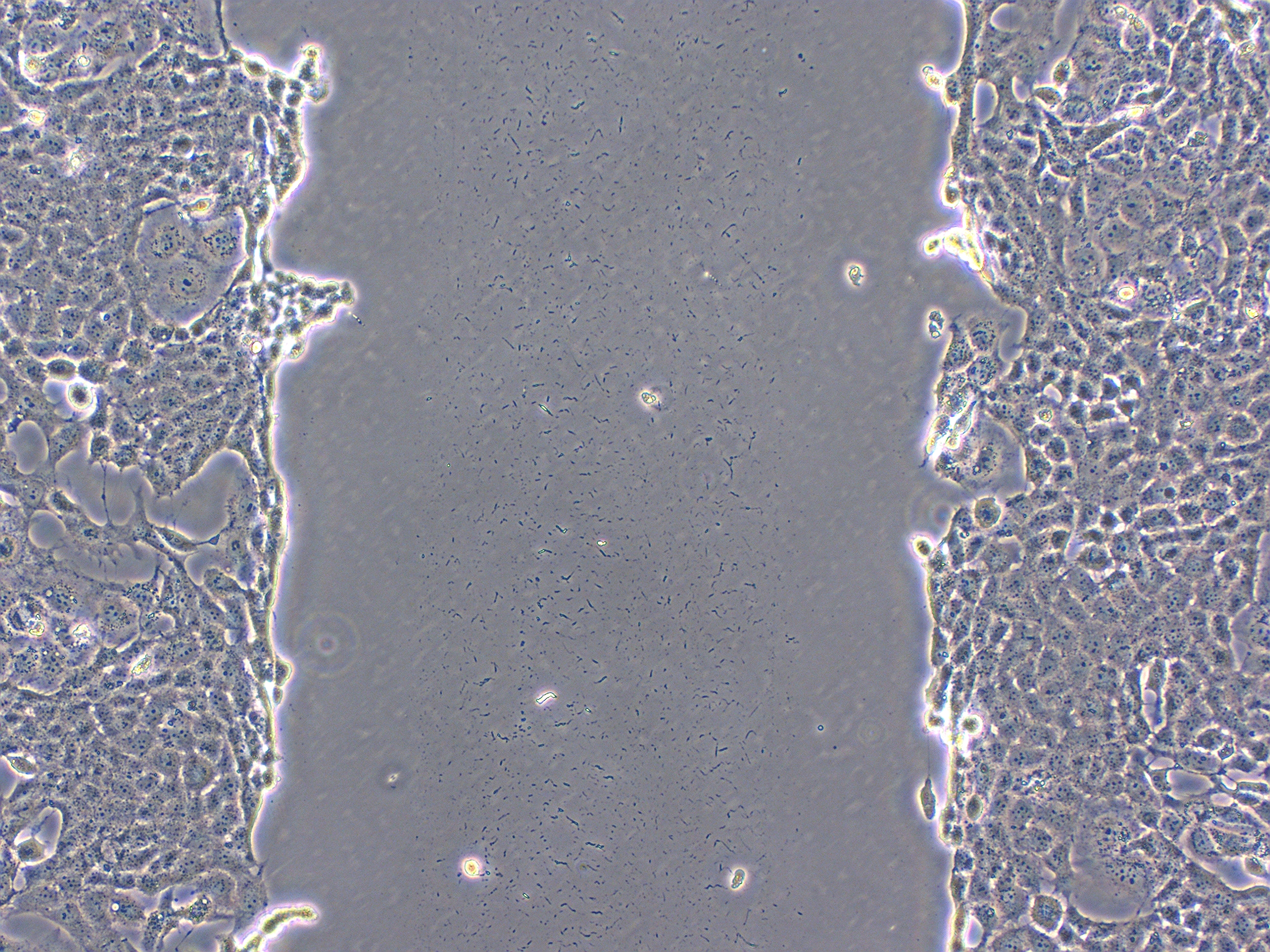

Supplement: Supplemental Material [file KBIE_A_2056692_SM9735.zip › supplementary/Fig3B_sh_VPS72_2_0h.jpg]

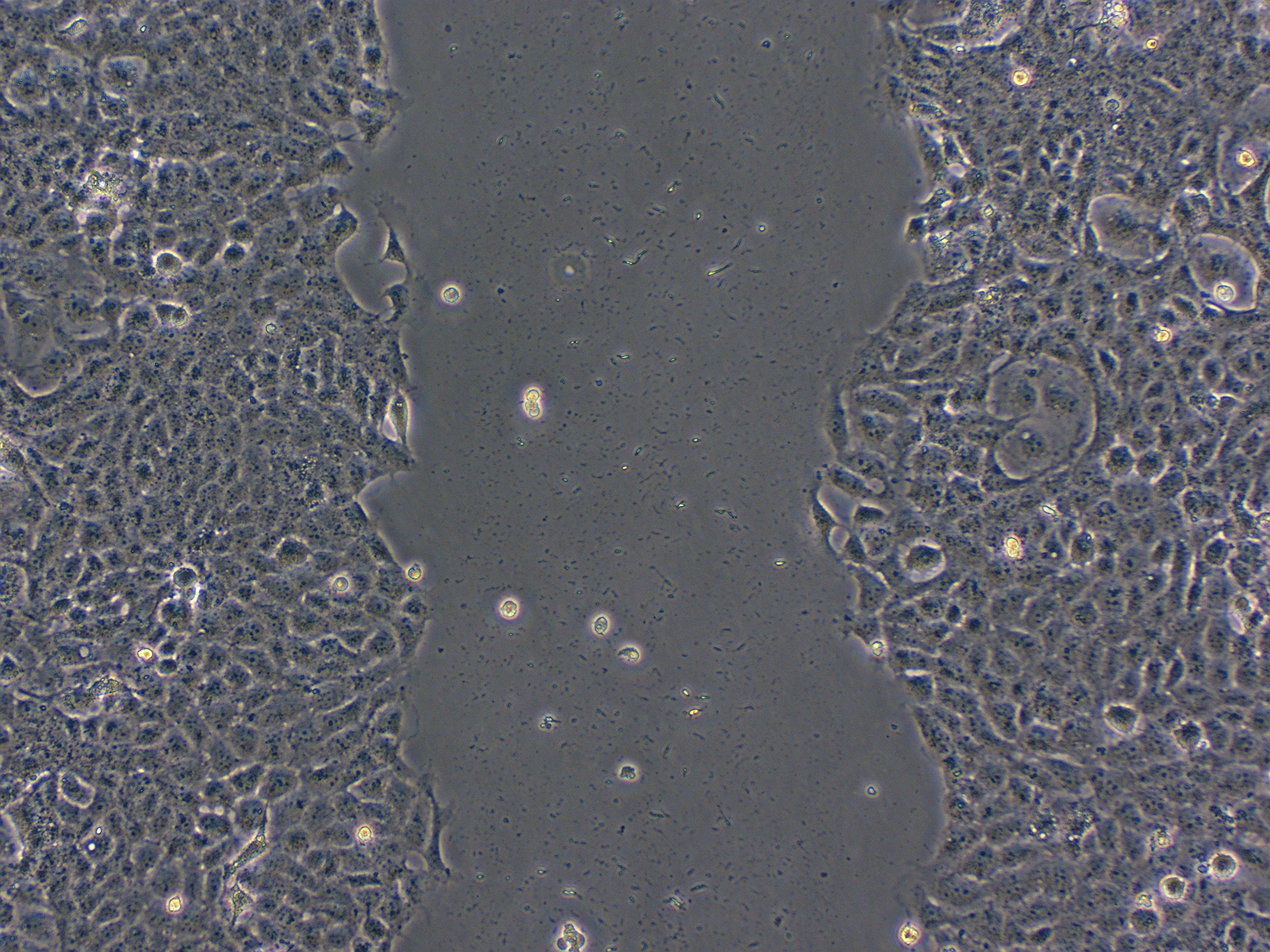

Supplement: Supplemental Material [file KBIE_A_2056692_SM9735.zip › supplementary/Fig3B_sh_VPS72_2_24h.jpg]

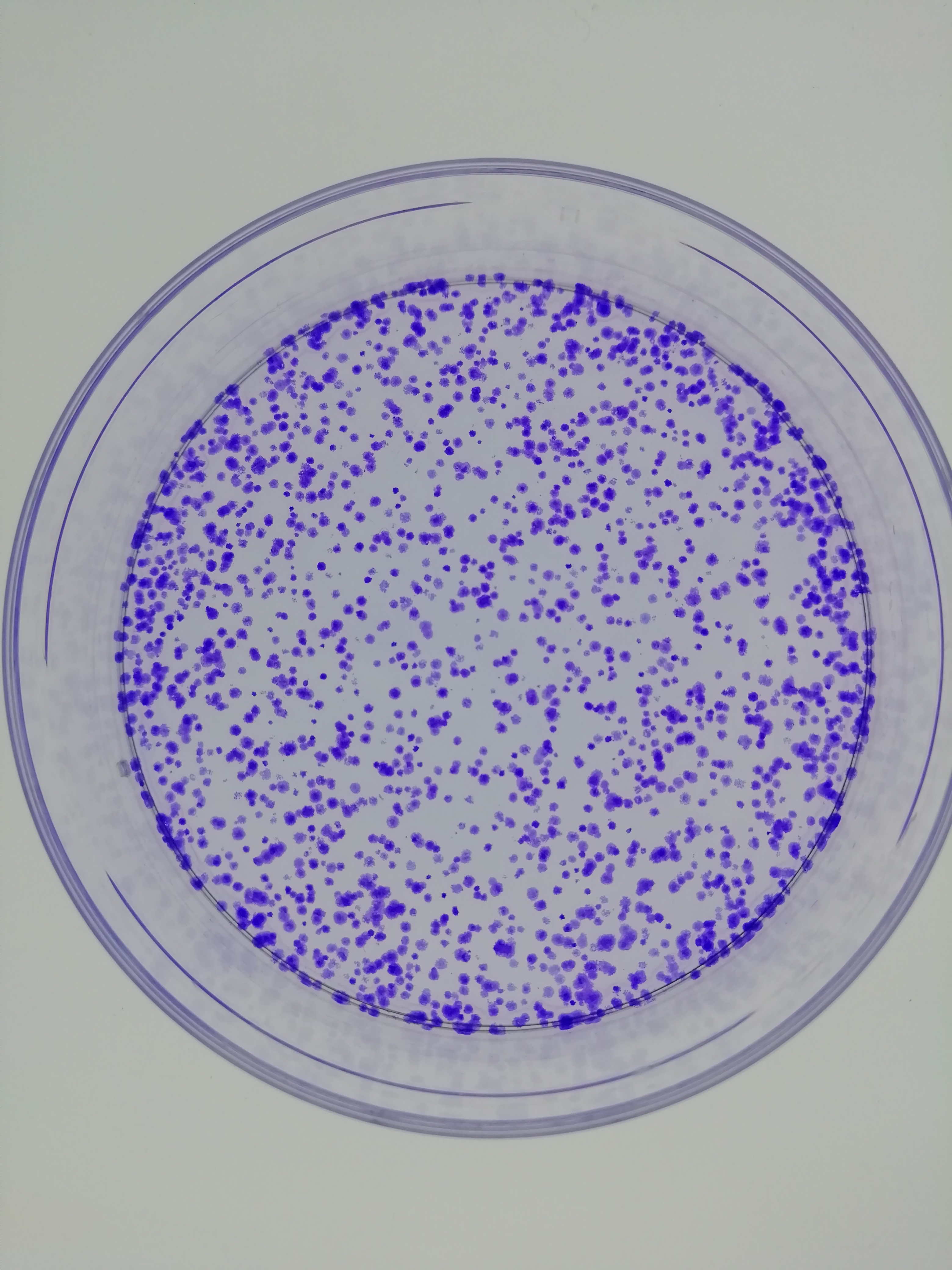

Supplement: Supplemental Material [file KBIE_A_2056692_SM9735.zip › supplementary/Fig5D_sh_NC.jpg]

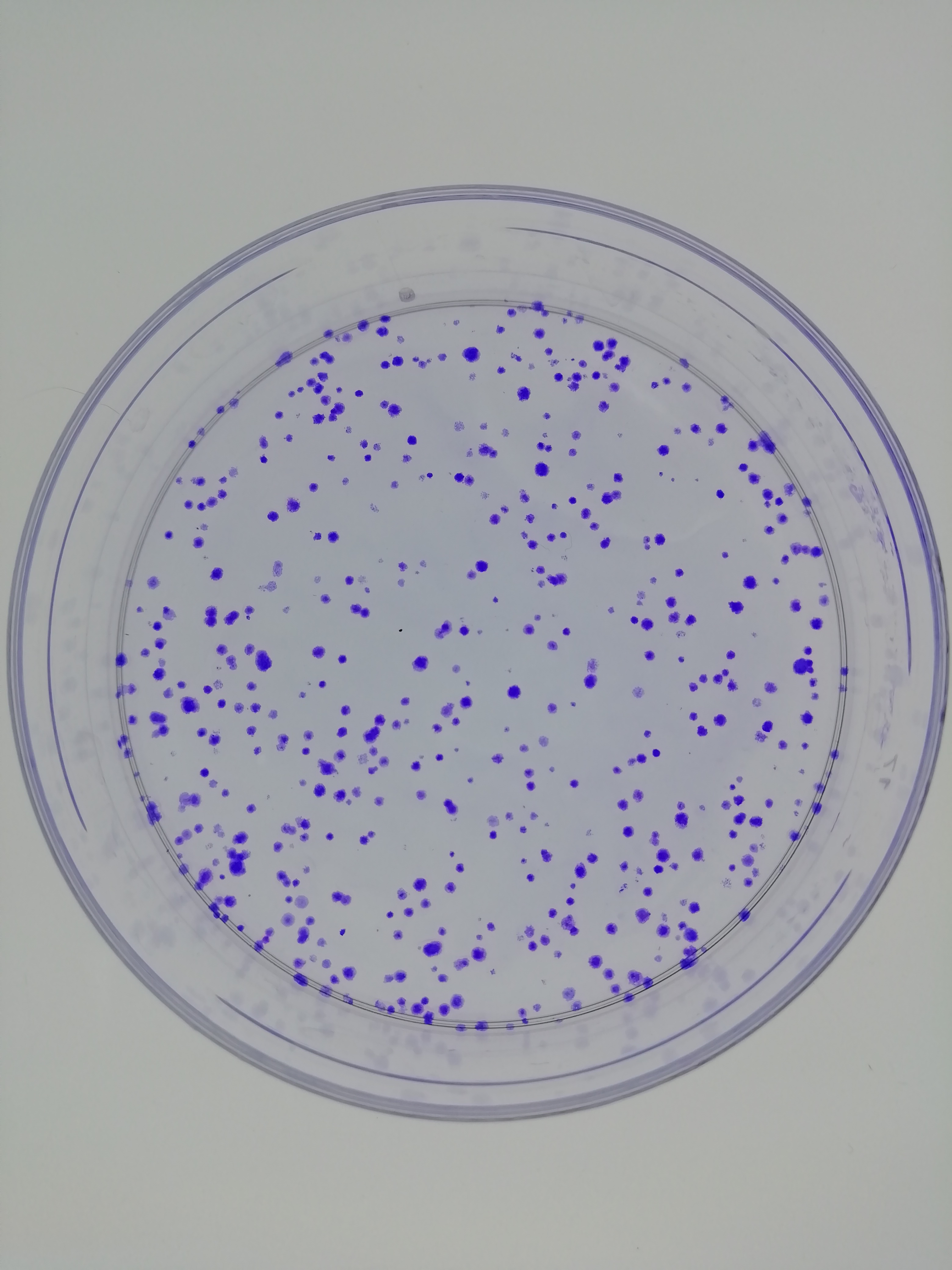

Supplement: Supplemental Material [file KBIE_A_2056692_SM9735.zip › supplementary/Fig5D_sh_VPS72_2.jpg]

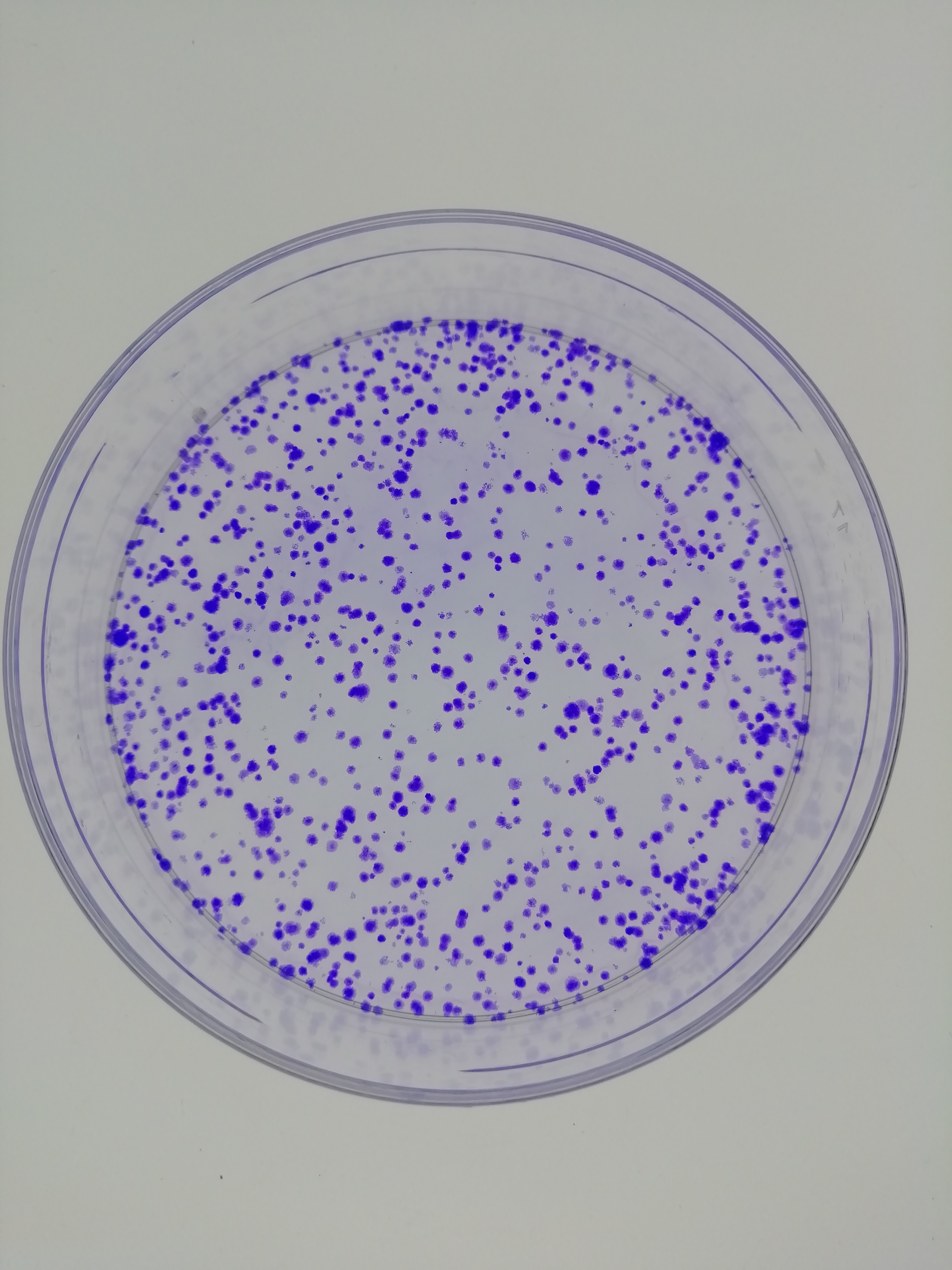

Supplement: Supplemental Material [file KBIE_A_2056692_SM9735.zip › supplementary/Fig5D_sh_VPS72_2_oe_KAT5.jpg]

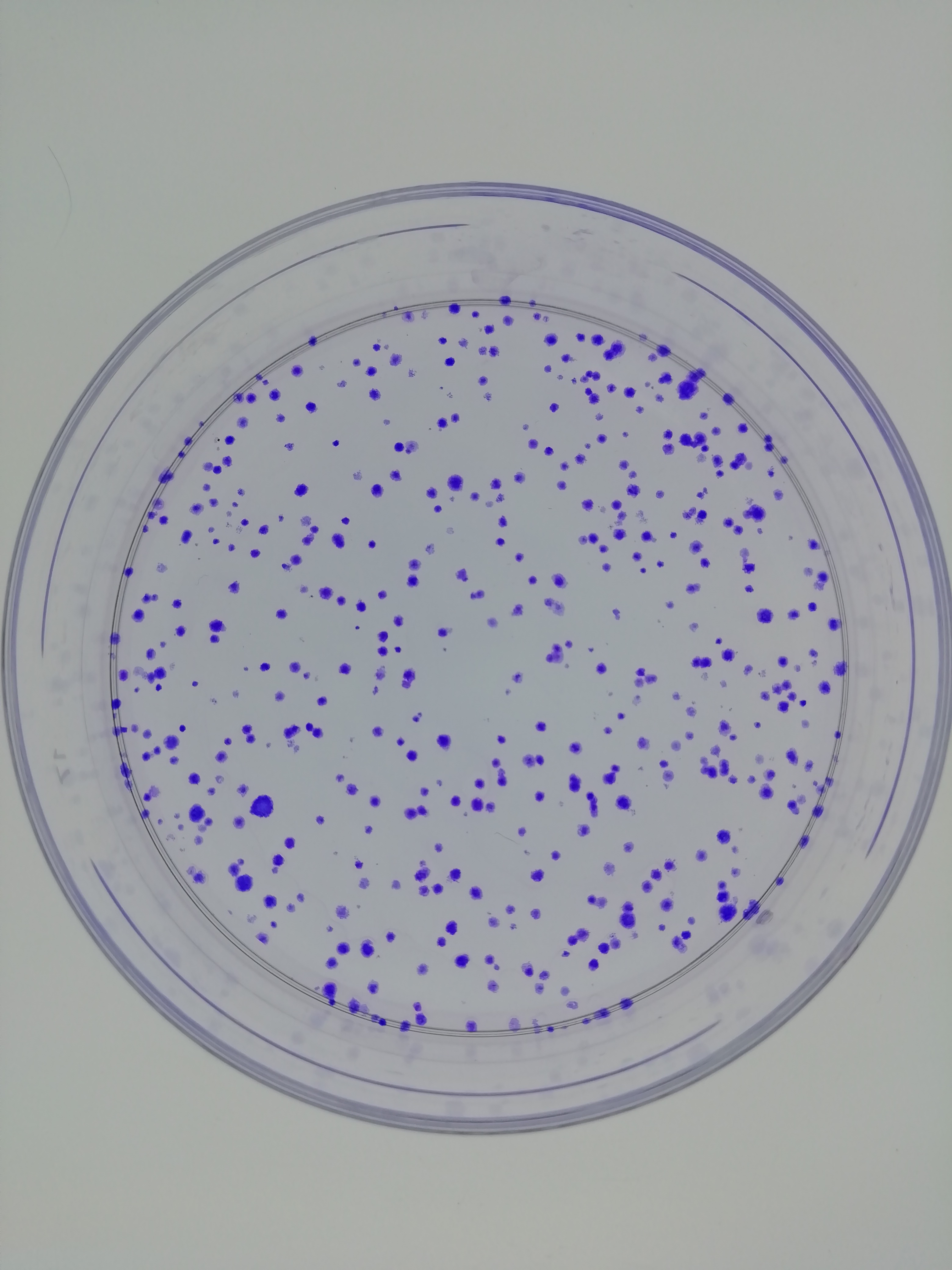

Supplement: Supplemental Material [file KBIE_A_2056692_SM9735.zip › supplementary/Fig5D_sh_VPS72_2_oe_NC.jpg]

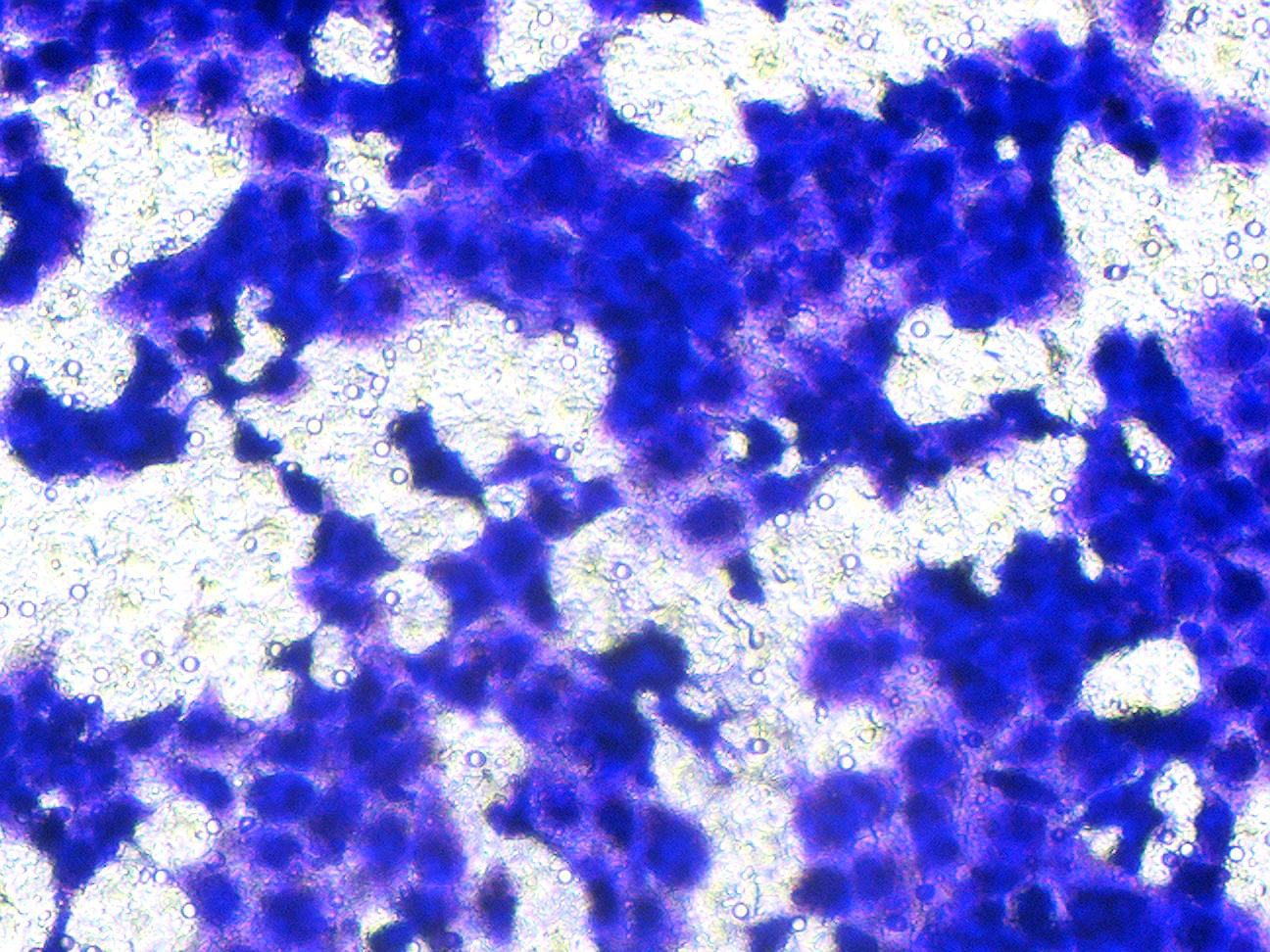

Supplement: Supplemental Material [file KBIE_A_2056692_SM9735.zip › supplementary/Fig6A_sh_NC.jpg]

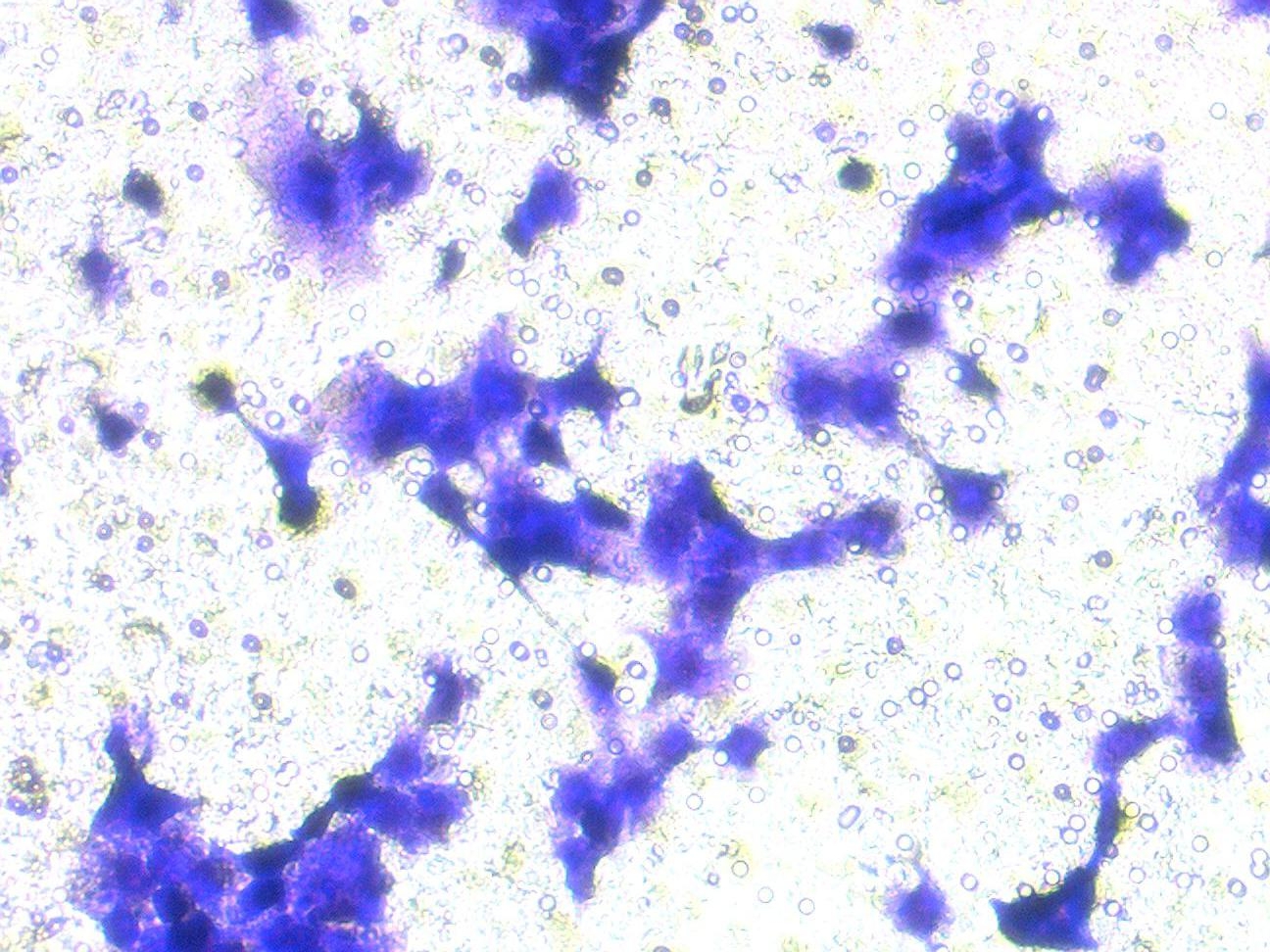

Supplement: Supplemental Material [file KBIE_A_2056692_SM9735.zip › supplementary/Fig6A_sh_VPS72_2.jpg]

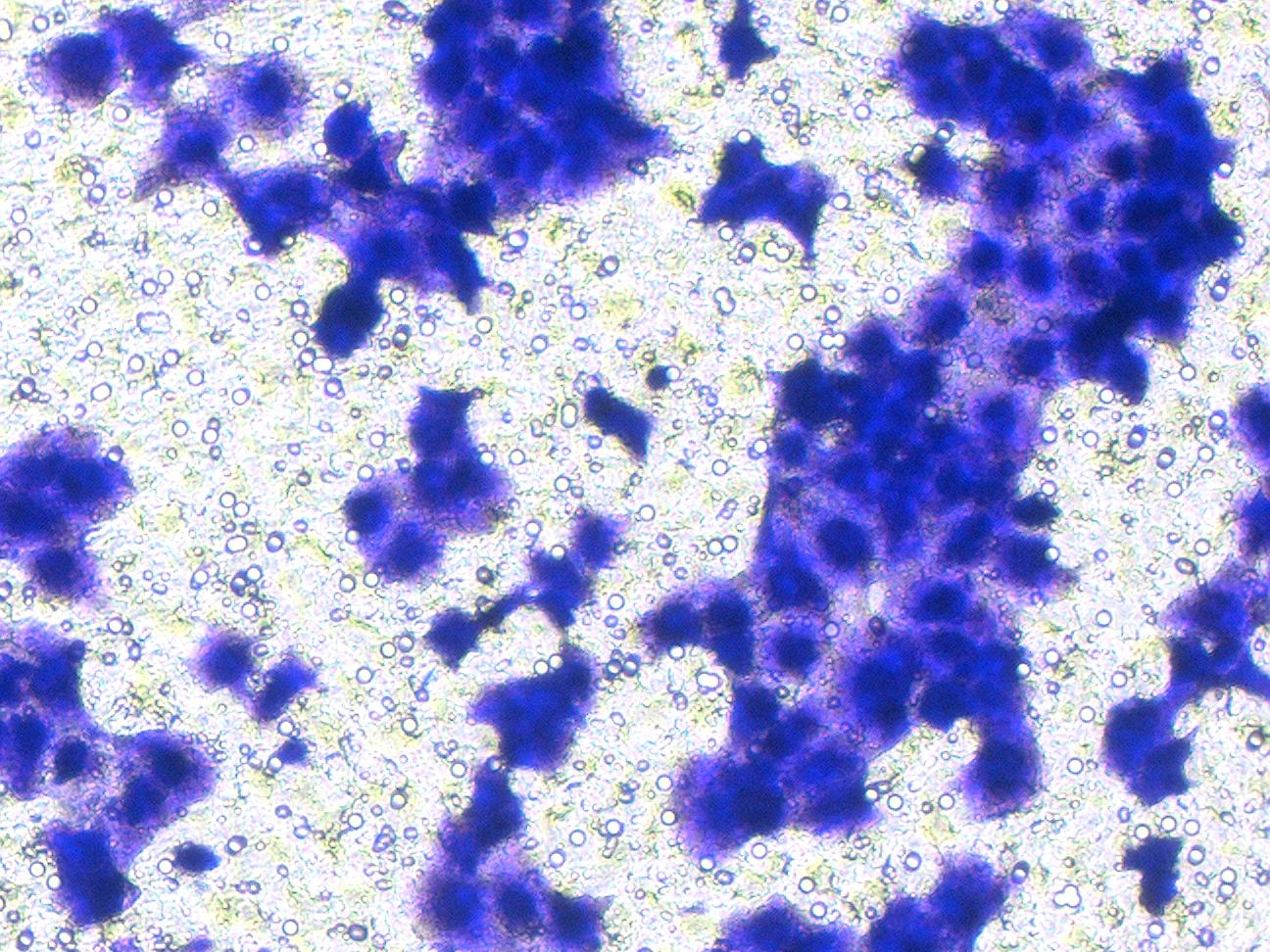

Supplement: Supplemental Material [file KBIE_A_2056692_SM9735.zip › supplementary/Fig6A_sh_VPS72_2_oe_KAT5.png]

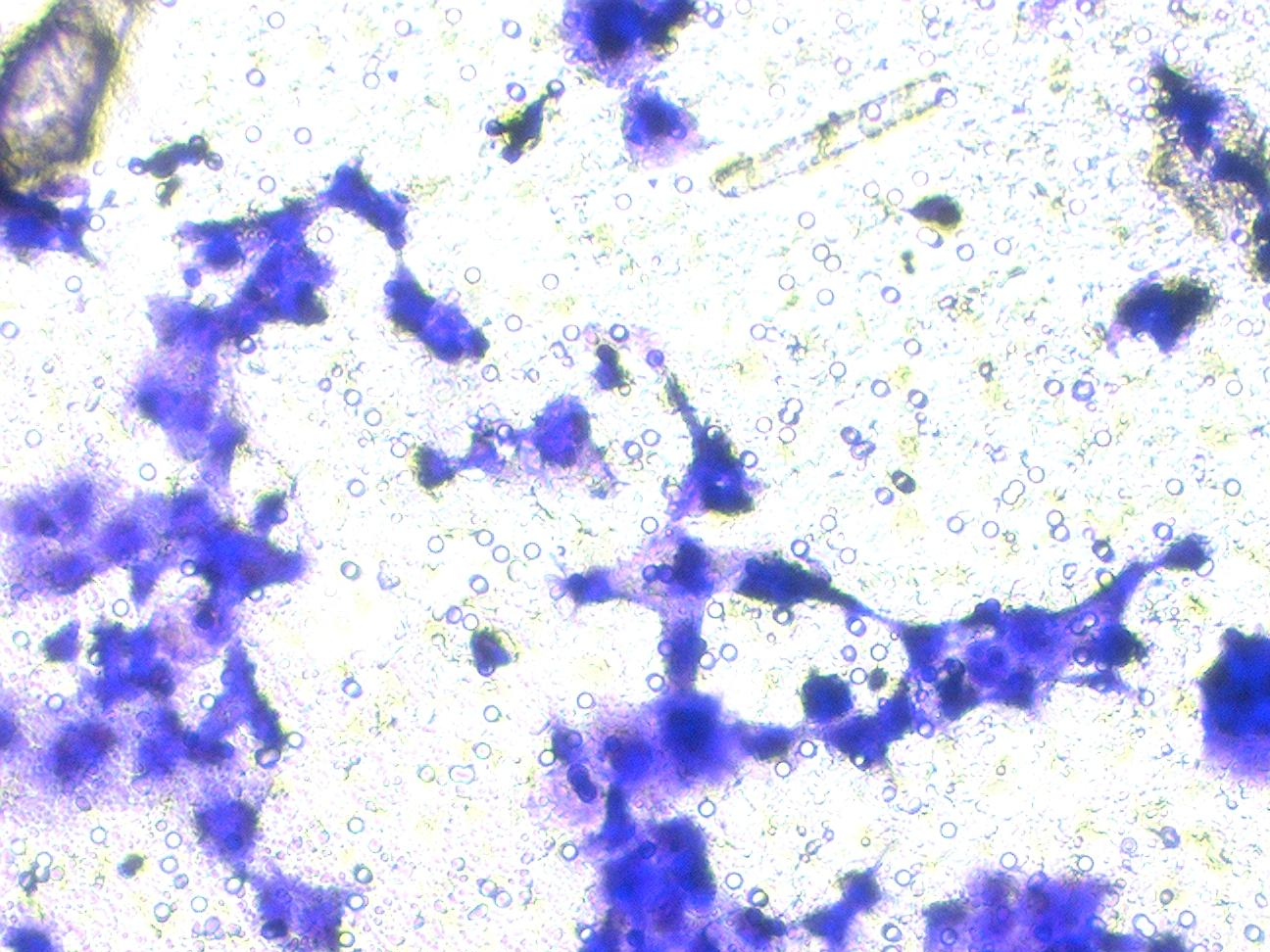

Supplement: Supplemental Material [file KBIE_A_2056692_SM9735.zip › supplementary/Fig6A_sh_VPS72_2_oe_NC.png]

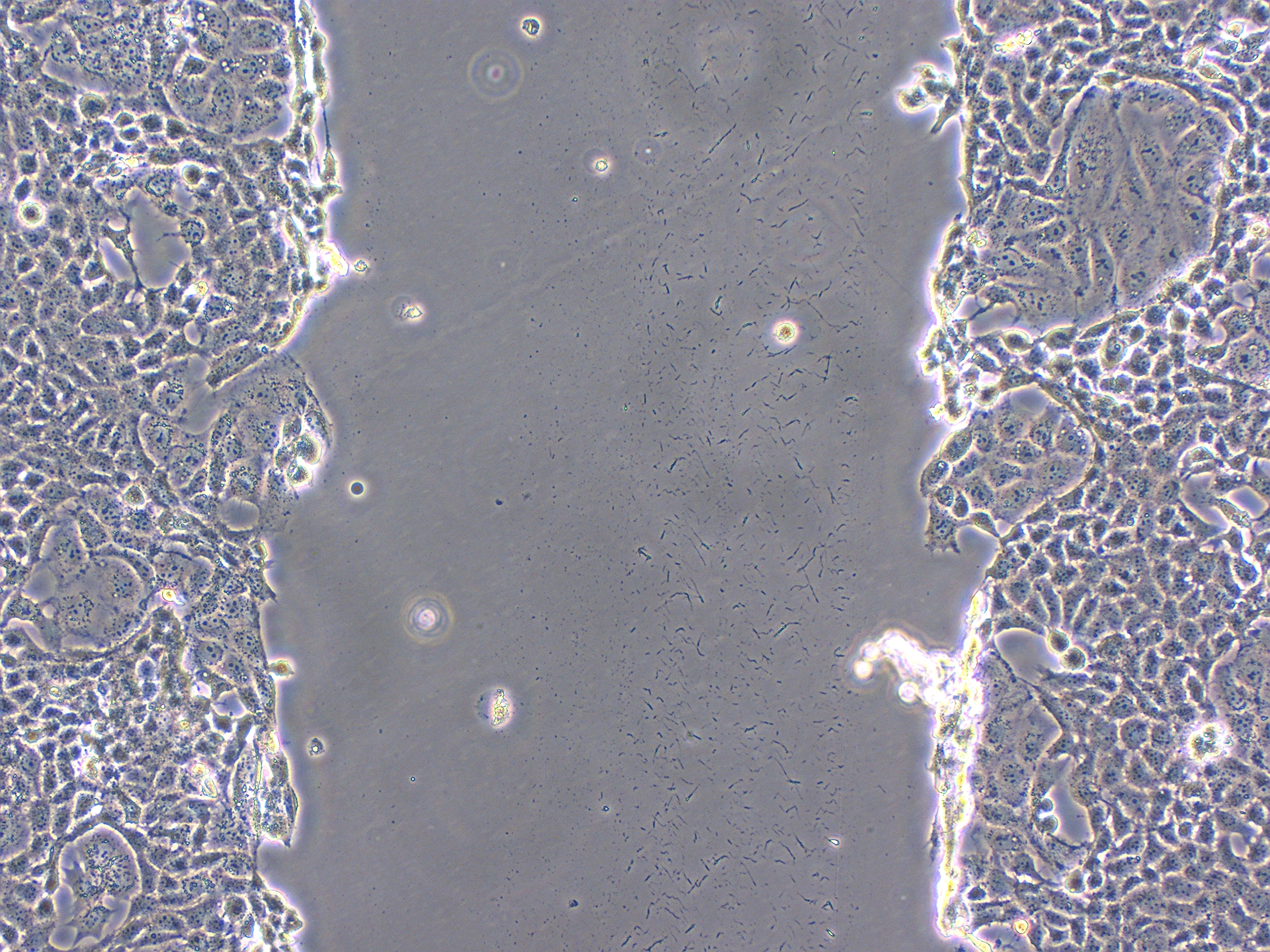

Supplement: Supplemental Material [file KBIE_A_2056692_SM9735.zip › supplementary/Fig6B_sh_NC_0h.jpg]

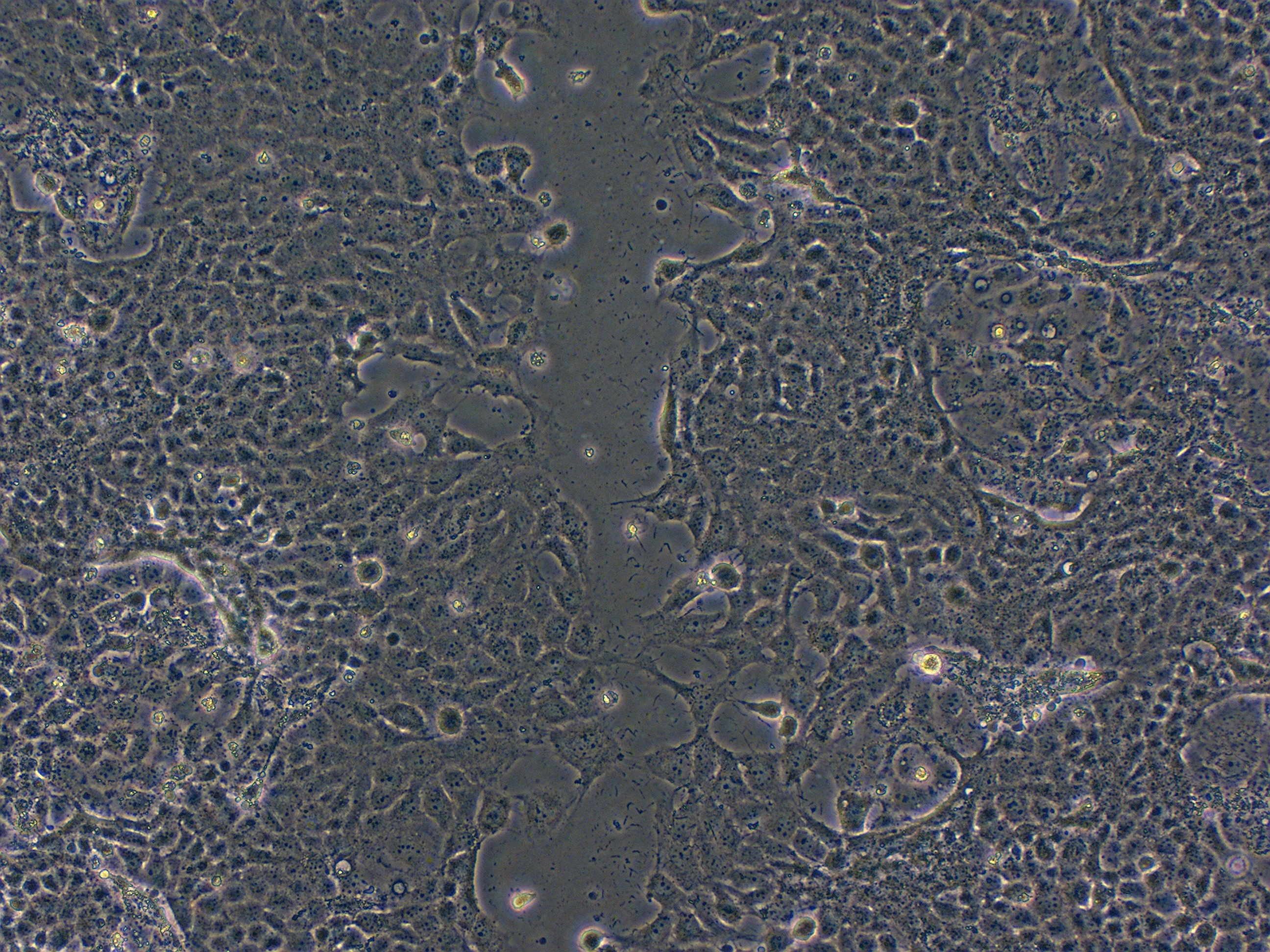

Supplement: Supplemental Material [file KBIE_A_2056692_SM9735.zip › supplementary/Fig6B_sh_NC_24h.jpg]

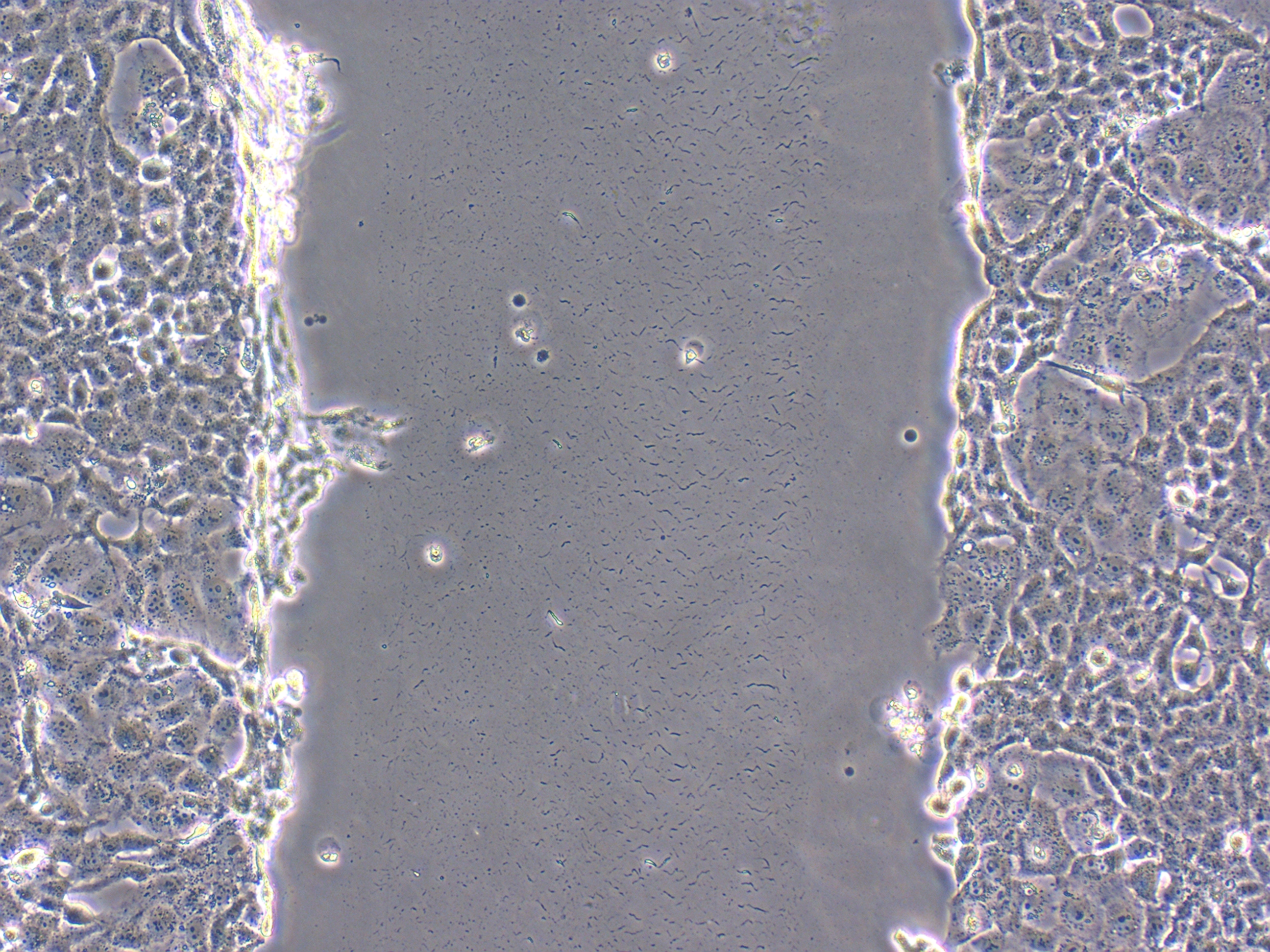

Supplement: Supplemental Material [file KBIE_A_2056692_SM9735.zip › supplementary/Fig6B_sh_VPS72_2_0h.jpg]

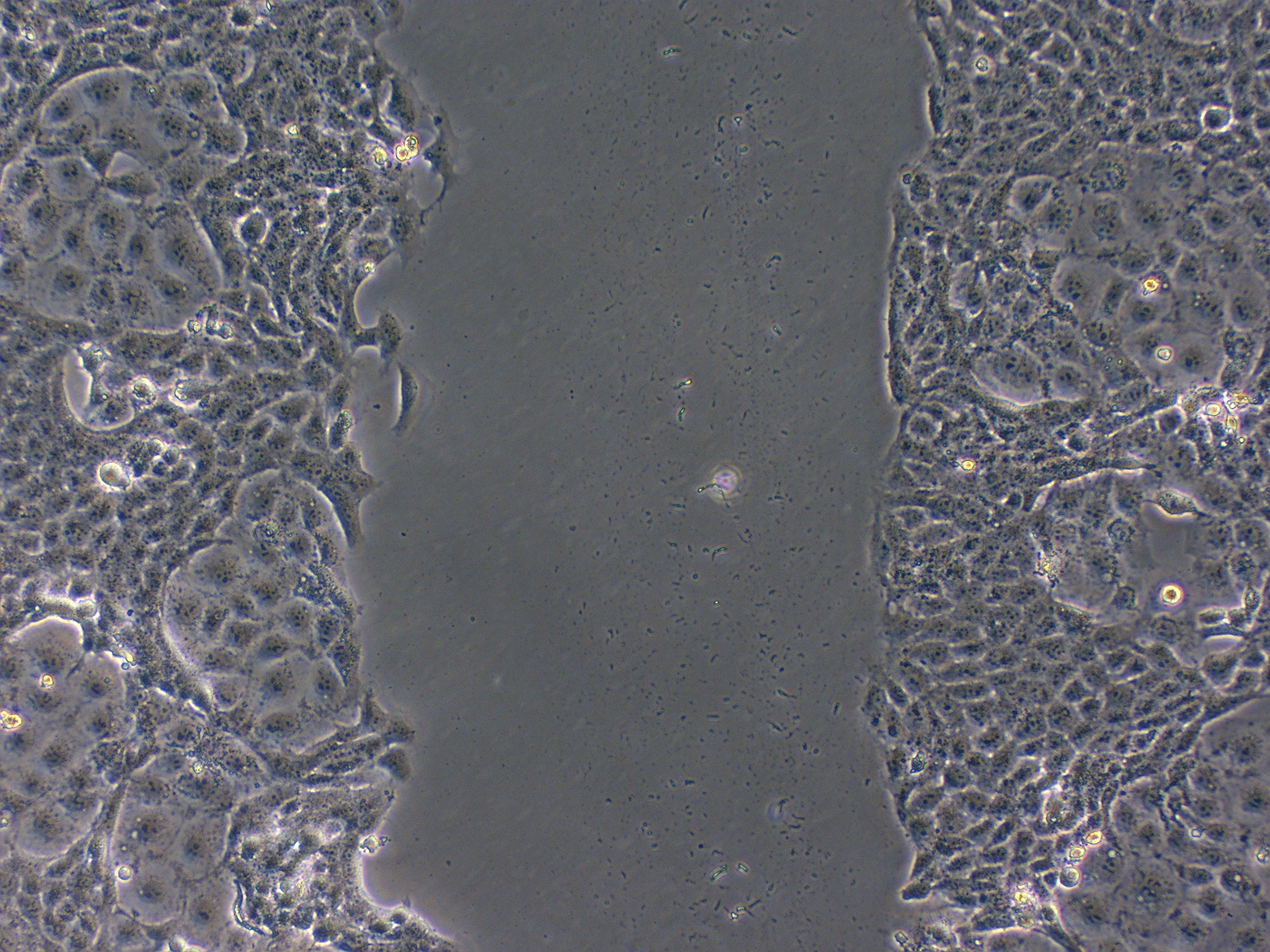

Supplement: Supplemental Material [file KBIE_A_2056692_SM9735.zip › supplementary/Fig6B_sh_VPS72_2_24h.jpg]

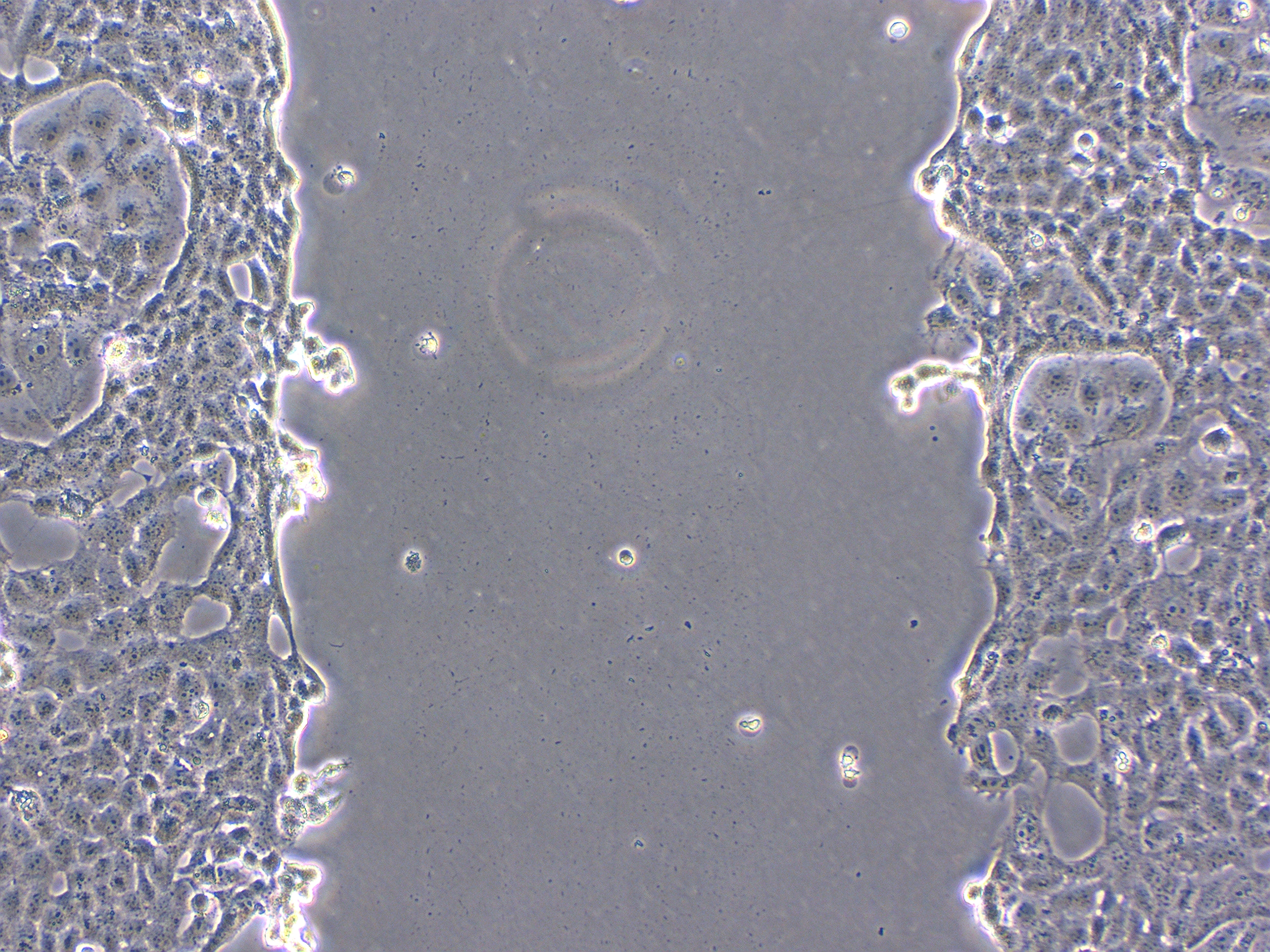

Supplement: Supplemental Material [file KBIE_A_2056692_SM9735.zip › supplementary/Fig6B_sh_VPS72_2_oe_KAT5_0h.jpg]

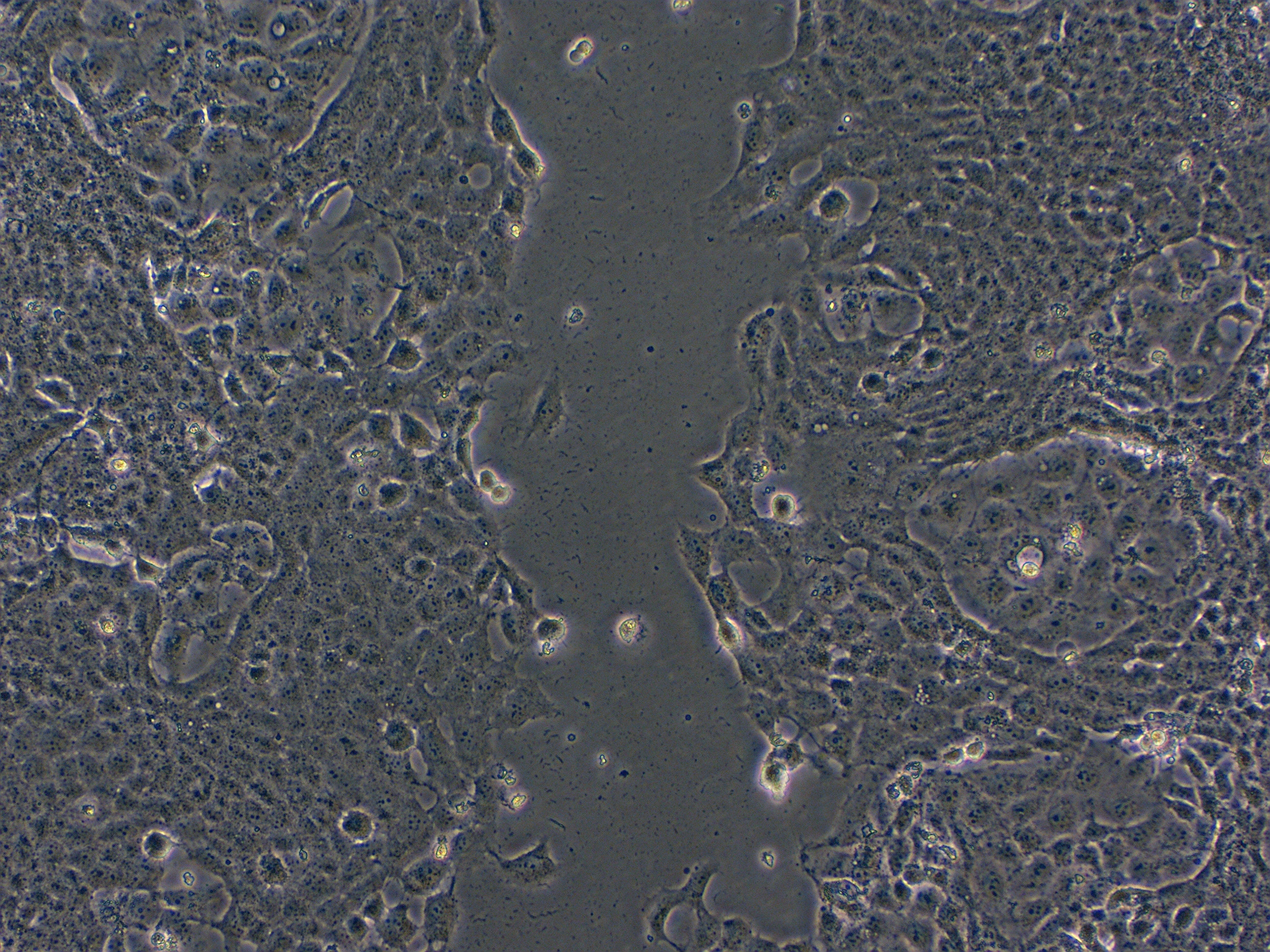

Supplement: Supplemental Material [file KBIE_A_2056692_SM9735.zip › supplementary/Fig6B_sh_VPS72_2_oe_KAT5_24h.jpg]

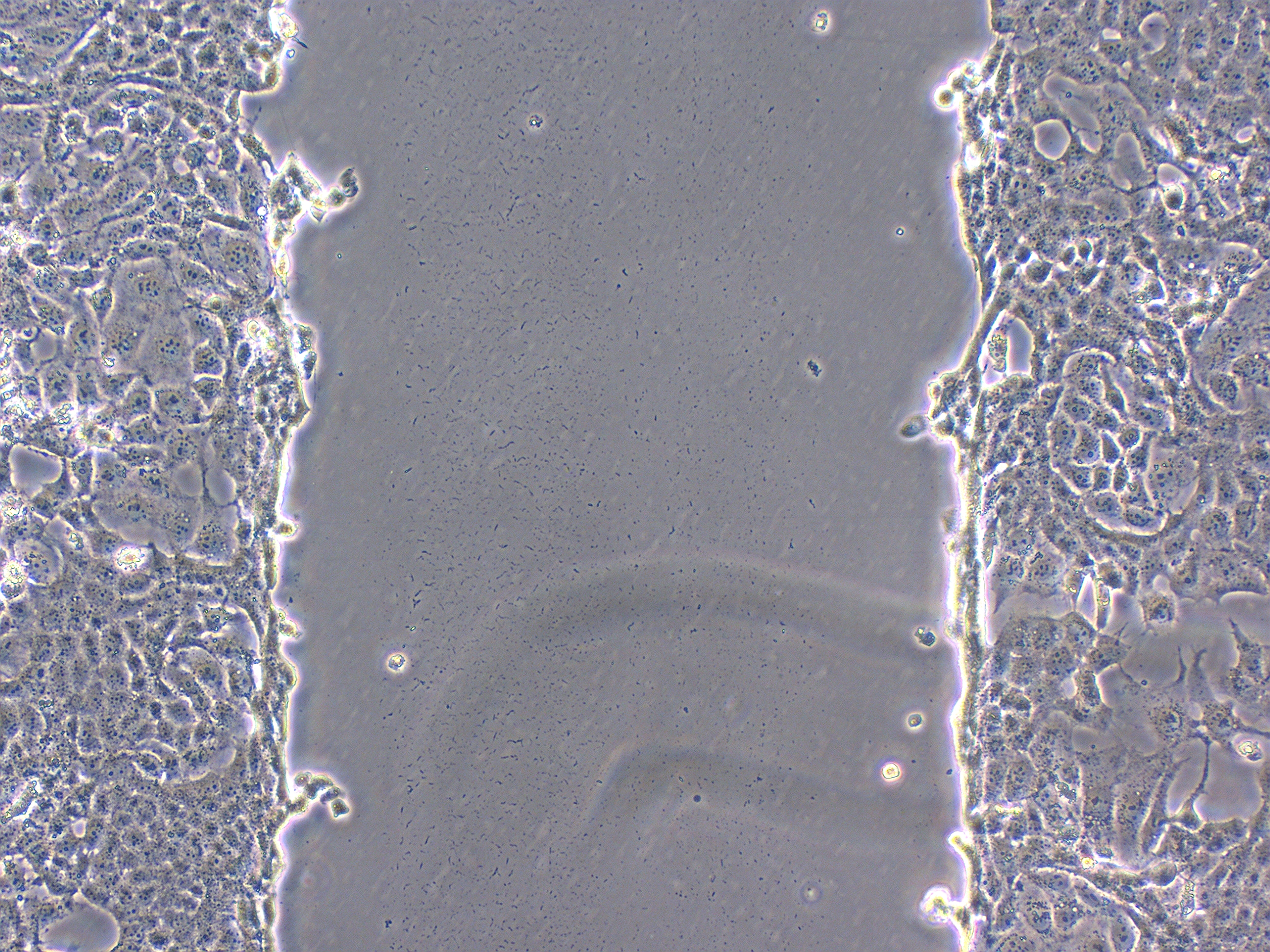

Supplement: Supplemental Material [file KBIE_A_2056692_SM9735.zip › supplementary/Fig6B_sh_VPS72_2_oe_NC_0h.jpg]

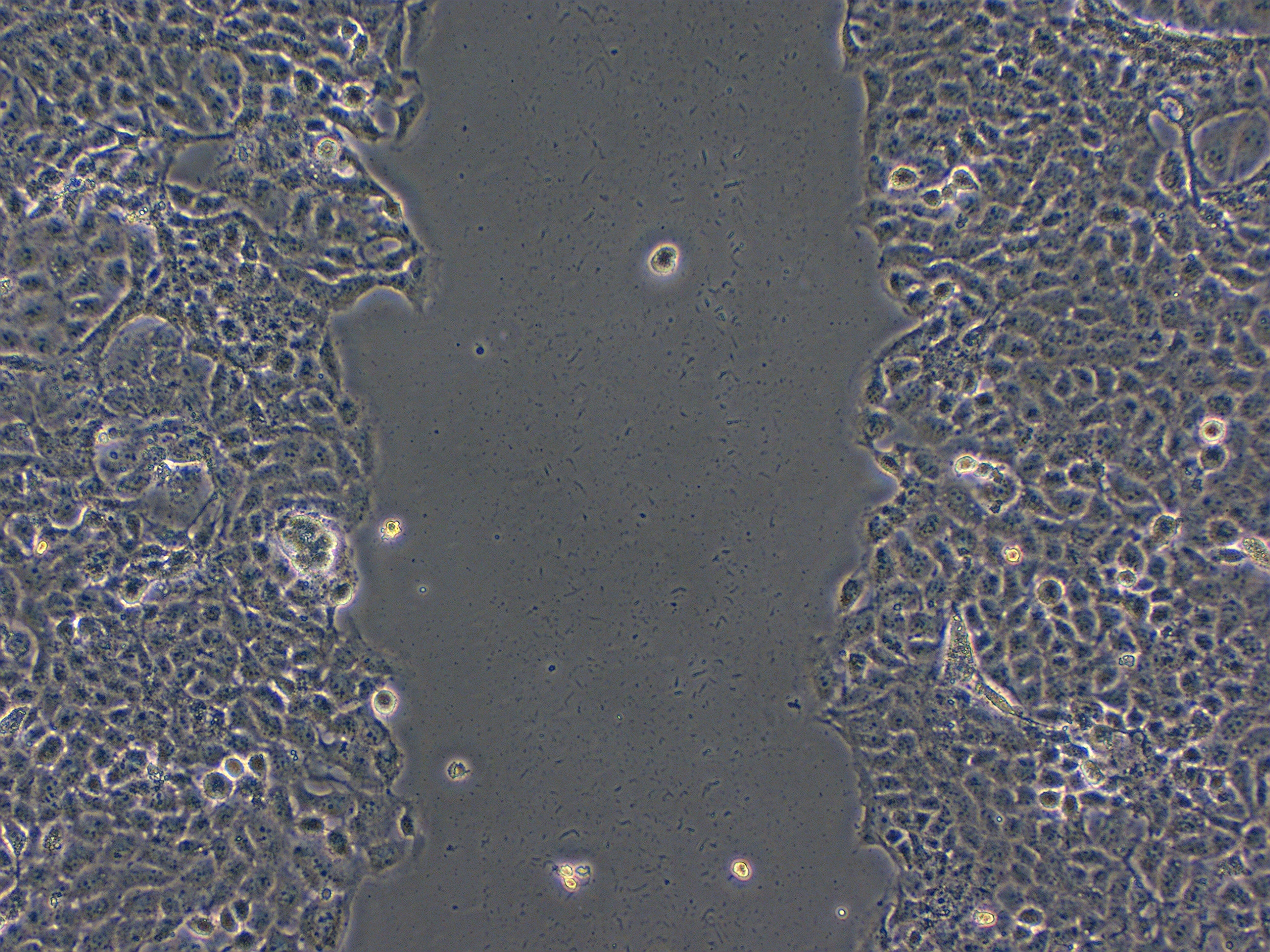

Supplement: Supplemental Material [file KBIE_A_2056692_SM9735.zip › supplementary/Fig6B_sh_VPS72_2_oe_NC_24h.jpg]

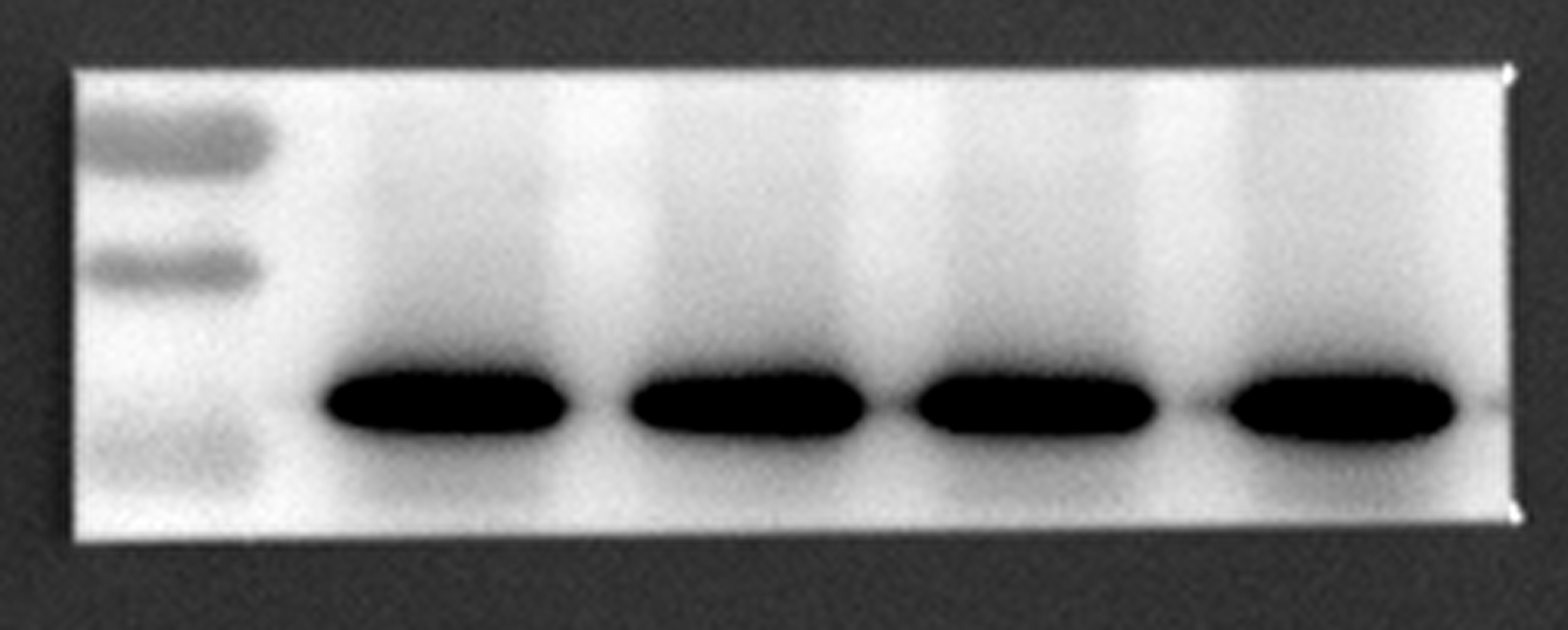

Supplement: Supplemental Material [file KBIE_A_2056692_SM9735.zip › supplementary/Figure1F_GAPDH.tif]

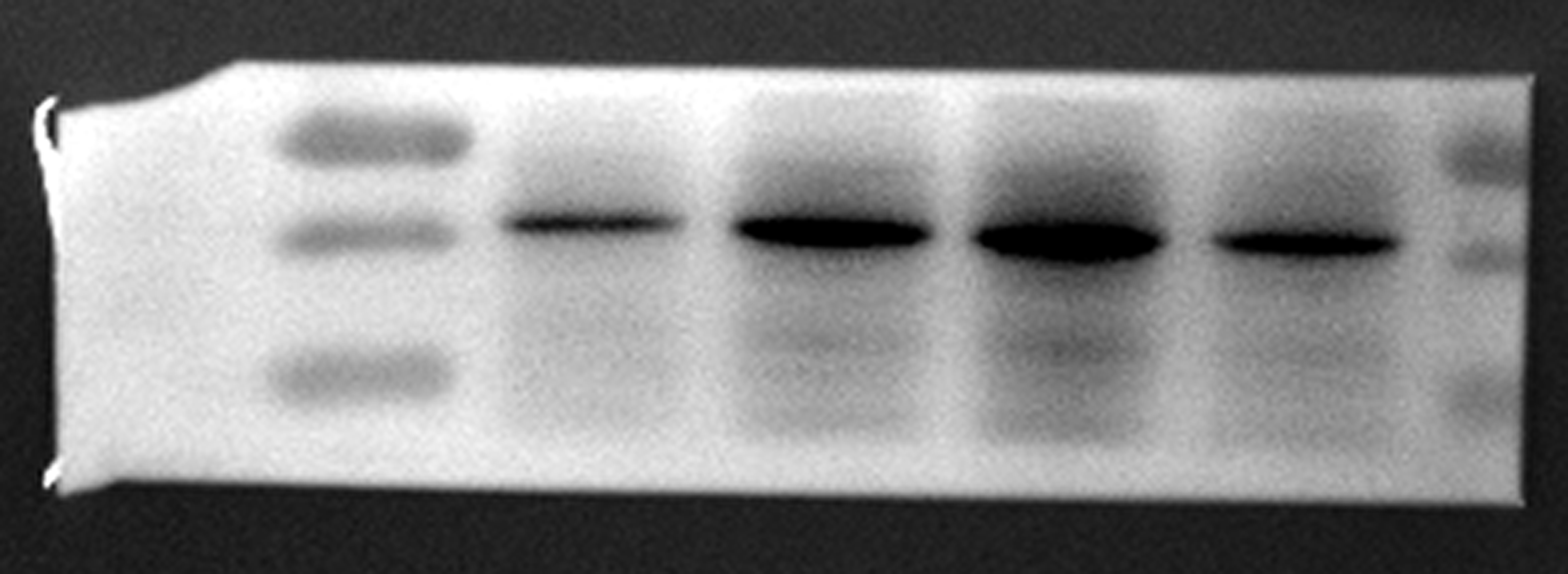

Supplement: Supplemental Material [file KBIE_A_2056692_SM9735.zip › supplementary/Figure1F_VPS72.tif]

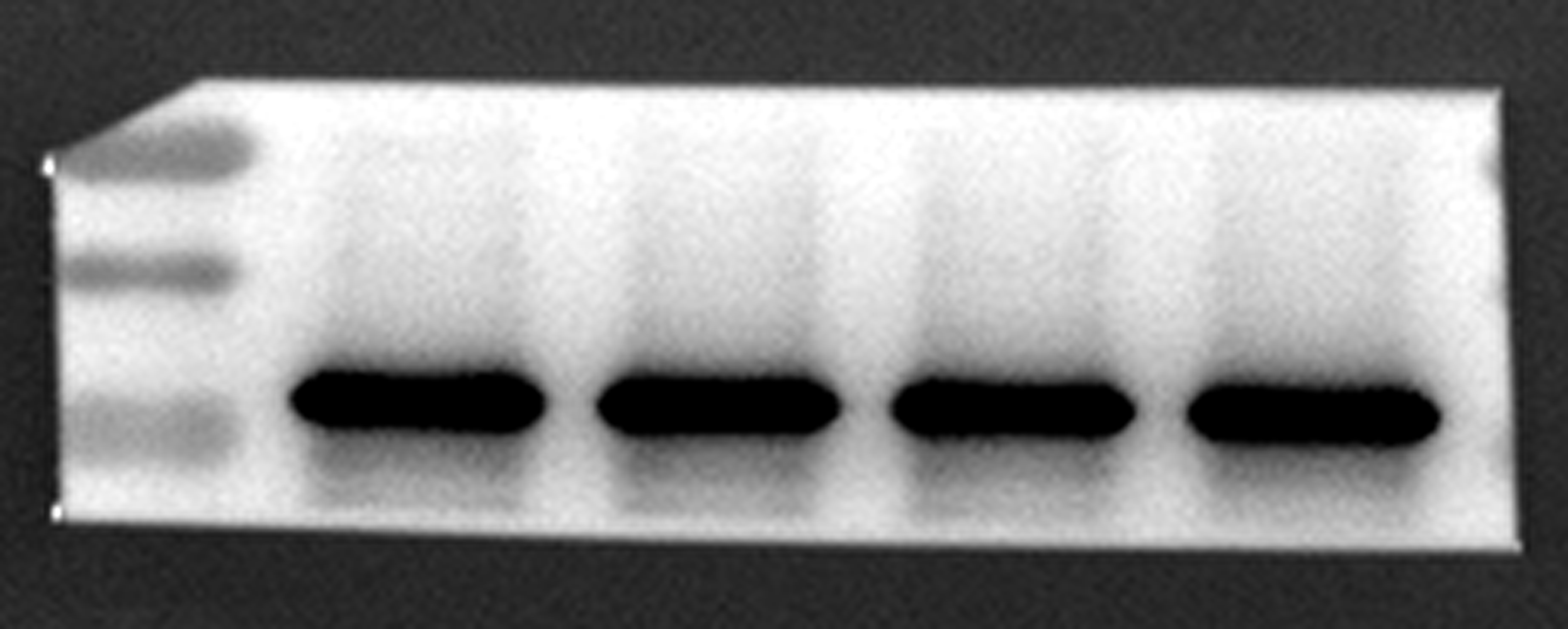

Supplement: Supplemental Material [file KBIE_A_2056692_SM9735.zip › supplementary/Figure2A_GAPDH.tif]

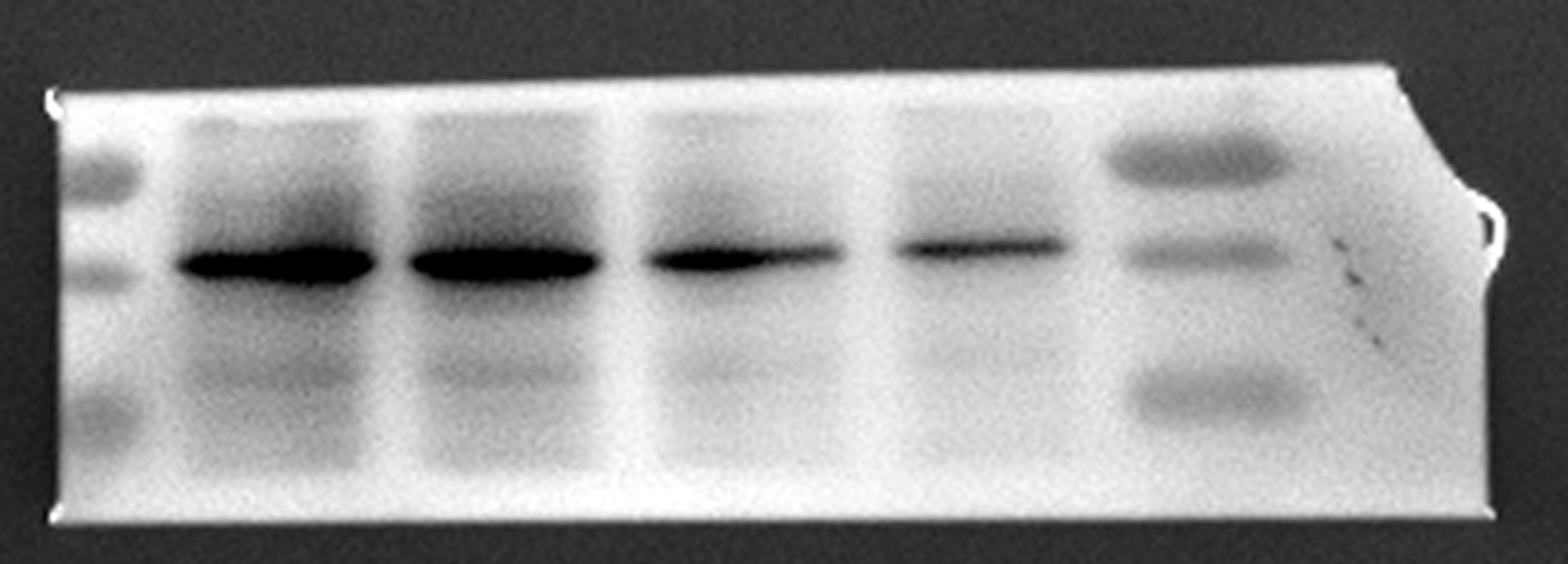

Supplement: Supplemental Material [file KBIE_A_2056692_SM9735.zip › supplementary/Figure2A_VPS72.tif]

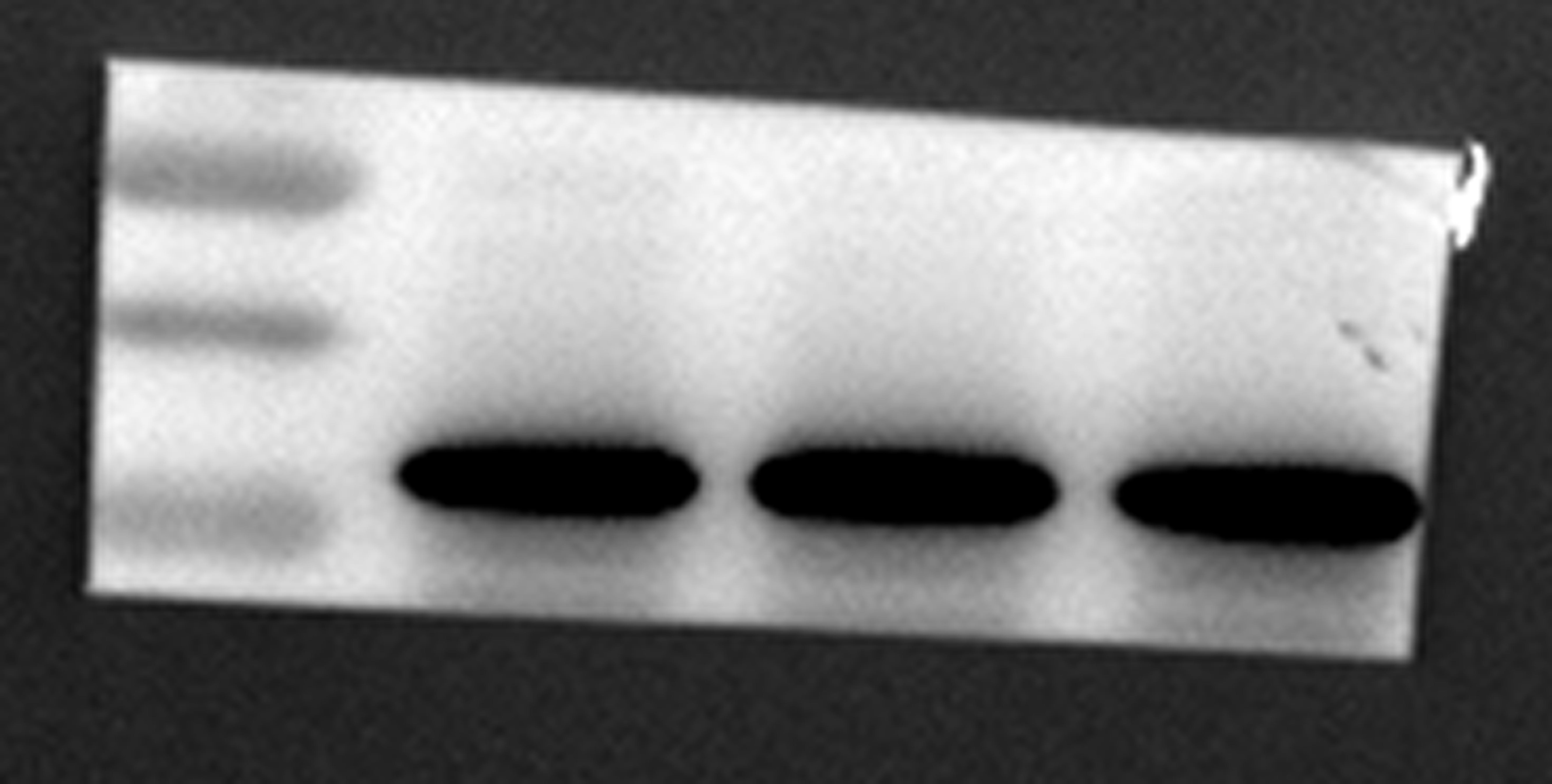

Supplement: Supplemental Material [file KBIE_A_2056692_SM9735.zip › supplementary/Figure2E_GAPDH.tif]

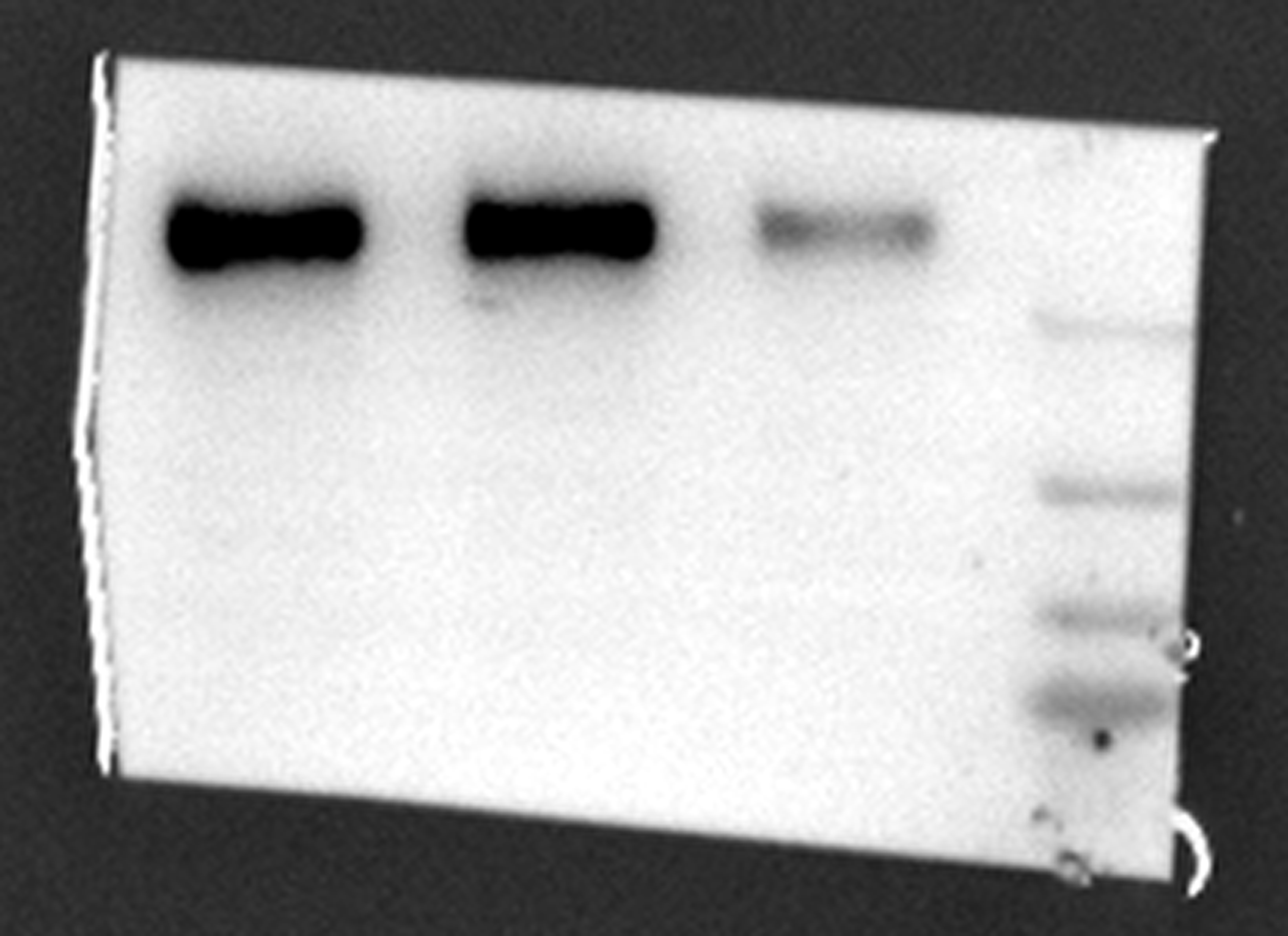

Supplement: Supplemental Material [file KBIE_A_2056692_SM9735.zip › supplementary/Figure2E_Ki67.tif]

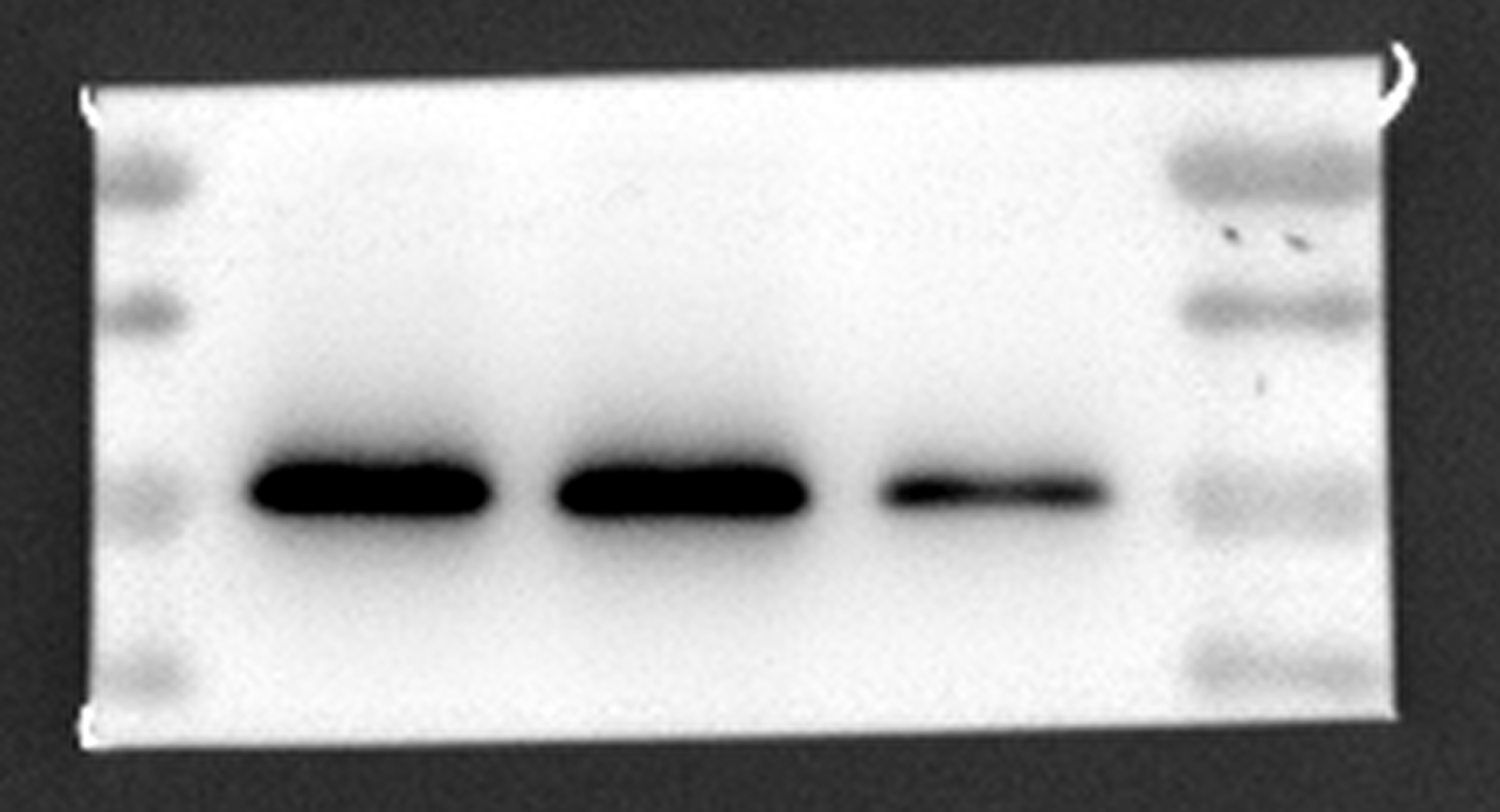

Supplement: Supplemental Material [file KBIE_A_2056692_SM9735.zip › supplementary/Figure2E_PCNA.tif]

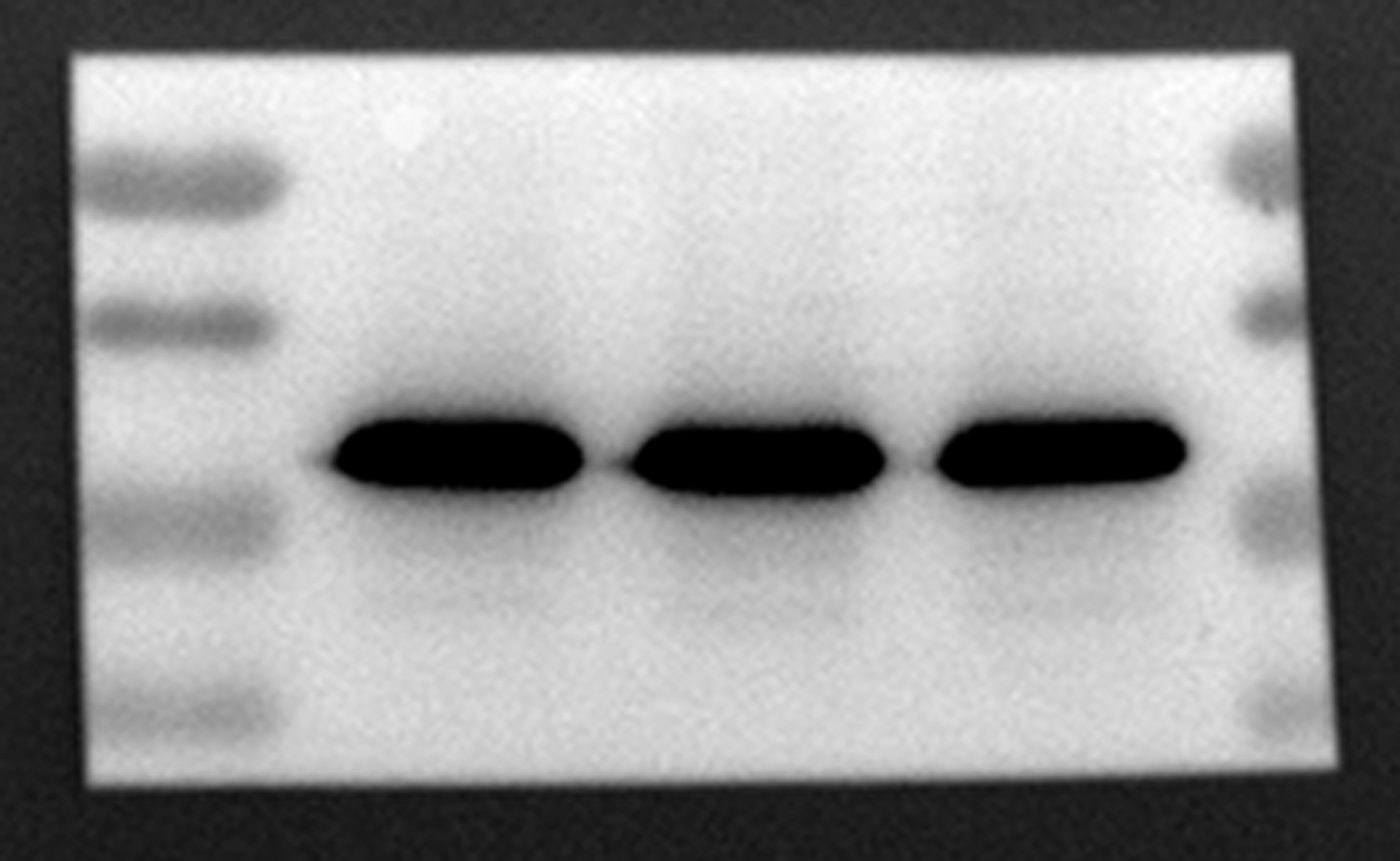

Supplement: Supplemental Material [file KBIE_A_2056692_SM9735.zip › supplementary/Figure3C_GAPDH.tif]

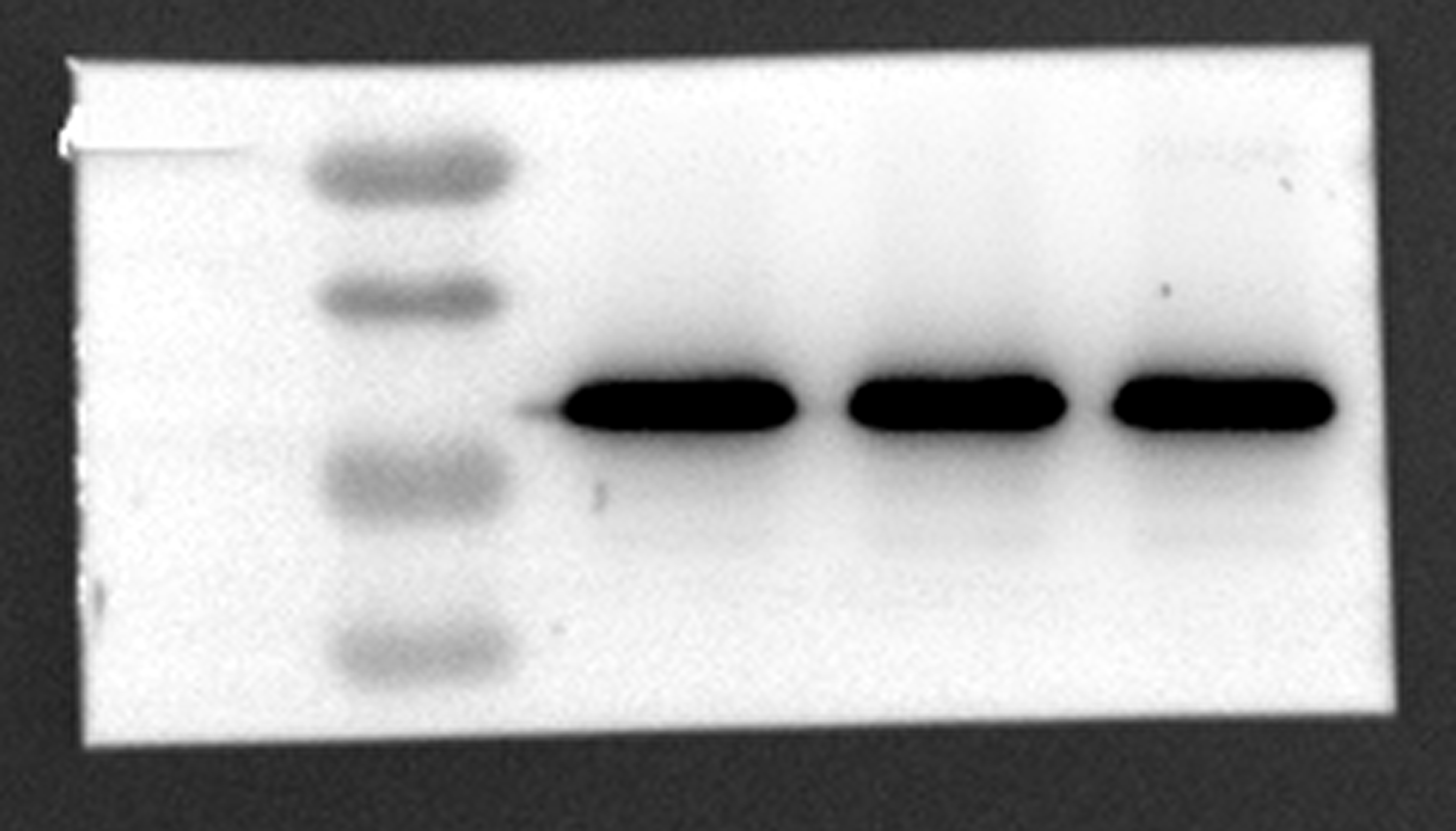

Supplement: Supplemental Material [file KBIE_A_2056692_SM9735.zip › supplementary/Figure3C_GAPDH_1.tif]

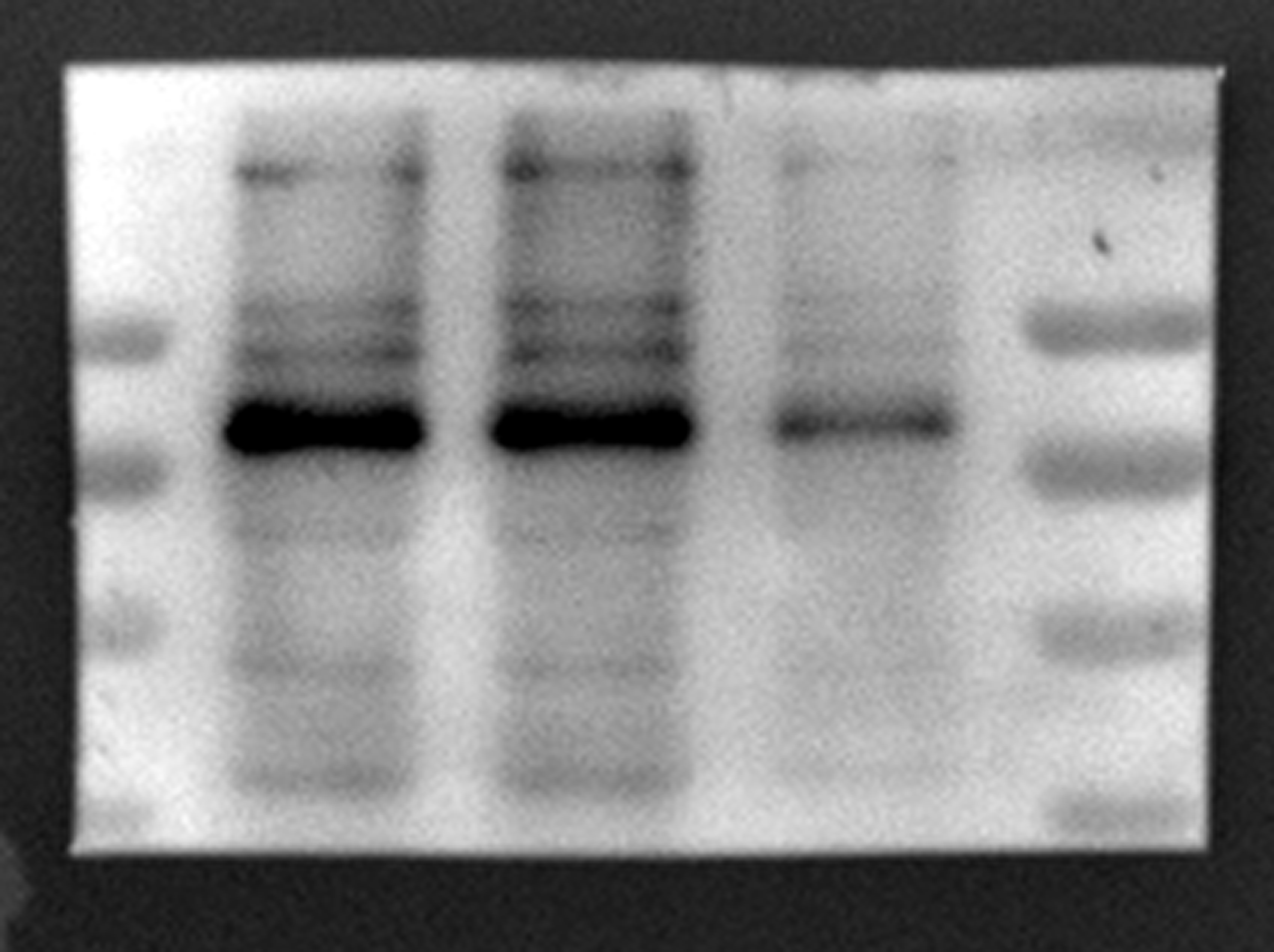

Supplement: Supplemental Material [file KBIE_A_2056692_SM9735.zip › supplementary/Figure3C_MMP2.tif]

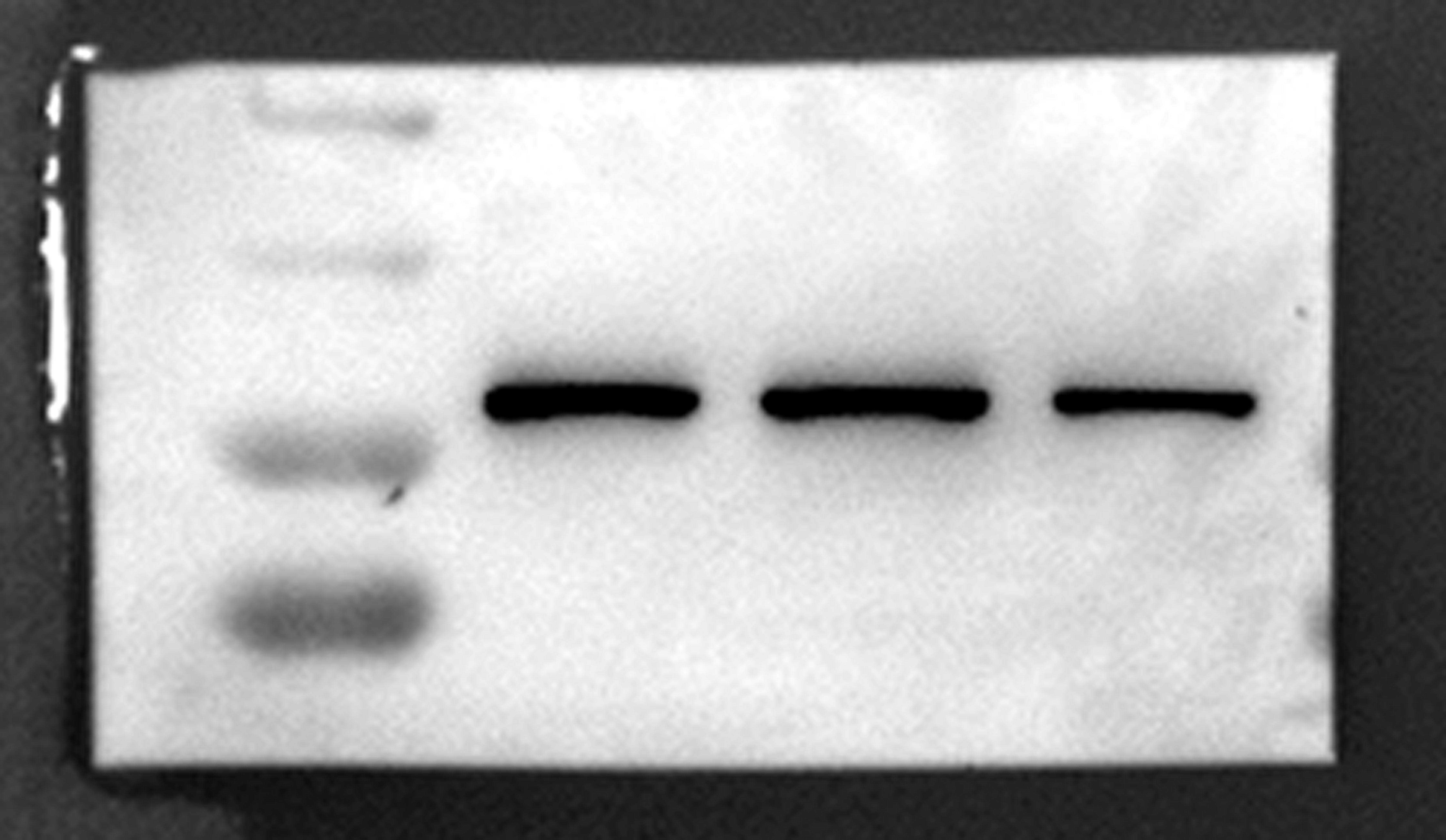

Supplement: Supplemental Material [file KBIE_A_2056692_SM9735.zip › supplementary/Figure3C_MMP9.tif]

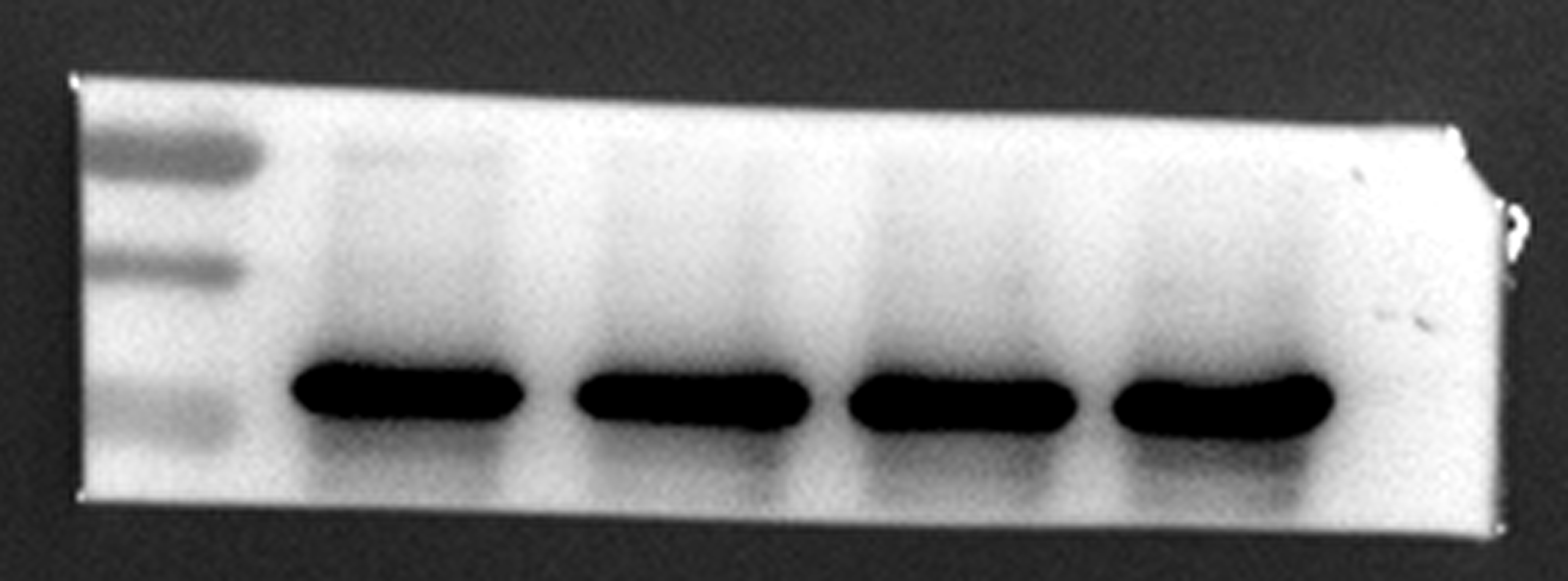

Supplement: Supplemental Material [file KBIE_A_2056692_SM9735.zip › supplementary/Figure4D_GAPDH.tif]

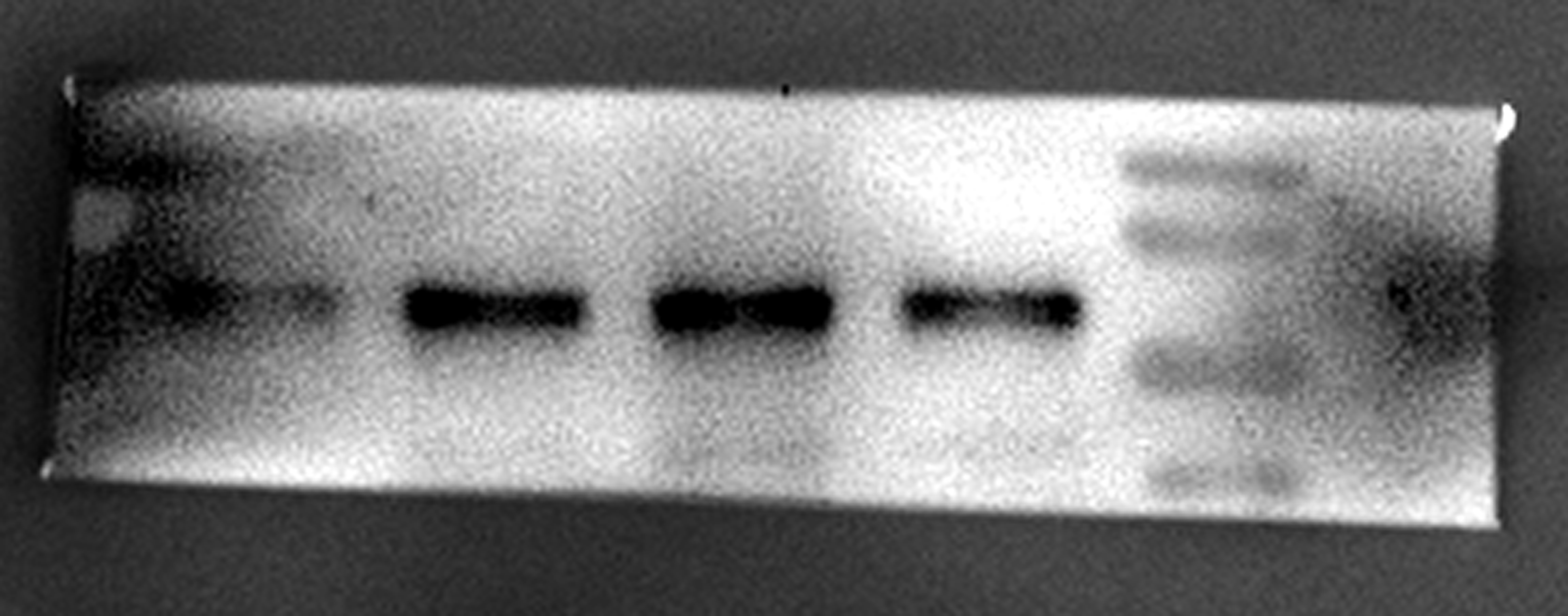

Supplement: Supplemental Material [file KBIE_A_2056692_SM9735.zip › supplementary/Figure4D_KAT5.tif]

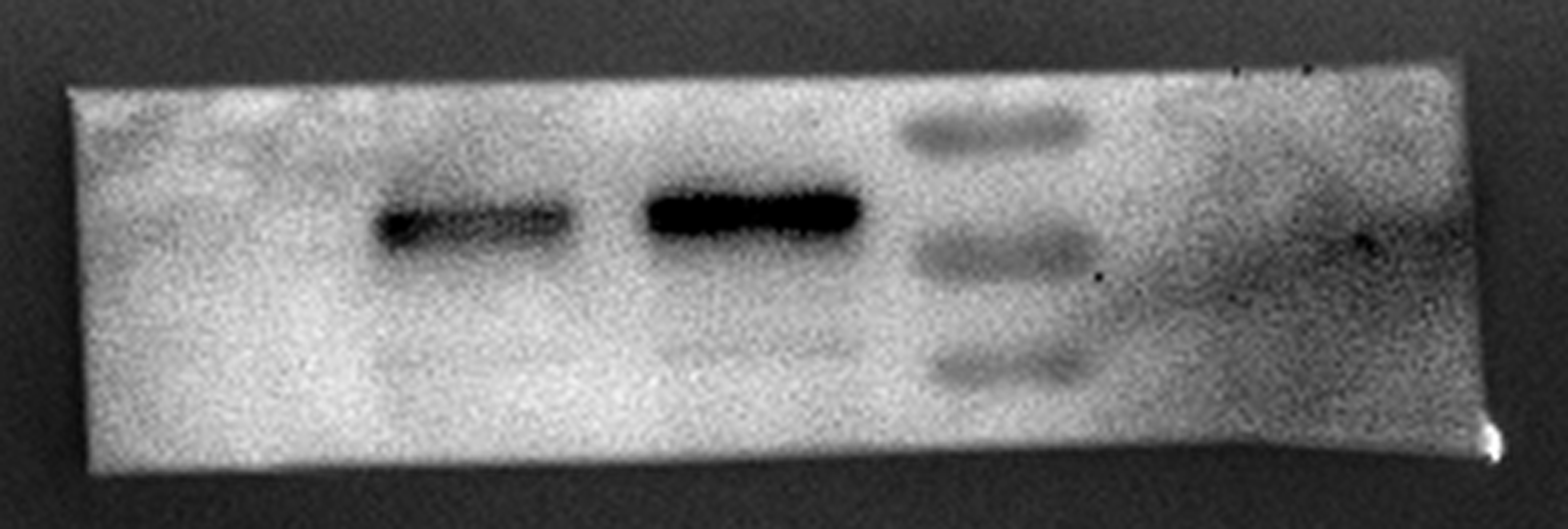

Supplement: Supplemental Material [file KBIE_A_2056692_SM9735.zip › supplementary/Figure4F_KAT5.tif]

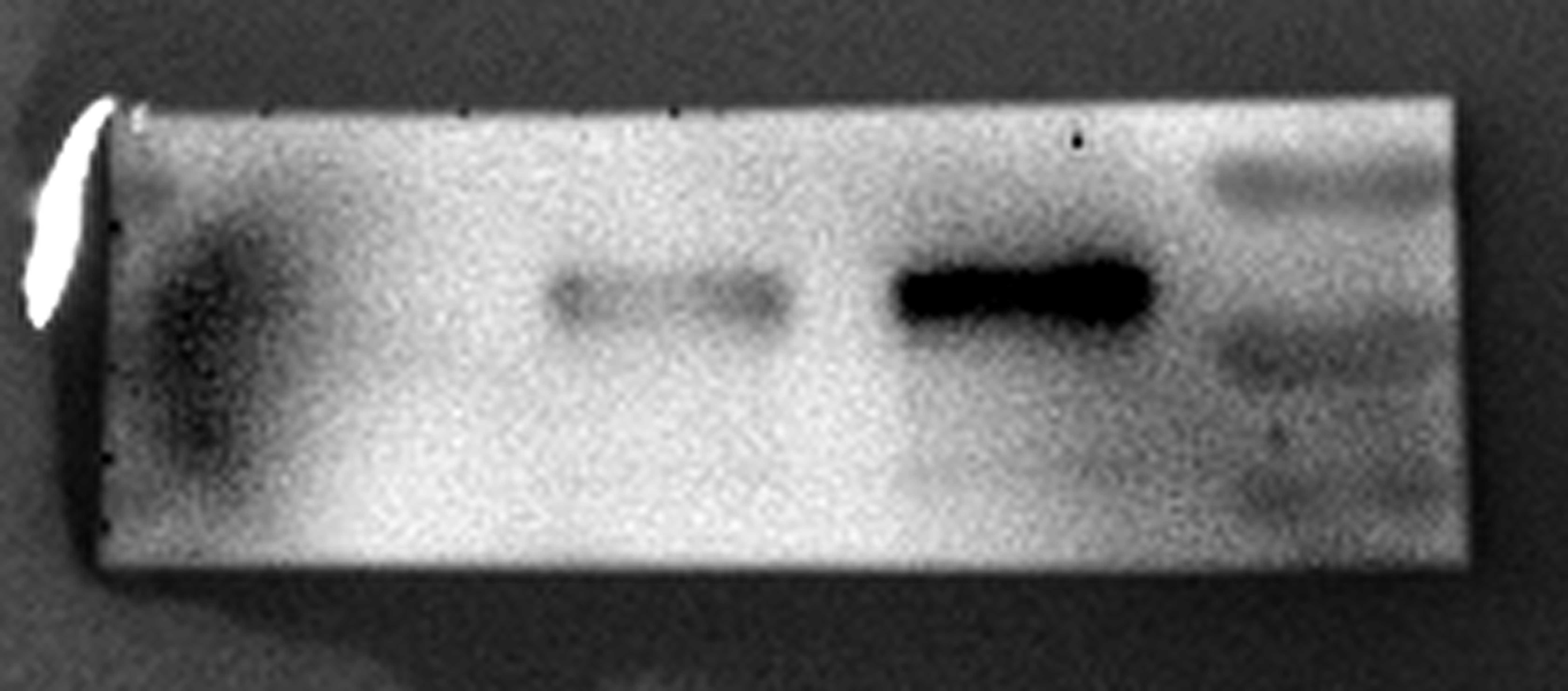

Supplement: Supplemental Material [file KBIE_A_2056692_SM9735.zip › supplementary/Figure4F_KAT5_1.tif]

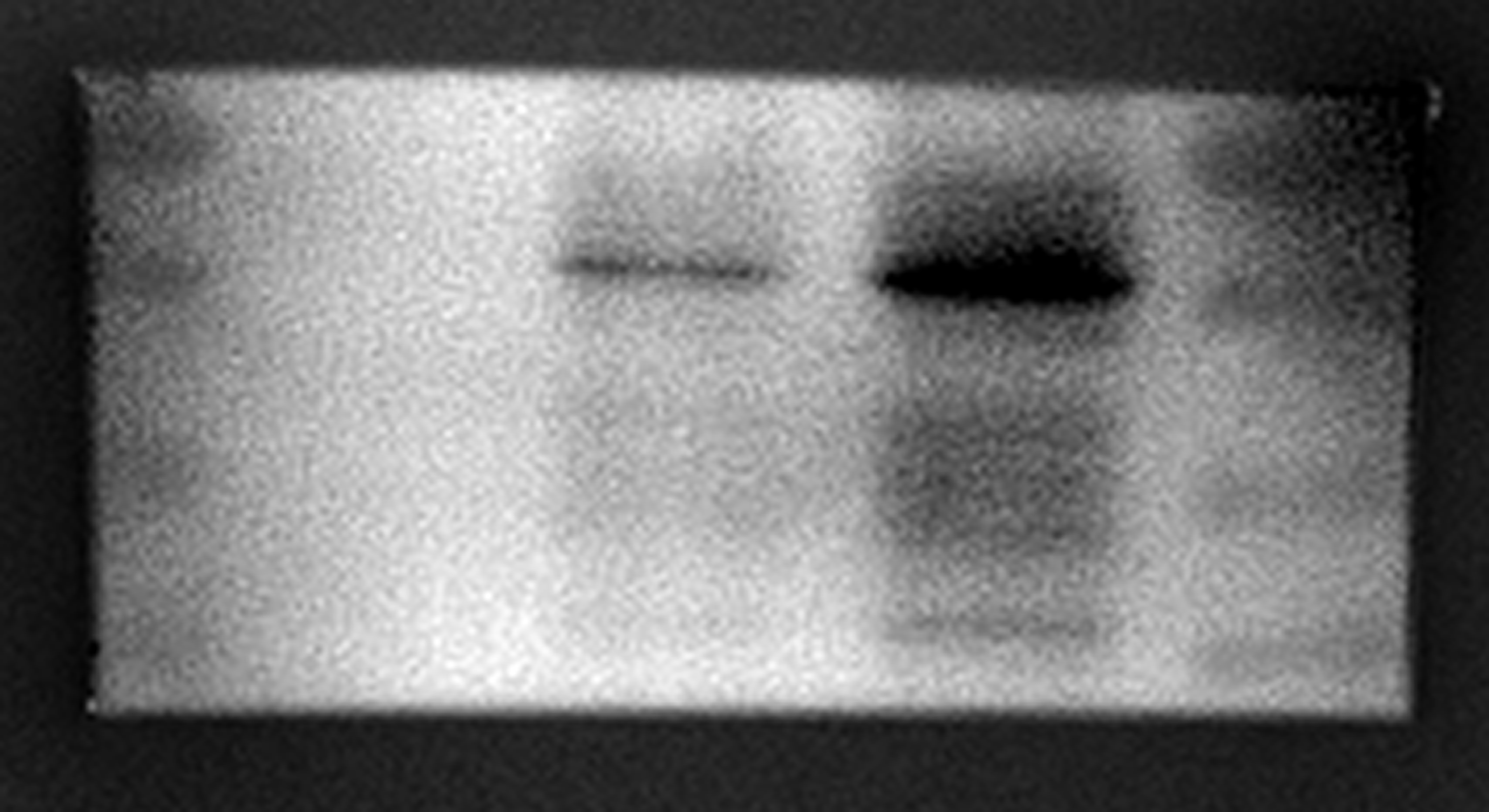

Supplement: Supplemental Material [file KBIE_A_2056692_SM9735.zip › supplementary/Figure4F_VPS72.tif]

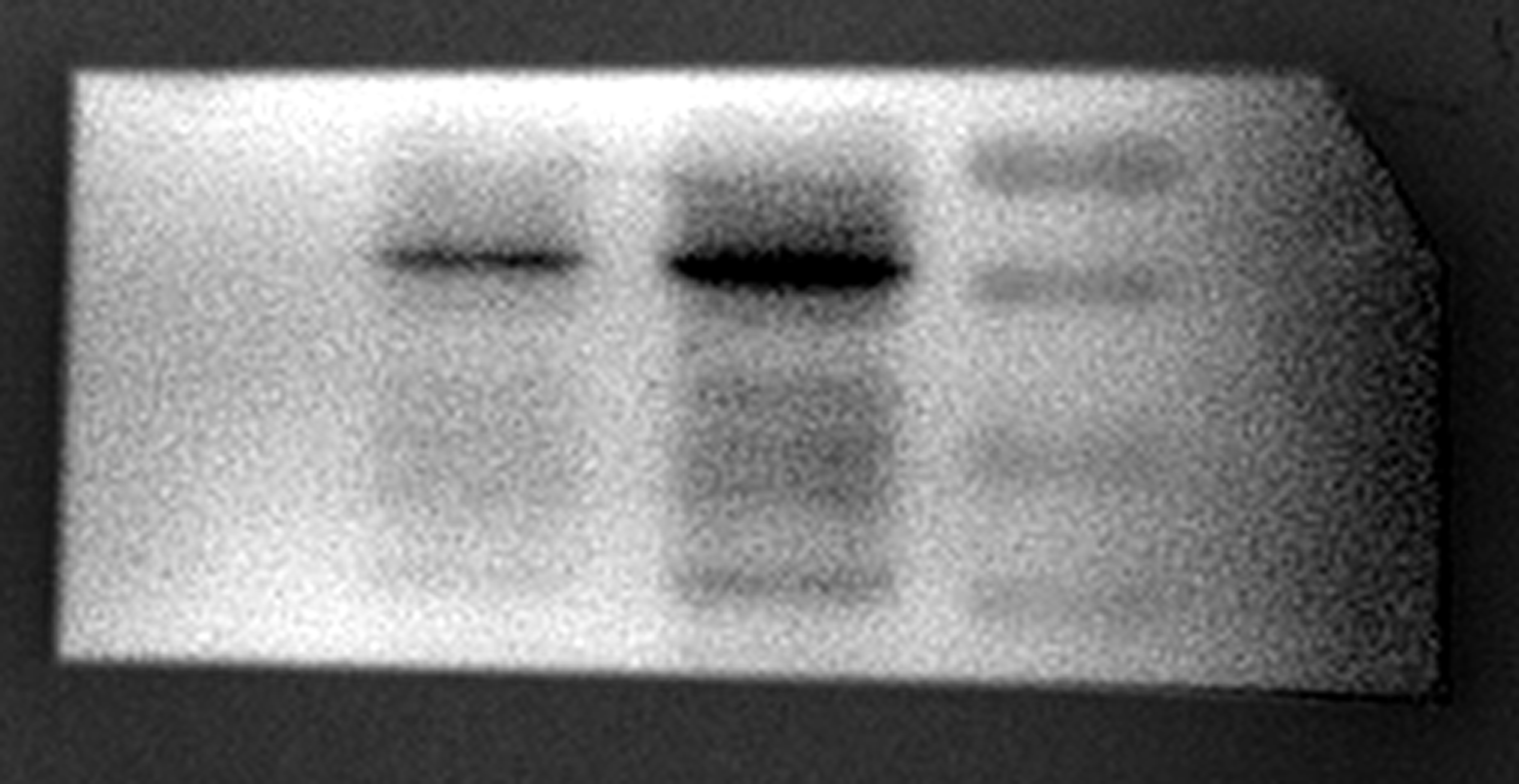

Supplement: Supplemental Material [file KBIE_A_2056692_SM9735.zip › supplementary/Figure4F_VPS72_1.tif]

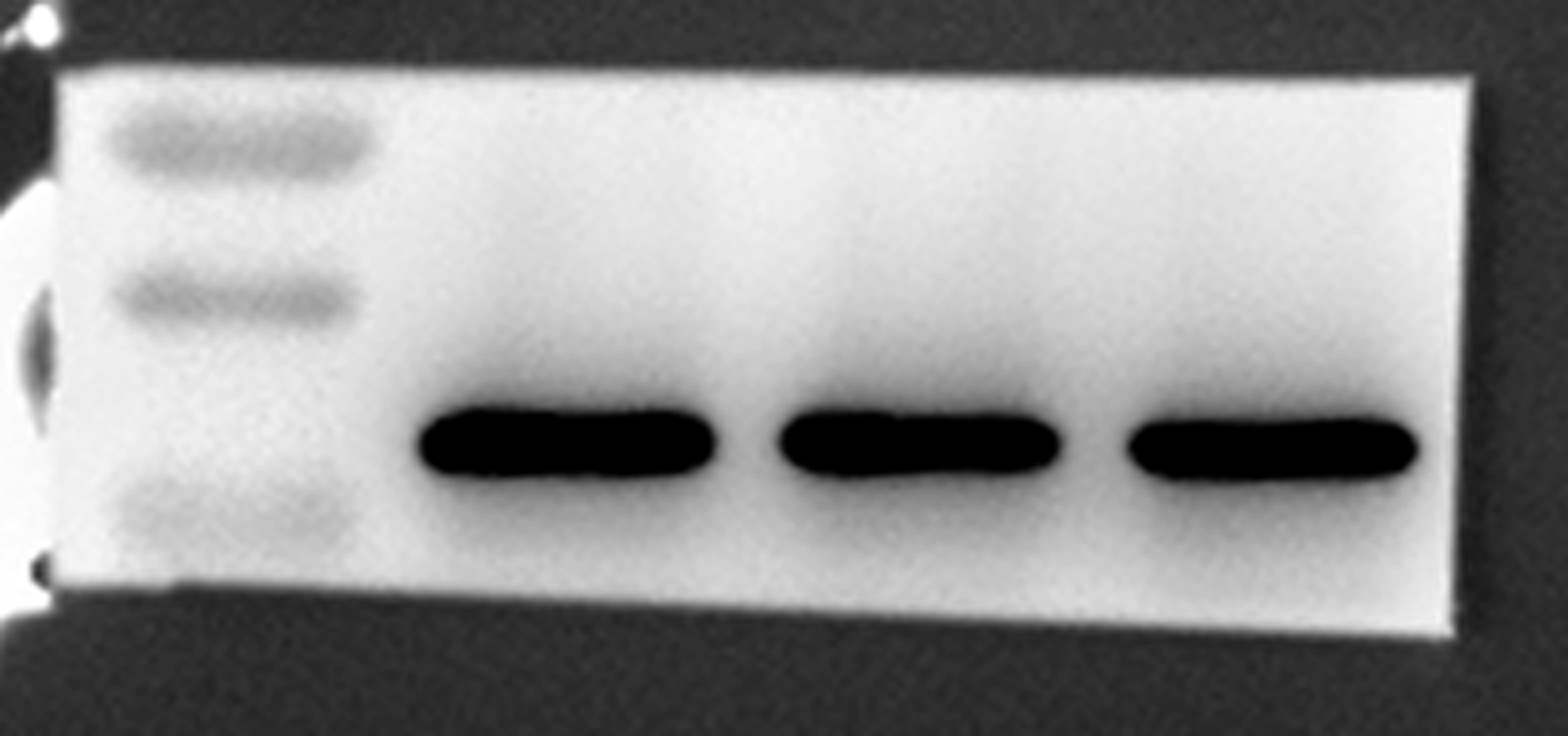

Supplement: Supplemental Material [file KBIE_A_2056692_SM9735.zip › supplementary/Figure4G_GAPDH.tif]

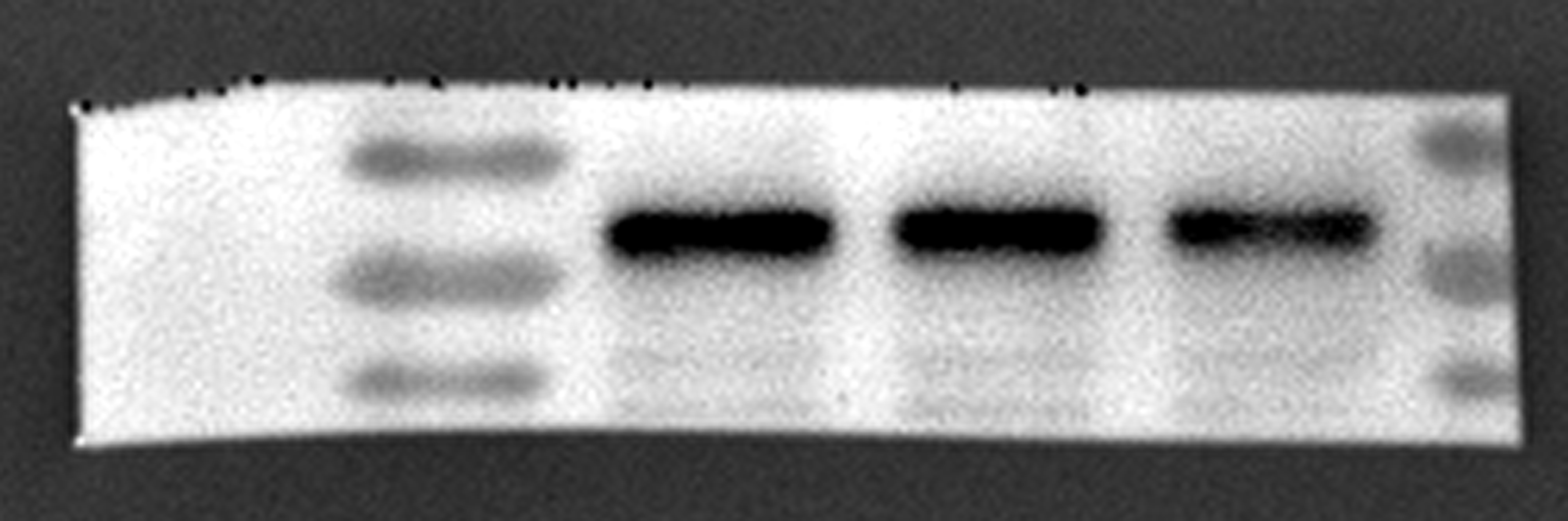

Supplement: Supplemental Material [file KBIE_A_2056692_SM9735.zip › supplementary/Figure4G_KAT5.tif]

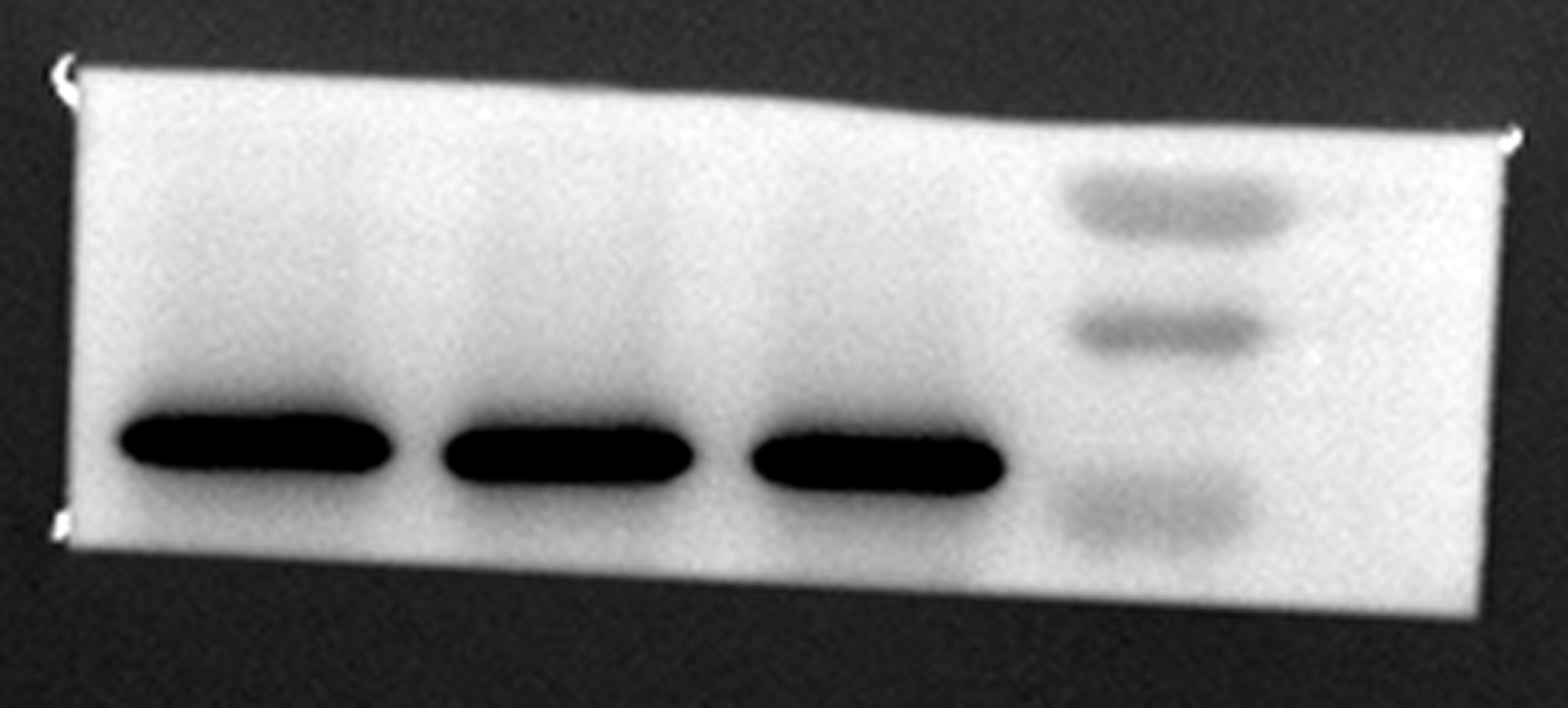

Supplement: Supplemental Material [file KBIE_A_2056692_SM9735.zip › supplementary/Figure5A_GAPDH.tif]

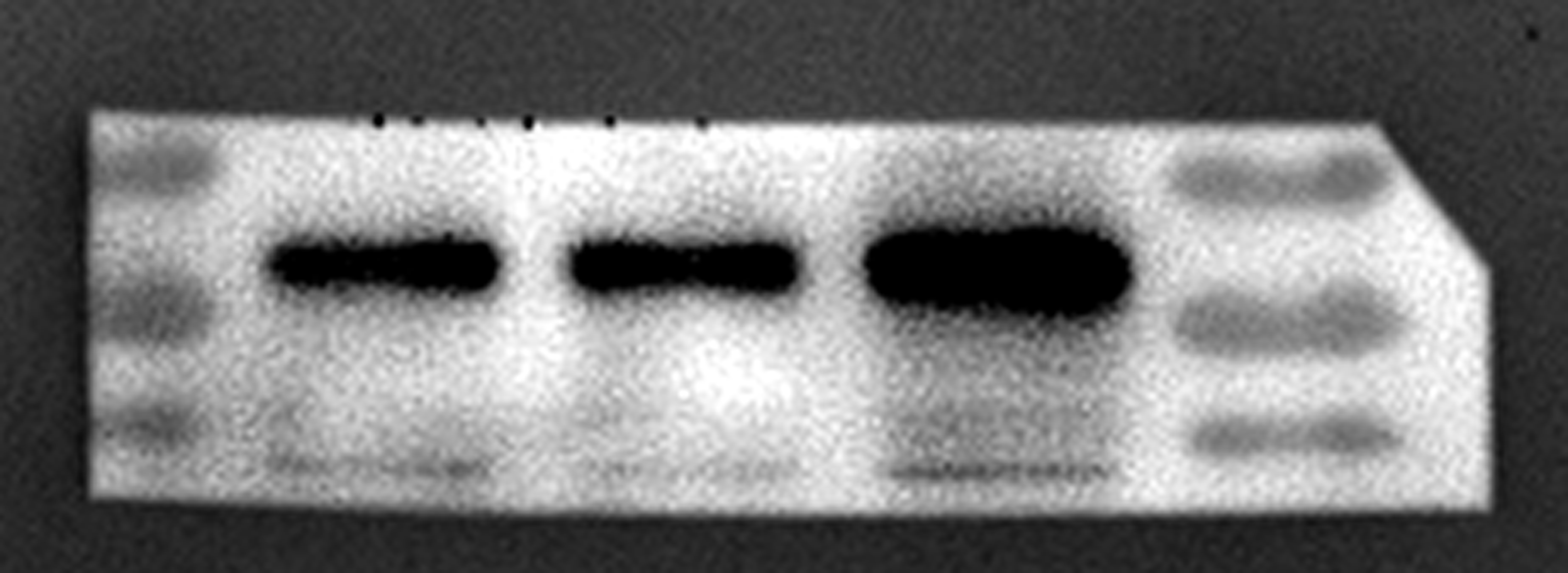

Supplement: Supplemental Material [file KBIE_A_2056692_SM9735.zip › supplementary/Figure5A_KAT5.tif]

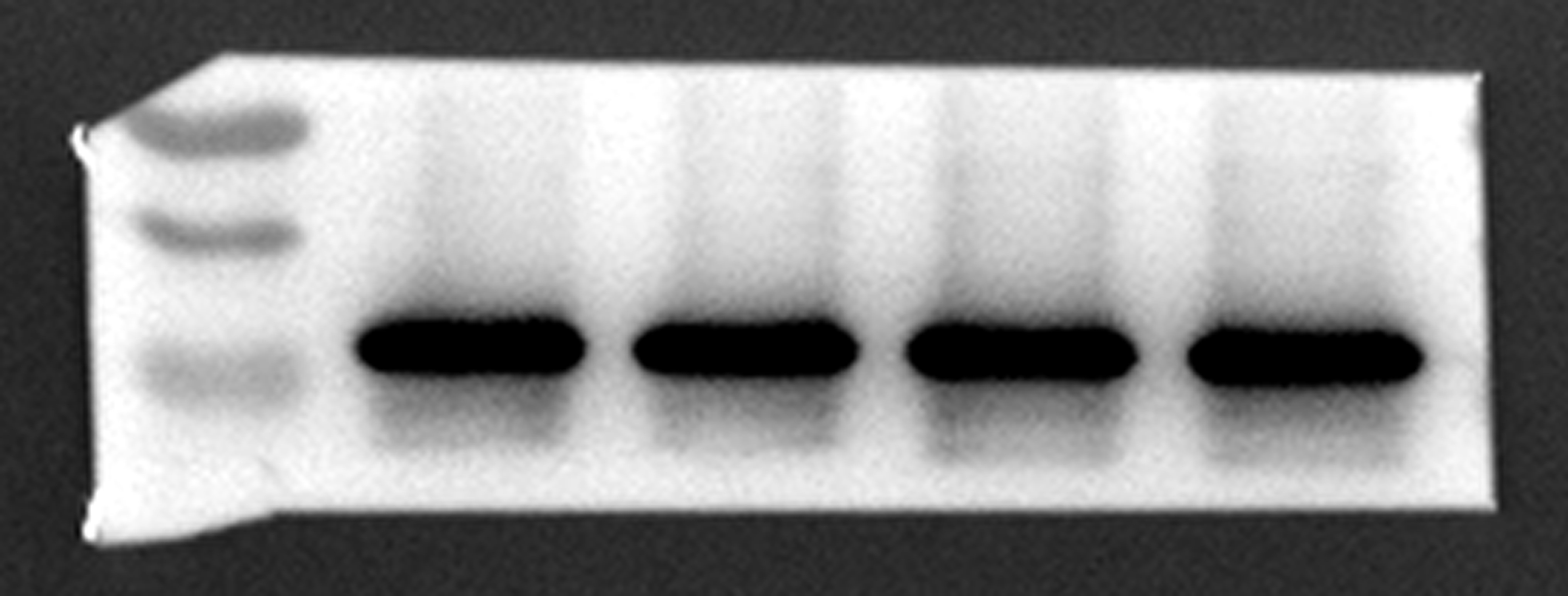

Supplement: Supplemental Material [file KBIE_A_2056692_SM9735.zip › supplementary/Figure5E_GAPDH.tif]

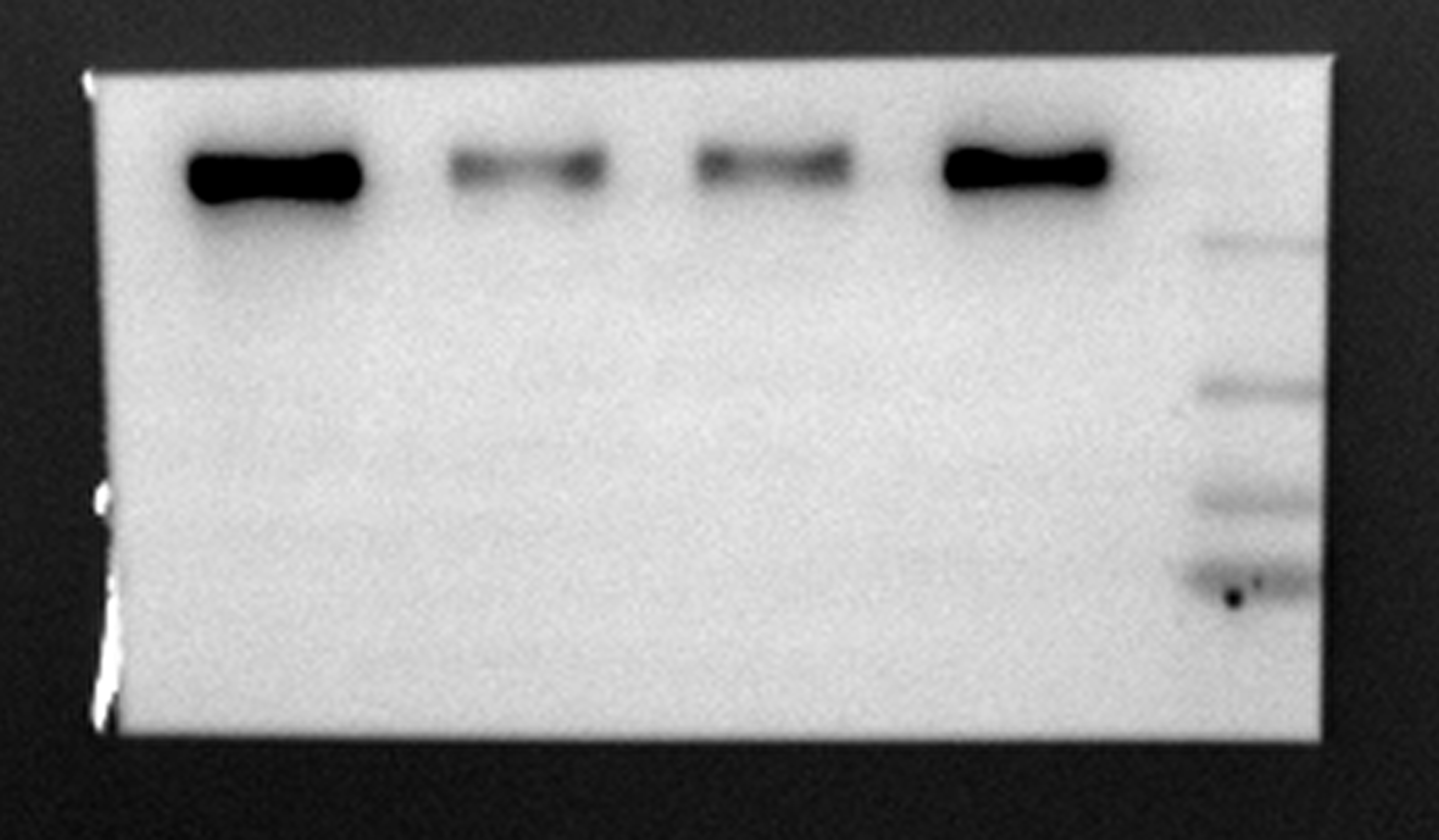

Supplement: Supplemental Material [file KBIE_A_2056692_SM9735.zip › supplementary/Figure5E_Ki67.tif]

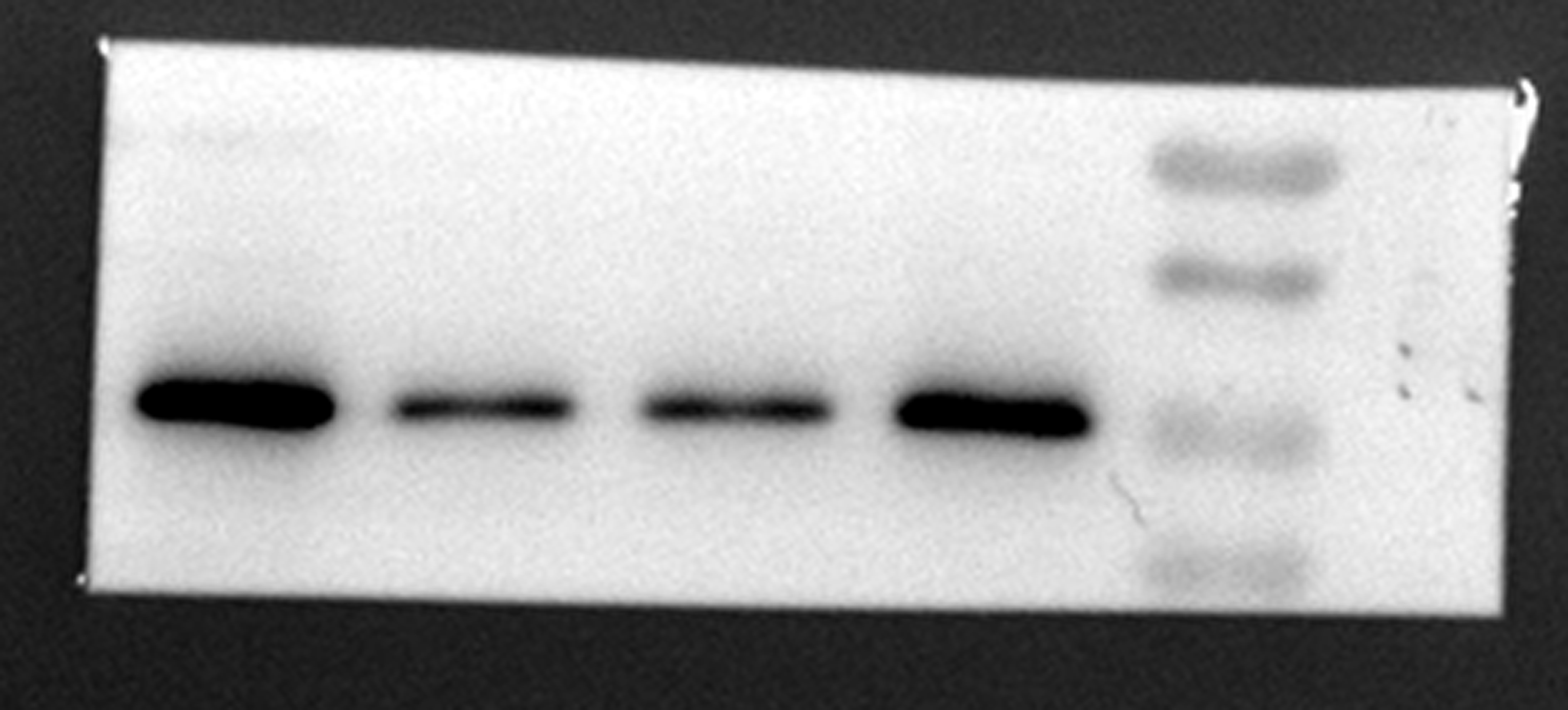

Supplement: Supplemental Material [file KBIE_A_2056692_SM9735.zip › supplementary/Figure5E_PCNA.tif]

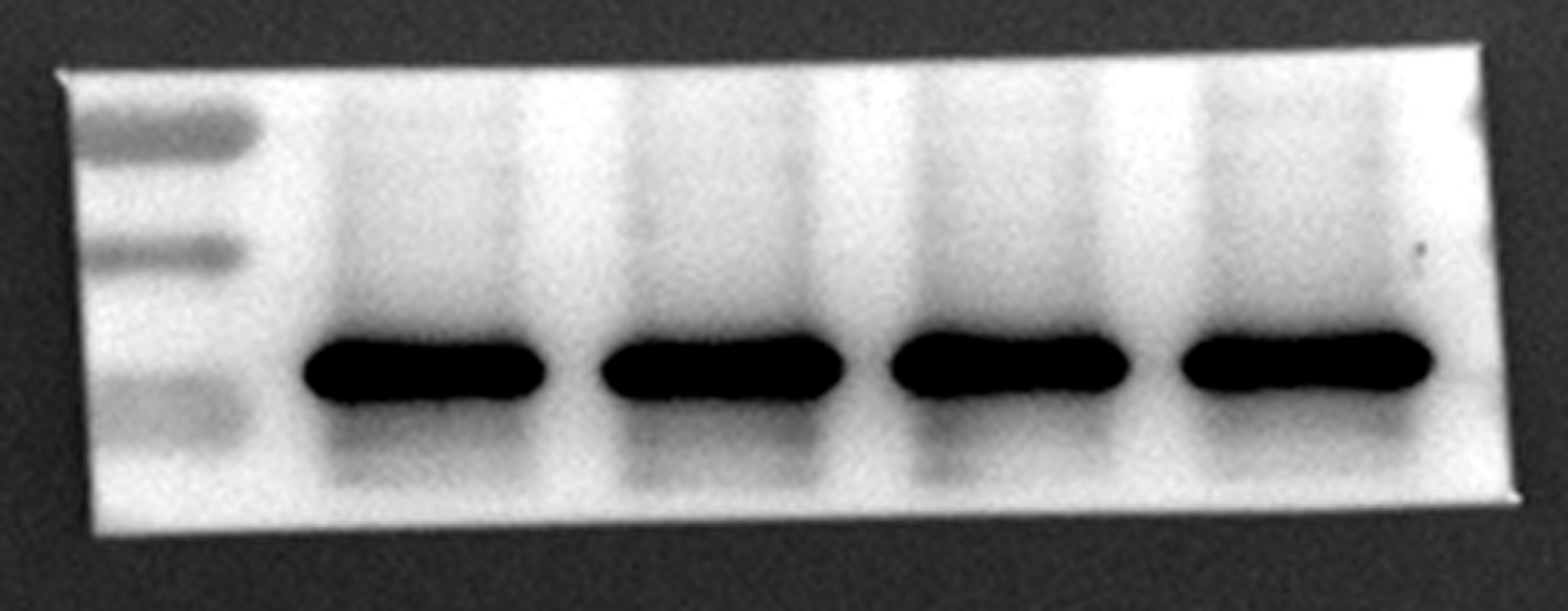

Supplement: Supplemental Material [file KBIE_A_2056692_SM9735.zip › supplementary/Figure6C_GAPDH.tif]

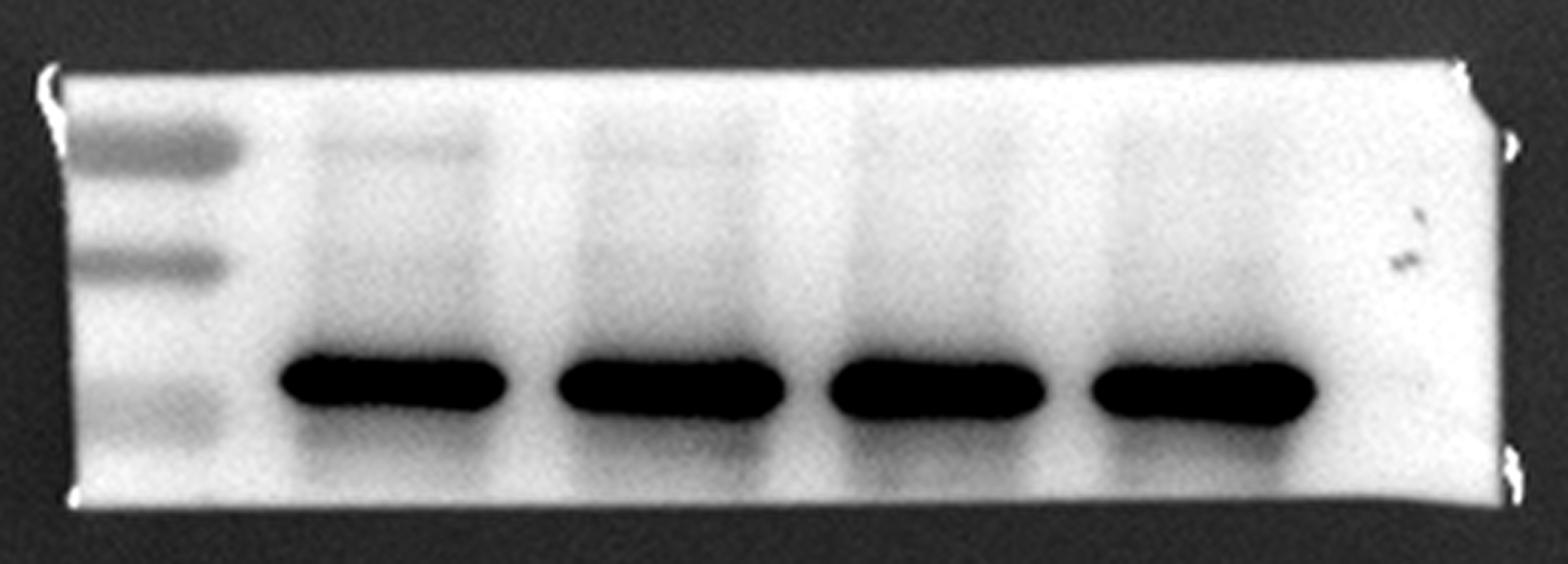

Supplement: Supplemental Material [file KBIE_A_2056692_SM9735.zip › supplementary/Figure6C_GAPDH_1.tif]

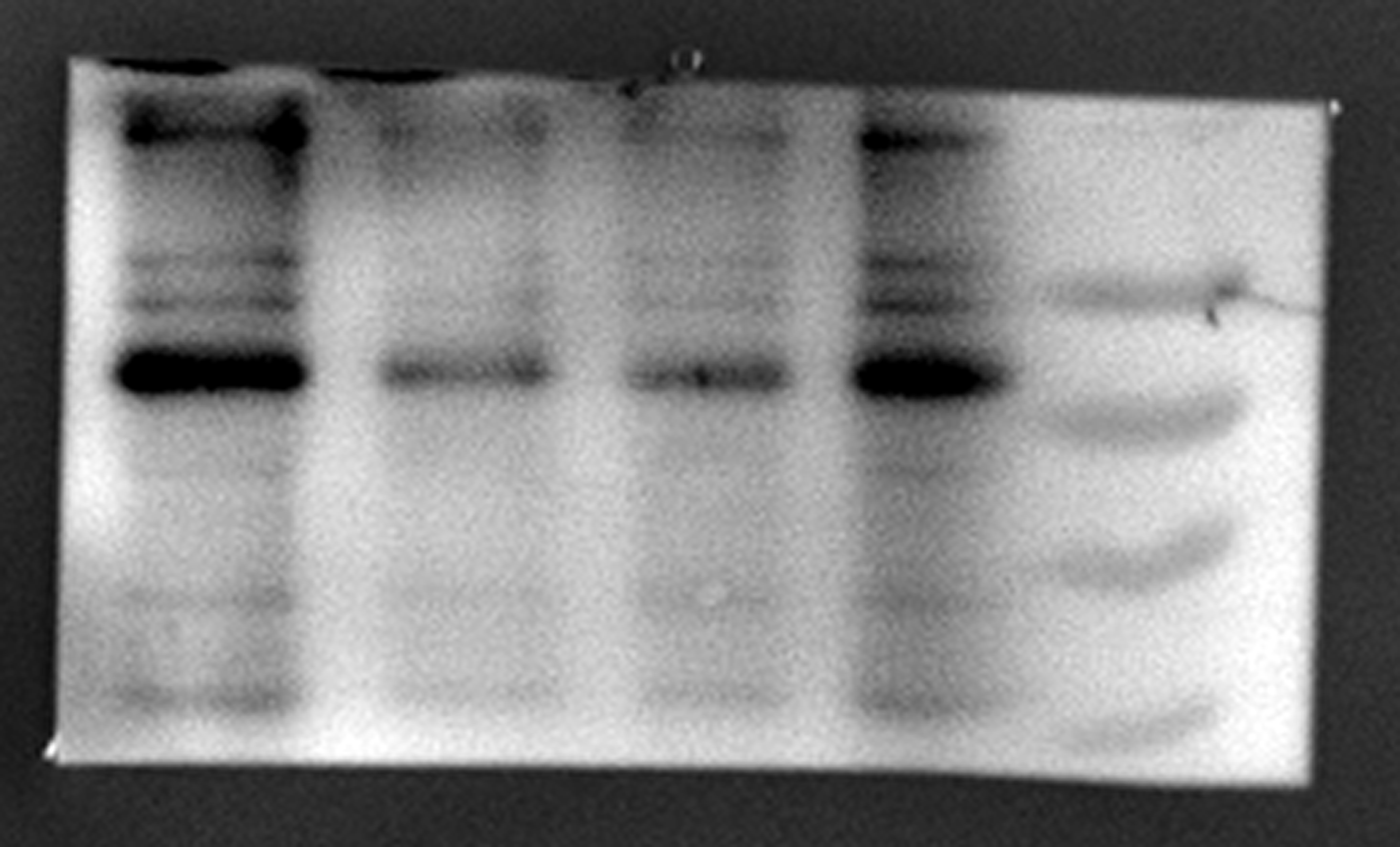

Supplement: Supplemental Material [file KBIE_A_2056692_SM9735.zip › supplementary/Figure6C_MMP2.tif]

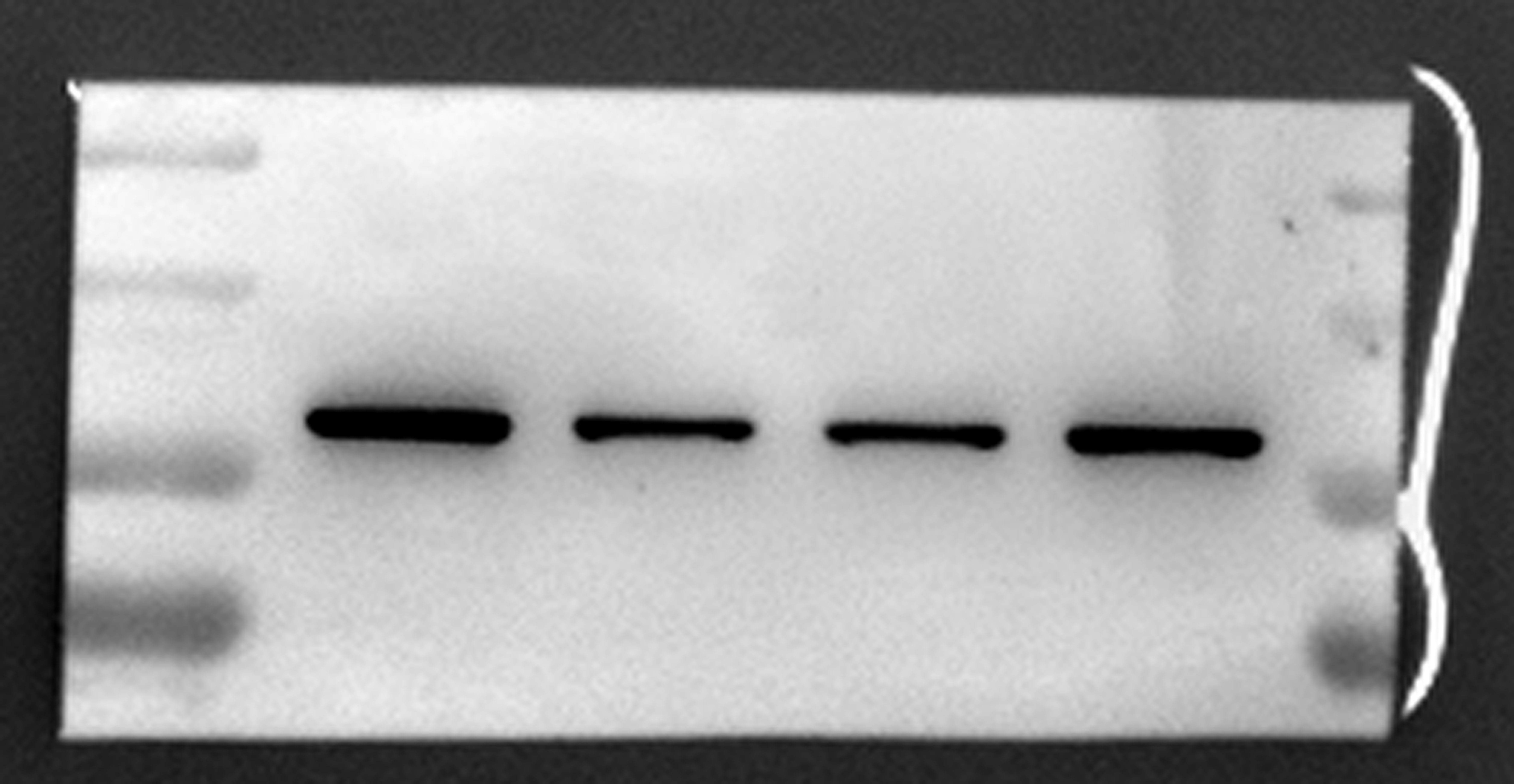

Supplement: Supplemental Material [file KBIE_A_2056692_SM9735.zip › supplementary/Figure6C_MMP9.tif]

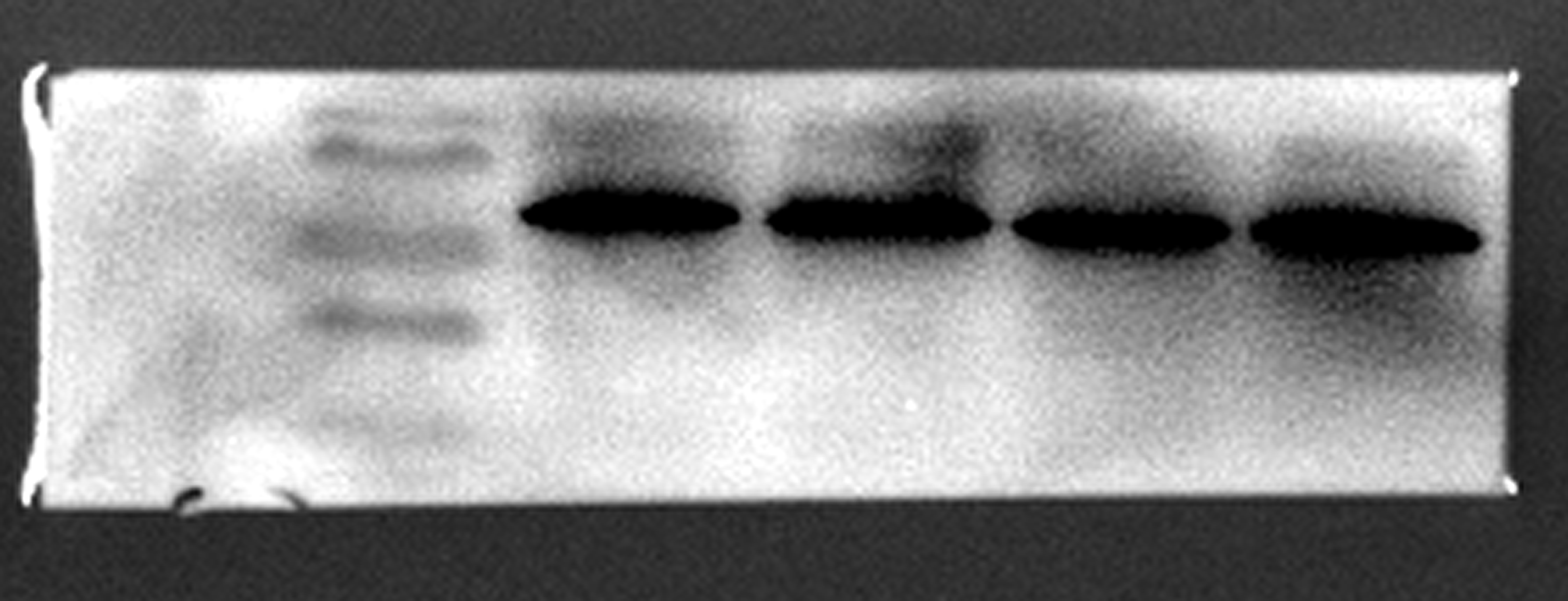

Supplement: Supplemental Material [file KBIE_A_2056692_SM9735.zip › supplementary/Figure7_AKT.tif]

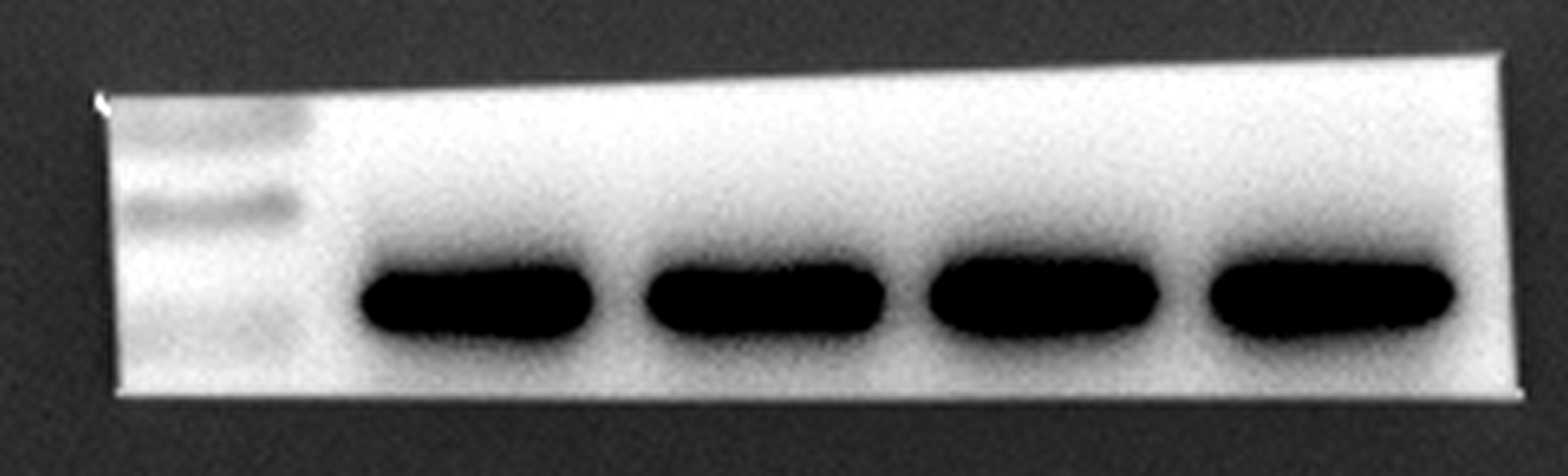

Supplement: Supplemental Material [file KBIE_A_2056692_SM9735.zip › supplementary/Figure7_GAPDH.tif]

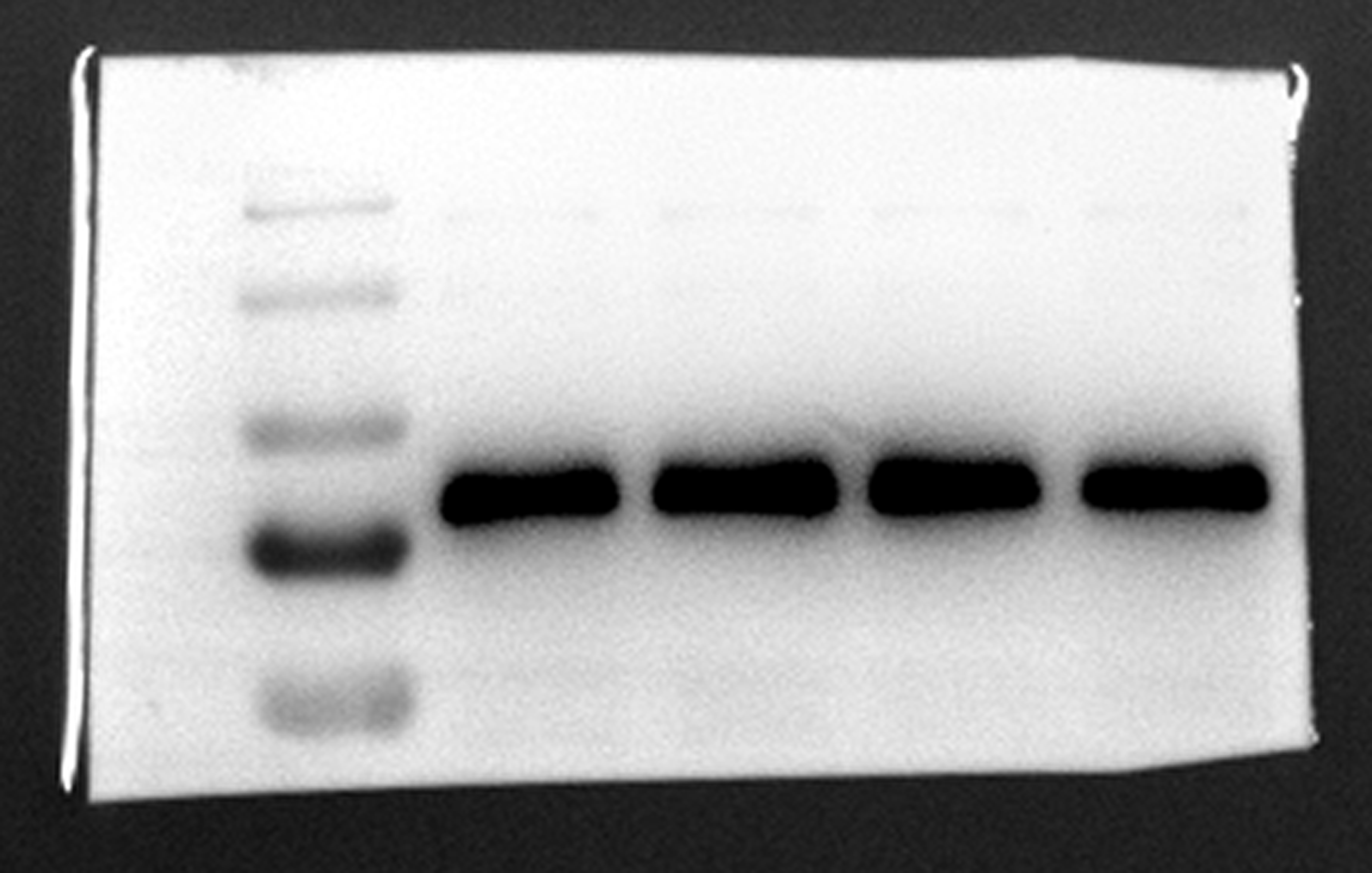

Supplement: Supplemental Material [file KBIE_A_2056692_SM9735.zip › supplementary/Figure7_PI3K.tif]

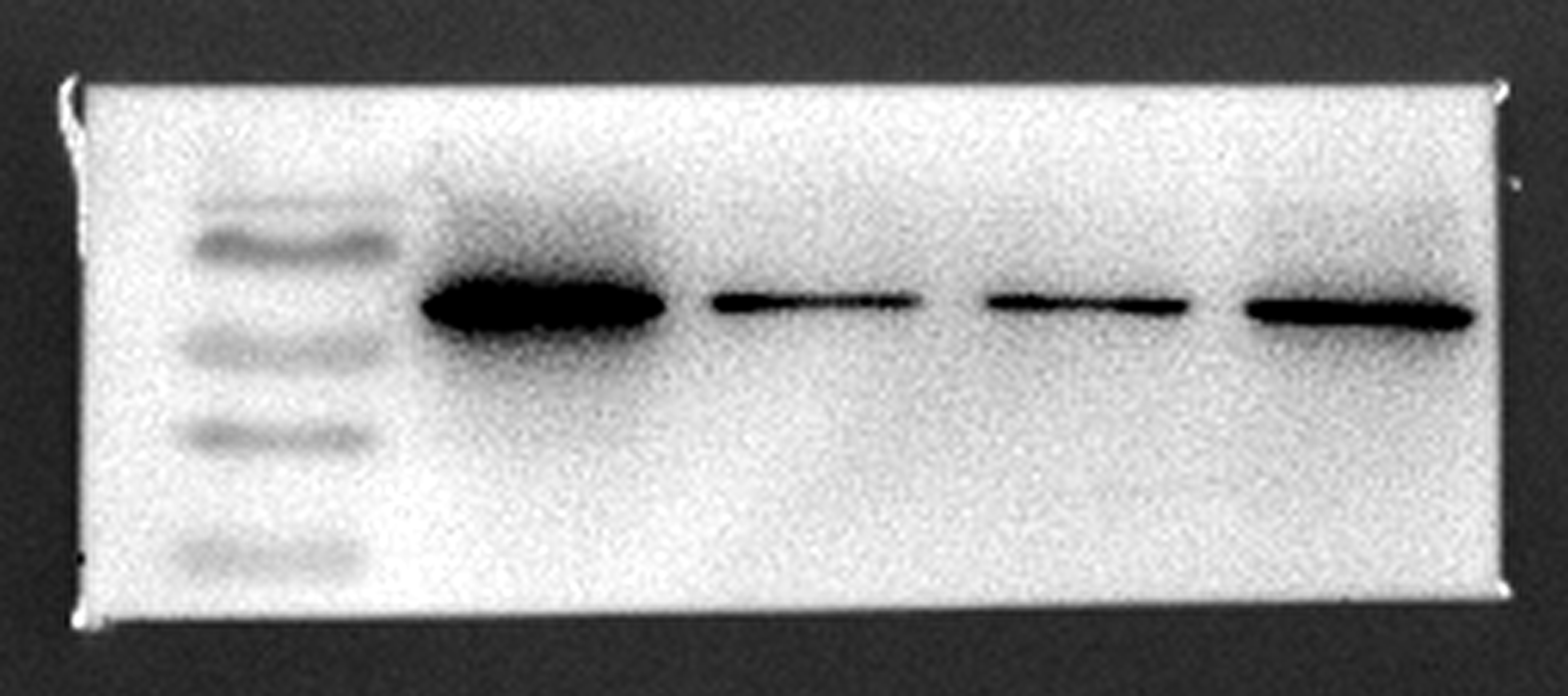

Supplement: Supplemental Material [file KBIE_A_2056692_SM9735.zip › supplementary/Figure7_p_AKT.tif]

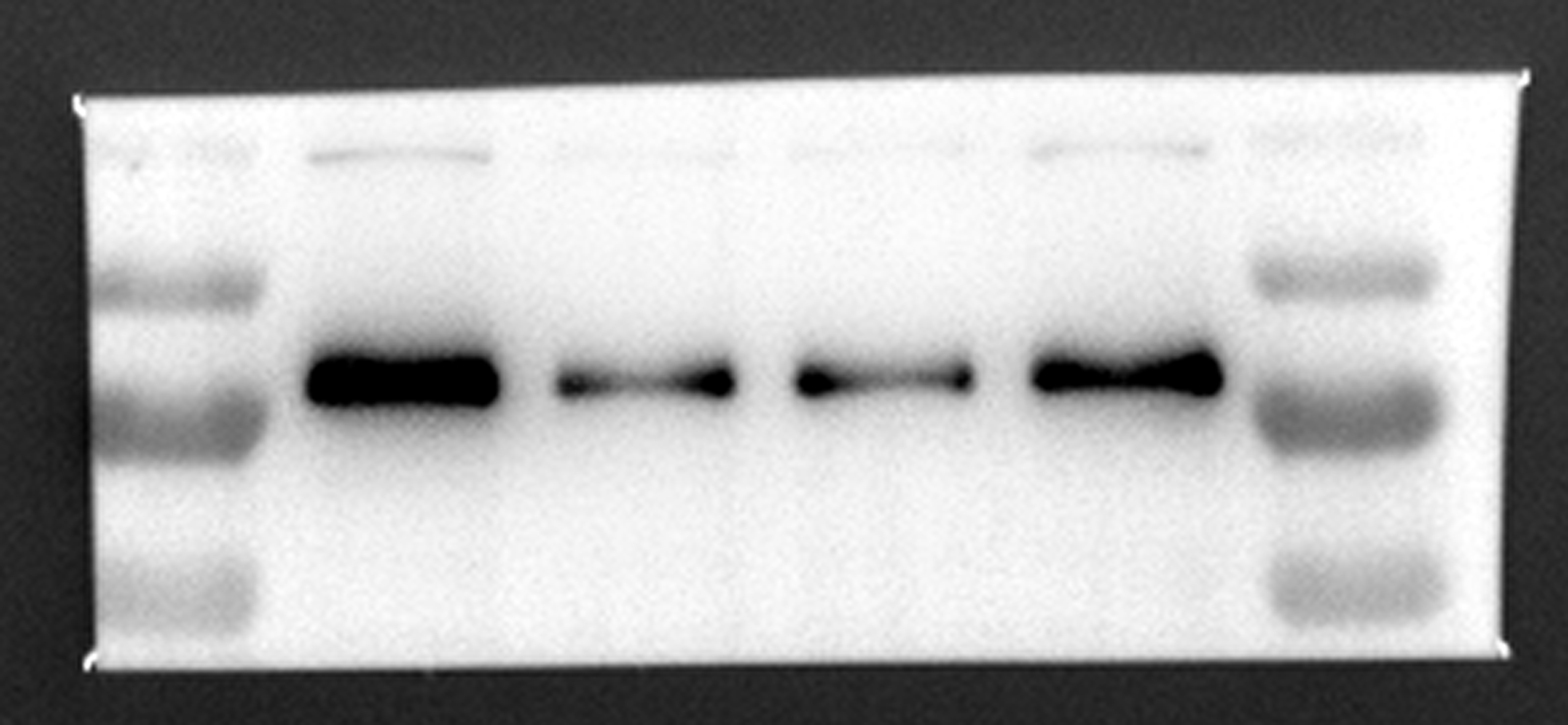

Supplement: Supplemental Material [file KBIE_A_2056692_SM9735.zip › supplementary/Figure7_p_PI3K.tif]
